# AMPK targets PDZD8 to trigger carbon source shift from glucose to glutamine

**DOI:** 10.1038/s41422-024-00985-6

**Published:** 2024-06-19

**Authors:** Mengqi Li, Yu Wang, Xiaoyan Wei, Wei-Feng Cai, Jianfeng Wu, Mingxia Zhu, Yongliang Wang, Yan-Hui Liu, Jinye Xiong, Qi Qu, Yan Chen, Xiao Tian, Luming Yao, Renxiang Xie, Xiaomin Li, Siwei Chen, Xi Huang, Cixiong Zhang, Changchuan Xie, Yaying Wu, Zheni Xu, Baoding Zhang, Bin Jiang, Zhi-Chao Wang, Qinxi Li, Gang Li, Shu-Yong Lin, Li Yu, Hai-Long Piao, Xianming Deng, Jiahuai Han, Chen-Song Zhang, Sheng-Cai Lin

**Affiliations:** 1 State Key Laboratory for Cellular Stress Biology, School of Life Sciences, Xiamen University, Xiamen, Fujian China; 2 Xiamen Key Laboratory of Radiation Oncology, Xiamen Cancer Center, The First Affiliated Hospital of Xiamen University, School of Medicine, Xiamen University, Xiamen, Fujian China; 3 Laboratory Animal Research Centre, Xiamen University, Xiamen, Fujian China; 4 School of Basic Medical Sciences, Henan University, Kaifeng, Henan China; 5 State Key Laboratory of Membrane Biology, Tsinghua University-Peking University Joint Center for Life Sciences, School of Life Sciences, Tsinghua University, Beijing, China; 6 School of Pharmacy, Zhejiang Chinese Medical University, Hangzhou, Zhejiang China; 7 Xiamen Cardiovascular Hospital of Xiamen University, School of Medicine, Xiamen University, Xiamen, Fujian China; 8 CAS Key Laboratory of Separation Science for Analytical Chemistry, Dalian Institute of Chemical Physics, Chinese Academy of Sciences, Dalian, Liaoning China

**Keywords:** Nutrient signalling, Stress signalling

## Abstract

The shift of carbon utilization from primarily glucose to other nutrients is a fundamental metabolic adaptation to cope with decreased blood glucose levels and the consequent decline in glucose oxidation. AMP-activated protein kinase (AMPK) plays crucial roles in this metabolic adaptation. However, the underlying mechanism is not fully understood. Here, we show that PDZ domain containing 8 (PDZD8), which we identify as a new substrate of AMPK activated in low glucose, is required for the low glucose-promoted glutaminolysis. AMPK phosphorylates PDZD8 at threonine 527 (T527) and promotes the interaction of PDZD8 with and activation of glutaminase 1 (GLS1), a rate-limiting enzyme of glutaminolysis. In vivo, the AMPK-PDZD8-GLS1 axis is required for the enhancement of glutaminolysis as tested in the skeletal muscle tissues, which occurs earlier than the increase in fatty acid utilization during fasting. The enhanced glutaminolysis is also observed in macrophages in low glucose or under acute lipopolysaccharide (LPS) treatment. Consistent with a requirement of heightened glutaminolysis, the PDZD8-T527A mutation dampens the secretion of pro-inflammatory cytokines in macrophages in mice treated with LPS. Together, we have revealed an AMPK-PDZD8-GLS1 axis that promotes glutaminolysis ahead of increased fatty acid utilization under glucose shortage.

## Introduction

Many physiological conditions such as fasting lead to a decline in blood glucose levels, due to the rapid depletion of stored carbohydrates, monomeric or polymeric. Nutritional adaptation is, hence, a fundamental measure to maintain energy balance.^1^ In metazoans, there are orchestrated interplays among organs and tissues to produce and redistribute alternative fuels, mainly fatty acids and amino acids.^2–8^ Fatty acids, particularly the long-chain fatty acids released from triglycerides, are first converted to fatty acyl-CoA, which is then transported into mitochondria via the carnitine palmitoyltransferase transporters (CPT1 and CPT2).^5,9–11^ Inside mitochondria, the acyl-CoA undergoes β-oxidation to generate acetyl-CoA that enters the tricarboxylic acid (TCA) cycle to produce energy.^12–14^ Among amino acids, glutamine is the most abundant circulating amino acid, comprising more than 50% of the free amino acid pool in the body during starvation. It serves as a key alternative carbon source.^15–18^ It is known that glutamine, along with alanine, is converted from other amino acids, particularly the branched-chain amino acids, derived from muscle protein breakdown under starvation.^18–20^ While alanine mainly contributes to hepatic gluconeogenesis in the liver, glutamine is utilized in various tissues to directly meet energy demand,^16,19–21^ as well as for gluconeogenesis in the liver, intestine, and kidney.^22–27^ In addition, glutamine can also act as a major source of GSH and NADPH synthesis during starvation to maintain the cellular redox state.^28–32^

AMPK plays a central role in maintaining energy homeostasis, mainly through phosphorylating multiple targets to stimulate catabolism and inhibit anabolism, thereby promoting ATP production and reducing ATP consumption.^33^ In addition to its classic role as an energy sensor regulated by increased AMP and ADP levels,^34,35^ AMPK is highly sensitive to activation by falling glucose levels,^36^ even independent of a decrease of cellular energy status.^37^ In this, it is the declining levels of glycolytic intermediate fructose-1,6-bisphosphate (FBP) that trigger activation of lysosomally localized AMPK by the upstream kinase LKB1 via the glucose-sensing pathway comprising aldolase (direct sensor for the presence or absence of FBP^37^), transient receptor potential V (TRPV), vacuolar H^+^-ATPase (v-ATPase), Ragulator and AXIN.^38–40^ Upon activation by the glucose-sensing axis, AMPK phosphorylates acetyl-CoA carboxylase 1 (ACC1),^41^ which inhibits the production of malonyl-CoA to remove the inhibition of CPT1, thereby promoting the transport of acyl-CoA into mitochondria and fatty acid oxidation (FAO).^42^ AMPK also promotes catabolism of amino acids by inhibiting translation, either through inhibiting the target of rapamycin complex 1 (TORC1),^43,44^ or through promoting the inhibition of the eukaryotic elongation factor 2 (eEF2) by eEF2 kinase (eEF2K).^45^ In addition, AMPK helps release free amino acids from cellular proteins either by promoting autophagy,^46–48^ or through increasing proteasomal degradation of labile proteins.^49^ However, the mechanisms underlying the prioritization of alternative carbon source utilization remain unclear.

Here, we show that in physiologically low glucose, rapid promotion of glutamine utilization (glutaminolysis) occurs before the promotion of FAO, and that AMPK is required for the increased glutaminolysis. In the course of studying the effect of glucose starvation on mitochondria, we observed that glucose starvation induces an increase in mitochondria-associated membrane (MAM). Through proteomic analysis of the proteins pulled down from MAM by an antibody against pan-AMPK phosphoproteins, we identified that PDZD8, an endoplasmic reticulum (ER)-localized protein, is a new substrate of AMPK. We further show that AMPK-mediated phosphorylation of PDZD8 is required for the increase of glutaminolysis to compensate for the scarcity of glucose before the promotion of FAO. We also demonstrate that phosphorylated PDZD8 interacts with and activates GLS1 to enhance glutaminolysis. In mice, we found that the AMPK-PDZD8-GLS1 axis is required for the enhancement of glutaminolysis in the skeletal muscle and macrophages when glucose levels are low. The increased glutaminolysis also plays a crucial role in the release of pro-inflammatory cytokines by macrophages during acute lipopolysaccharide (LPS) treatment that leads to a rapid decrease in blood glucose. In short, we have elucidated the molecular mechanism underlying the carbon source shift from glucose to glutamine.

## Results

### Glutamine is the alternative carbon source during early starvation

**Fig. 1:**
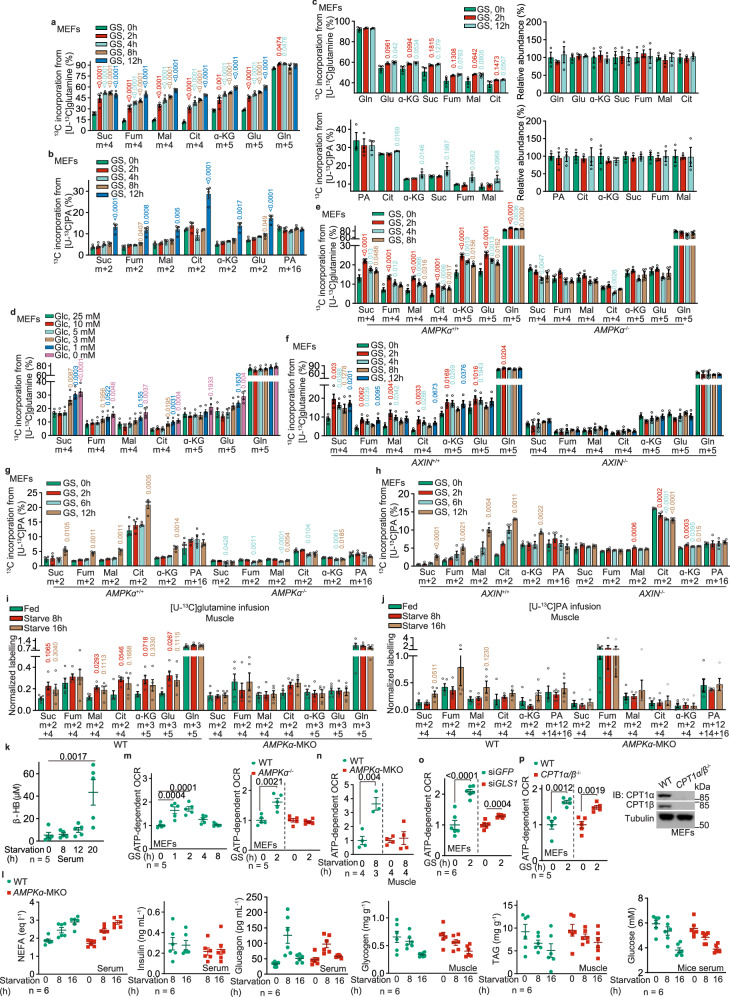
AMPK promotes glutaminolysis before promoting FAO in low glucose. **a**, **b**, **d** Glutaminolysis is promoted ahead of the increase of FAO under low glucose. MEFs were glucose starved (GS) for desired durations (**a**, **b**), or incubated with a medium containing desired concentrations of glucose (**d**). At 20 min and 12 h before sample collection, cells were labeled with [U-^13^C]-glutamine (**a**, **d**) and [U-^13^C]-PA (**b**), respectively, followed by determination of the levels of labeled TCA cycle intermediates, including succinate (Suc), fumarate (Fum), malate (Mal), citrate (Cit), α-ketoglutarate (α-KG), along with glutamate (Glu), by gas chromatography-mass spectrometry (GC-MS). Levels of m + 5 α-ketoglutarate and glutamate; and m + 4 succinate, fumarate, malate, and citrate that reflect the rates of glutaminolysis (**a**, **d**), along with levels of m + 2 α-ketoglutarate, glutamate, succinate, fumarate, malate and citrate that reflect the rates of FAO (**b**), were shown. See also Supplementary information, Fig. S1b, c, f for the levels of other isotopomers of the labeled metabolites shown in (**a**, **b**, and **d**). Data are shown as mean ± SEM; *n* = 4 samples for each condition; *P* values were determined by one-way ANOVA, followed by Dunnett (**d**), Dunn (fumarate, malate, and α-KG of **b**), or Sidak (others). *P* values labeled in these panels represent the comparisons between the starved and the unstarved groups; same hereafter. **c** Glutamine utilization compensates for the reduction of glucose oxidation in the TCA cycle in low glucose. MEFs were separately labeled with [U-^13^C]-glutamine and [U-^13^C]-PA, all for 24 h, followed by glucose starvation for 2 h and 12 h. Show here are the relative contributions of each carbon source to the TCA cycle, as calculated by the total levels of labeled TCA cycle intermediates: ((m + 1) × 1 + (m + 2) × 2 + …… (m + n) × n)/n, in which n represents the number of labeled carbon numbers of each intermediate. See also abundance (pool size; calculated as (m + 0) + (m + 1) + (m + 2) + …… (m + n); all normalized to the unstarved group) of each TCA cycle intermediate on the right panels. Data are shown as mean ± SEM; *n* = 3 samples for each condition; *P* values were determined by one-way ANOVA, followed by Dunn (citrate of the upper left panel, and α-KG of lower left panel), Sidak (succinate, fumarate and malate of lower left panel), or Dunn (others). **e**–**h** AMPK promotes the utilization of glutamine during early starvation in MEFs. Experiments in **e** and **f** (for determining glutaminolysis) were performed as in **a**, and those in **g** and **h** (for determining FAO) as in **b**) except that *AMPKα*^–/–^ MEFs (**e**, **g**), *AXIN*^–/–^ MEFs (**f**, **h**) were used. Data are shown as mean ± SEM; *n* = 4 samples for each condition; *P* values were determined by one-way ANOVA, followed by Dunn (malate and citrate of widetyp (WT) MEFs in **f**; fumarate, malate, α-KG of WT MEFs and succinate of *AMPKα*^–/–^ MEFs in **g**; and malate and citrate of WT MEFs in **h** or Dunnet (others), all compared to the unstarved group. **i**, **j** AMPK promotes the utilization of glutamine during early starvation in mouse muscle. Mice were starved for desired durations, followed by jugular-vein infusion with [U-^13^C]-glutamine or [U-^13^C]-PA tracer, for 2 h, respectively. Mice were then sacrificed, followed by determining the rates of glutaminolysis and FAO as in **a** and **b**. After normalization to the serum levels of corresponding labeled tracers, data were shown as mean ± SEM; *n* = 5 samples for each condition; *P* values were determined by one-way ANOVA, followed by Dunn (fumarate of WT mice, and malate, fumarate, succinate, and PA of *AMPKα*-MKO mice of **j**) or Tukey (others), all compared to the unstarved group. **k** Induction of serum β-hydroxybutyrate, an indicator of hepatic FAO, occurs after prolonged starvation. Mice were starved for desired durations, followed by determining the levels of serum β-hydroxybutyrate (β-HB). Data are shown as mean ± SEM; *n* = 5 mice for each condition; *P* values were determined by one-way ANOVA, followed by Tukey. **l** Muscle-specific knockout of *AMPKα* does not change the levels of serum free fatty acid (NEFA), insulin and glucagon, plasma glucose, or muscle glycogen and triglyceride (TAG). Data are shown as mean ± SEM; *n* = 6 mice for each treatment/genotype; *P* values were determined by two-way ANOVA, followed by Sidak, all compared to the WT group. **m**, **n** AMPK axis promotes OCR during early starvation. WT MEFs and *AMPKα*^–/–^ MEFs (**m**), or WT and *AMPKα*-MKO mice (**n**), were starved for desired durations, followed by determining OCR through Seahorse Analyzer. Data were normalized to the unstarved group of each genotype (same hereafter for all OCR measurements), and are shown as mean ± SEM; *n* values represent biological replicates for each condition, and were labeled in each panel; *P* values were determined by one-way ANOVA, followed by Tukey (left panel, **m**) or by unpaired two-tailed Student’s *t*-test (others). **o**, **p** Inhibition of glutaminolysis, but not FAO, prevents OCR increases. MEFs with *GLS1* knockdown (**o**) or *CPT1* knockout (**p**) were glucose-starved for 2 h (early starvation), followed by determining OCR as in **o**. Data are shown as mean ± SEM; *n* = 6 (**o**) or 5 (**p**) biological replicates for each condition; *P* values were determined by unpaired two-tailed Student’s *t*-test. See also knockout validation data of *CPT1* on the right panel of **p**. Experiments in this figure were performed three times, except experiments in **i** were performed four times.

We first evaluated the dynamics of utilization of alternative carbon sources, i.e., glutamine and fatty acid in low glucose. We pre-labeled mouse embryonic fibroblasts (MEFs) separately with [U-^13^C]-palmitate and [U-^13^C]-glutamine, both to unsaturated states (for respectively determining the rates of FAO and glutaminolysis;^50,51^ see Supplementary information, Note S1 for details), and subjected these cells to glucose starvation. The rates of glutamine utilization, as determined by the levels of ^13^C-labeling of TCA cycle intermediary metabolites (determined by the levels of m + 5 α-ketoglutarate (α-KG); and m + 4 succinate, fumarate, malate and citrate) in MEFs pre-treated with [U-^13^C]-glutamine, were elevated within 2 h of low-glucose treatment (Fig. 1a; Supplementary information, Fig. S1a, b). In comparison, an increase of ^13^C-labeled TCA cycle intermediary metabolites in [U-^13^C]-palmitate pre-treated MEFs (determined by the levels of m + 2 α-KG, succinate, fumarate, malate and citrate) occurred at ∼12 h of starvation, much slower than that with [U-^13^C]-glutamine (Fig. 1b; Supplementary information, Fig. S1c–e; see also Supplementary information, Note S1 for detailed analysis). Therefore, the promotion of glutaminolysis under low glucose conditions occurs ahead of the increase of FAO. We also determined the contributions of these two carbon sources to fuel the TCA cycle in low glucose conditions by labeling MEFs with [U-^13^C]-glutamine and [U-^13^C]-palmitate to a steady, saturated state^50,51^ (see Supplementary information, Note S2 for details). We found that the increased utilization of glutamine, as early as 2 h under the low glucose condition, occurs ahead of the increased utilization of palmitate without changing the total levels of TCA cycle intermediates (Fig. 1c; Supplementary information, Note S2), indicative of a shift of carbon source utilization from glucose to glutamine at the early stage of low glucose treatment. The promotion of glutaminolysis was observed when glucose fell below 5 mM (Fig. 1d; Supplementary information, Fig. S1f), exactly matching the threshold for AMPK activation in MEFs.^37^ Knockout of *AMPKα*, as well as *LKB1*, *AXIN*, or *LAMTOR1* that is required for AMPK activation in low glucose,^38,40^ blocked the promotion of both glutaminolysis within 2-h starvation, and FAO at 12-h starvation in MEFs (Fig. 1e–h; Supplementary information, Fig. S1g–n), leading to deficient energy levels with a drastic accumulation of AMP (Supplementary information, Fig. S1o, see also ref. ^37,52,53^). In contrast, the promotion of glutaminolysis within 2 h of starvation was not affected by MEFs with the knockout of the *RAPTOR* subunit of TORC1 (mTORC1) or *RICTOR* subunit of TORC2 (mTORC2), or inhibition of both mTORC1 and mTORC2 with Torin 1 (ref. ^54^) (Supplementary information, Fig. S2a–c), indicating that AMPK promotes glutaminolysis independently of the regulation of TOR. We also determined the AMPK-dependent promotion of glutaminolysis at the organismal level. Similar to those observed in MEFs, muscular and hepatic glutaminolysis was found to be promoted in mice starved for 8 h (a treatment known to activate AMPK via low glucose/FBP^37^; see also Supplementary information, Fig. S3a for the validation of AMPK activation in tissues), much earlier than FAO (occurring after 16 h of starvation), as measured by infusing mice with U-^13^C-labeled glutamine and palmitate (Fig. 1i, j; Supplementary information, Fig. S3b–e). Consistently, levels of serum β-hydroxybutyrate, an indicator of hepatic FAO,^55,56^ started to increase after 12 h of starvation (Fig. 1k). Knockout of *AMPKα* in skeletal muscle (*AMPKα*-MKO; see validation data in Supplementary information, Fig. S3a; with no change of the levels of blood glucose, insulin, glucagon, free fatty acid or the muscular glycogen and triglyceride (Fig. 1l)) blocked the fasting-induced glutaminolysis in the skeletal muscle of these mice (Fig. 1i, j; Supplementary information, Fig. S3b–e). In line with the results from the isotopic labeling experiments, we observed a rapid increase of oxygen consumption rates (OCR) in both 2 h glucose-starved MEFs and 8 h-starved mouse skeletal muscle tissues, which did not occur in *AMPKα*^–/–^ MEFs (Fig. 1m) or *AMPKα*-MKO mouse skeletal muscles (Fig. 1n). In addition, knockdown of *GLS1* (both *GAC* and *KGA* isoforms), a rate-limiting enzyme of glutaminolysis,^57–59^ or treatment of GLS1 inhibitor BPTES^60^ blocked the increase of OCR (Fig. 1o; Supplementary information, Fig. S3f), while knockout of *CPT1* (both *CPT1α* and *CPT1β*) or treatment of CPT1 inhibitor etomoxir^61^ failed to do so (Fig. 1p; Supplementary information, Fig. S3f). As an additional control, the protein contents of the mitochondrial electron transport chain or the efficiency of electron transfer was unchanged in low glucose (Supplementary information, Fig. S3g), re-assuring that it is the utilization of glutamine that elevates OCR. Together, these results demonstrate that glutamine is a primary carbon source to be catabolized in mitochondria, ahead of fatty acids, thereby compensating for glucose scarcity under starvation.

### PDZD8 is a new substrate for AMPK

**Fig. 2:**
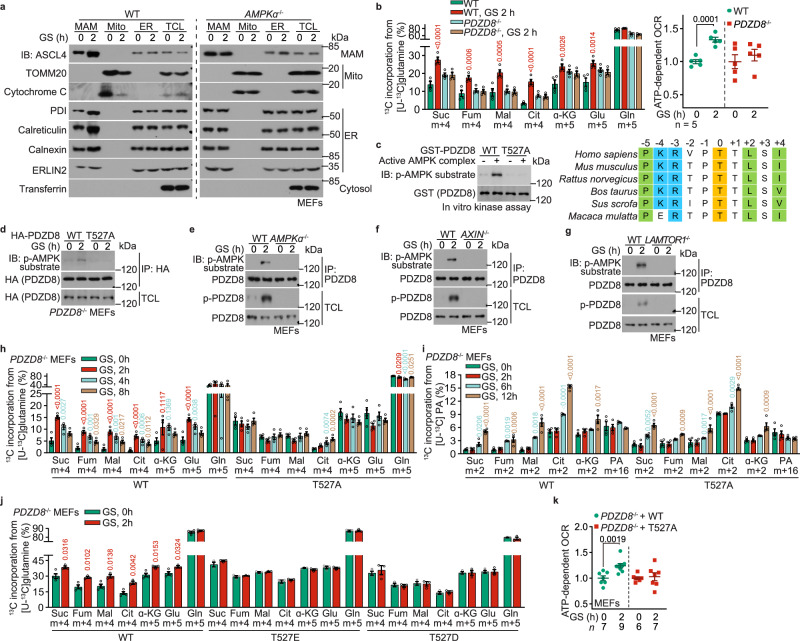
PDZD8 promotes the utilization of glutamine during early starvation. **a** AMPK promotes the association between mitochondria and ER in low glucose. WT MEFs and *AMPKα*^–/–^ MEFs were glucose-starved for 2 h and were subjected to the purification of MAM, mitochondria (mito), and ER. The formation of ER–mitochondria contact was determined either by the protein levels of markers for each subcellular structure via immunoblotting. **b** PDZD8 promotes glutaminolysis during early starvation. WT MEFs and *PDZD8*^–/–^ MEFs were glucose-starved for 2 h, followed by determining the rates of glutaminolysis as in Fig. 1a. Data are shown as mean ± SEM; *n* = 4 biological replicates for each condition; *P* values were determined by two-way ANOVA, followed by Sidak, all compared to the unstarved condition. See also OCR levels, as determined through Seahorse Analyzer, in the right panel, in which data are shown as mean ± SEM; *n* = 5 biological replicates for each condition; *P* values were determined by unpaired two-tailed Student’s *t*-test. **c** AMPK phosphorylates T527 residue of PDZD8 in vitro. 1 μg of GST-tagged recombinant PDZD8 or its T527A mutant was incubated with 0.1 μg of holo-AMPK pre-phosphorylated by CaMKK2, followed by determining the phosphorylation of PDZD8 using immunoblotting (left panel). See also the typical AMPK substrate motif around the phosphoacceptor T527 residue (colored in yellow) of PDZD8, with the basic residues at –4 and –3 positions flanking T527 colored in blue, and the hydrophobic residues at –5, +2, and +4 in green (right panel). **d**–**g** AMPK phosphorylates T527 residue of PDZD8 in cells. MEFs with HA-tagged PDZD8 or PDZD8-T527A stably expressed (**d**), or with knockout of *AMPKα* (**e**), *AXIN* (**f**), or *LAMTOR1* (**g**), were glucose-starved for 2 h, followed by immunoprecipitation of HA-PDZD8 (**d**) or endogenous PDZD8 (**e**–**g**). The immunoprecipitates were then subjected to immunoblotting to determine the levels of p-T527. **h**–**j** AMPK-PDZD8 axis promotes the utilization of glutamine during early starvation. Experiments in **h** and **j** (for determining glutaminolysis) were performed as in Fig. 1e; and experiments in **i** were performed (for determining FAO) as in Fig. 1h; except that *PDZD8*^–/–^ MEFs with WT PDZD8 or PDZD8-T527A re-introduction (**h**, **i**) or PDZD8-T527D/E re-introduction (**j**) were used. Data are shown as mean ± SEM; *n* = 4 (**h**, **i**, and the WT, unstarved group of **j**) or 3 samples (**j**, others) for each condition; *P* values were determined by one-way ANOVA, followed by Dunnet (**h**, **i**), or by unpaired two-tailed Student’s *t*-test (**j**). **k** AMPK-PDZD8 axis promotes OCR during early starvation. WT MEFs and PDZD8-T527A-reintroduced *PDZD8*^–/–^ MEFs were starved for desired durations, followed by determining cellular OCR through the Seahorse analyzer. Data are shown as mean ± SEM; *n* = 4 biological replicates for each condition; *P* values were determined by unpaired two-tailed Student’s *t*-test. Experiments in this figure were performed three times.

We then explored the mechanisms for AMPK to promote the utilization of glutamine in low glucose. We have previously shown that AMPK reduces the yields of pure mitochondria from glucose-starved MEFs after subcellular fractionation,^62^ which turned out to be caused by increased association of mitochondria with ER, or MAM (Fig. 2a). We purified the fractions of MAM and the MAM-tethered mitochondria of glucose-starved MEFs, and then enriched AMPK substrates by using an antibody specifically recognizing pan-phospho-substrates of AMPK that contains the conserved motif to be phosphorylated by AMPK.^63–67^ Through mass spectrometry of the pulldown samples, we identified 12 proteins that were preferentially phosphorylated in glucose-starved cells (listed in Supplementary information, Table S1), among which PDHA1 is a known AMPK substrate.^68^ We next generated expression plasmids for these 12 proteins and found that 3 of them, i.e., PDZD8 and RMDN3, and PDHA1 (as a positive control), were phosphorylated by AMPK in low glucose (Supplementary information, Fig. S4a). Through knocking out these 3 individual genes in MEFs, we found that PDZD8, a known MAM-localized protein required for maintaining ER–mitochondria and ER–lysosome contacts,^69,70^ was required for the promotion of glutaminolysis in the early stage of low glucose treatment (Fig. 2b; Supplementary information, Fig. S4c; see knockout validation data in Supplementary information, Fig. S4b). In comparison, knockout of *RMDN3* or *PDHA1* did not block the low glucose-induced glutaminolysis (Supplementary information, Fig. S4d, e; see knockout validation data in Supplementary information, Fig. S4b). We next determined the phosphorylation site(s) of PDZD8 by AMPK. PDZD8 contains 160 predicted sites (according to ref. ^63–67^; see Supplementary information, Note S3 for the prediction), among which 78 were hit by mass spectrometry (Supplementary information, Table S1). We individually mutated those 78 sites and the other predicted sites as well and found that T527 (for humans; T521 for mice), conserved in mammals (Fig. 2c), is the site of PDZD8 for phosphorylation by AMPK. First of all, p-T527 was hit by the mass spectrometry analysis (see representative spectrogram in Supplementary information, Fig. S4f); secondly, mutation of T527 to alanine (PDZD8-T527A) rendered it unphosphorylable by AMPK in vitro (Fig. 2c); and thirdly, PDZD8-T527A was also unphosphorylable after re-introduction into *PDZD8*^–/–^ MEFs in low glucose (Fig. 2d). We then developed a phospho-specific antibody against p-T527-PDZD8 (see validation data using *PDZD8*^–/–^ MEFs expressing WT PDZD8 or PDZD8-T527A in Supplementary information, Fig. S4g), and found that glucose starvation led to a significant elevation of p-T527 signal in the immunoprecipitants of endogenous PDZD8 (Fig. 2e–g). Moreover, knockout of *AMPKα*, as well as *AXIN* or *LAMTOR1*, which are known components of the glucose-sensing-AMPK axis, abolished the p-T527 signal in low glucose (Fig. 2e–g). These results indicate that PDZD8 is a novel substrate of AMPK that is activated by the lysosomal glucose-sensing pathway. Importantly, the AMPK-PDZD8 axis is specifically involved in the promotion of glutaminolysis, as the re-introduction of PDZD8-T527A into *PDZD8*^–/–^ MEFs only blocked the promotion of glutaminolysis during early starvation, but not the increase of FAO that occurs later on (Fig. 2h, i; Supplementary information, Fig. S4h, i; RMDN3 or PDHA1 also did not affect FAO, see Supplementary information, Fig. S4j–l). In comparison, re-introduction of the phospho-mimetic T527E (T527 to glutamate) mutation of PDZD8 into *PDZD8*^–/–^ MEFs led to a significant increase in glutaminolysis even in high glucose (Fig. 2j; Supplementary information, Fig. S4m; note that the T527D (T527 to aspartate) mutant behaved rather similarly to the T527A mutant). The re-introduction of PDZD8-T527A also blocked the increase of OCR in MEFs in low glucose (Fig. 2k), without disrupting the mitochondrial electron transport chain or the efficiency of electron transfer (Supplementary information, Fig. S3g). Therefore, AMPK phosphorylates PDZD8 at T527 to promote glutamine utilization ahead of the use of fatty acids, to compensate for the scarcity of glucose under starvation.

### PDZD8 promotes GLS1 activity

**Fig. 3:**
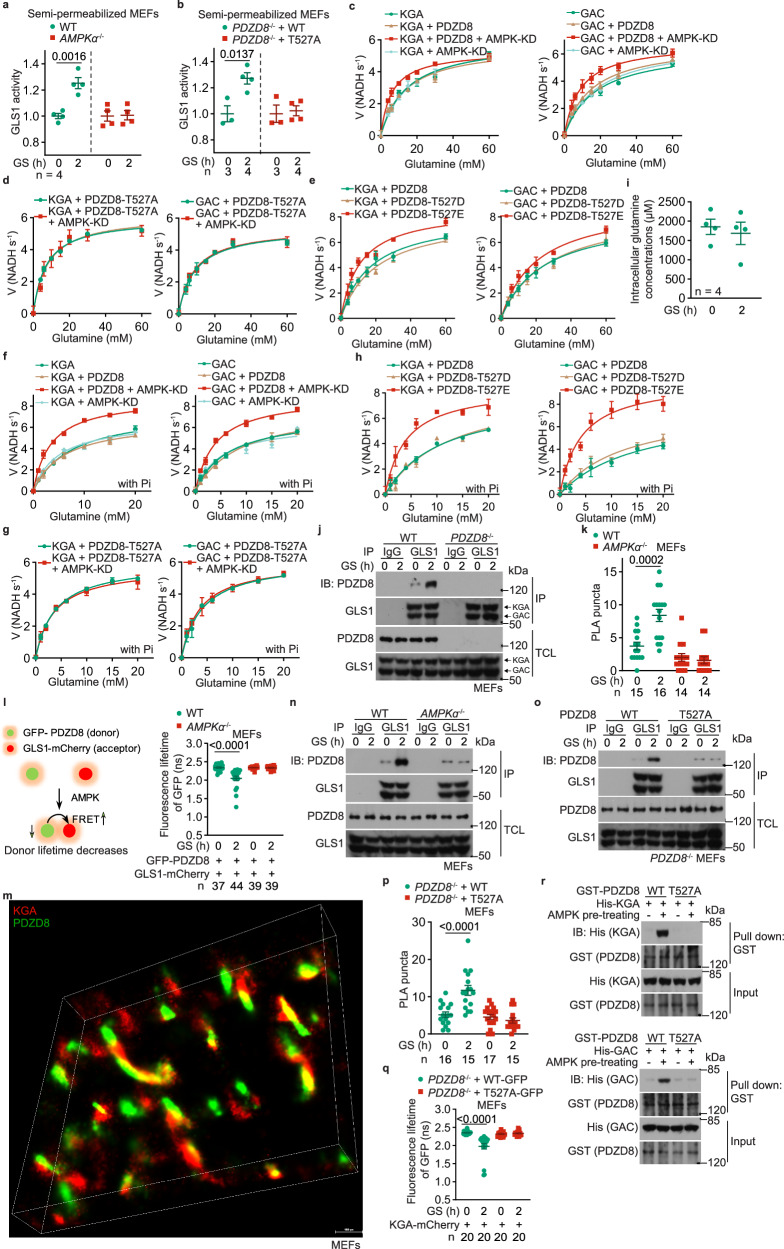
PDZD8 promotes GLS1 activity. **a**, **b** AMPK-PDZD8 axis promotes GLS1 activity in permeabilized cells. WT MEFs, *AMPKα*^–/–^ MEFs (**a**), and WT PDZD8 or PDZD8-T527A-reintroduced *PDZD8*^–/–^ MEFs (**b**) were glucose-starved for 2 h, followed by permeabilization with 0.01% (v/v) NP-40. The activities of GLS1, as evaluated by the production of glutamate after glutamine addition, were then measured. Data are shown as mean ± SD; *n* = 4 (**a**), or labeled on the panel (**b**; representing biological replicates) for each condition; *P* values were determined by Mann–Whitney test (T527A cells of **b**) and by unpaired two-tailed Student’s *t*-test (others). **c**–**h** AMPK-PDZD8 axis promotes GLS1 activity in cell-free systems. Recombinant KGA (left panel) and GAC (right panel) isozymes of GLS1 were mixed with recombinant PDZD8 (**c**, **f**) or PDZD8-T527A (**d**, **g**), or PDZD8-T527D/E (**e**, **h**) protein that was pre-incubated with the constitutively active kinase domain of AMPKα (AMPK-KD; see “Phosphorylation of PDZD8 by AMPK in vitro” in the Materials and Methods section), followed by determination of the enzymatic activities of GLS1. In **f**–**h**, 20 mM K_2_HPO_4_ (Pi) was added to the reactions. Data are shown as mean ± SD; *n* = 3 biological replicates for each condition. See also *K*_m_ and *k*_cat_ values for each reaction in Supplementary information, Table S2. The experiments in **c** and Fig. 4a were performed at the same time and shared control (the KGA- and GAC-alone groups), and ditto for **f** and Fig. 4b. **i** Glucose starvation does not change the intracellular levels of glutamine. Cells were glucose-starved for 2 h, and the intracellular levels of glutamine were determined via high-performance liquid chromatography-mass spectrometry (HPLC-MS). Data are shown as mean ± SEM; *n* = 4 samples for each condition; *P* values were determined by unpaired two-tailed Student’s *t*-test. **j**, **n**, **o** PDZD8 interacts with GLS1, depending on AMPK. WT MEFs and *PDZD8*^–/–^ MEFs (**j**), *AMPKα*^–/–^ MEFs (**n**), and WT PDZD8 or PDZD8-T527A-reintroduced *PDZD8*^–/–^ MEFs (**o**), were glucose-starved for 2 h. Endogenous GLS1 proteins (both KGA and GAC) were immunoprecipitated, followed by immunoblotting to determine co-precipitated PDZD8. **k**, **l**, **p**, **q** AMPK promotes PDZD8–GLS1 interaction in situ. *AMPKα*^–/–^ MEFs (**k**, **l**), or *PDZD8*^–/–^ MEFs (**p**, **q**) were infected with lentiviruses carrying HA-tagged PDZD8 or PDZD8-T527A (**k**, **p**; for PLA), or GLS1 (KGA)-mCherry, along with GFP-PDZD8 (**l**, **q**; for FRET-FLIM assay, see strategy of this assay on the left panel of **l**) or GFP-PDZD8-T527A (**q**). Cells were then glucose-starved for 2 h, followed by quantifying the numbers of PLA puncta in each cell (**k**, **p**; data are shown as mean ± SEM; *n* (labeled on each panel) represents cell numbers for each condition), or measuring the fluorescence lifetime of GFP (the FRET donor; **l**, **q**; data are shown as mean ± SEM; *n* represents cells numbers for each condition); *P* values were determined by two-way ANOVA, followed by Tukey. **m** STORM images showing that PDZD8 is juxtaposed with GLS1 inside cells. MEFs stably expressing FLAG-tagged KGA and Myc-tagged PDZD8 were subjected to STORM imaging, and the representative, reconstituted 3D-STORM image is shown. **r** AMPK promotes PDZD8–GLS1 interaction in vitro. Recombinant His-tagged KGA (upper panel) and GAC (lower panel) isozymes of GLS1 were separately mixed with recombinant GST-tagged PDZD8 or PDZD8-T527A protein that was pre-incubated with AMPK pre-phosphorylated with CaMKK2 (see “Phosphorylation of PDZD8 by AMPK in vitro” in Materials and Methods section), followed by pulling down GST-tag and immunoblotting. Experiments in this figure were performed three times.

We next explored the mechanism through which PDZD8 promotes glutaminolysis. It was found that the activity of GLS1 was significantly promoted in cells starved for glucose, by using a semi-permeabilized assay system (Fig. 3a; validated in Supplementary information, Fig. S5a; see detailed protocol in the Materials and Methods section). Knockout of *AMPKα* blocked the promotion of GLS1 activity (Fig. 3a). We also found that re-introduction of PDZD8-WT, but not PDZD8-T527A, rescued low glucose-induced GLS1 activity in *PDZD8*^–/–^ MEFs (Fig. 3b). These data indicate that the AMPK-PDZD8 axis controls glutaminolysis through regulating GLS1. As a control, we also examined whether glucose starvation causes GLS1 filamentation (supratetrameric oligomerization) that has been shown to enhance the catalytic activity of GLS1 under glutamine starvation,^71^ and found that GLS1 oligomerization was not changed, indicating that GLS1 filamentation did not apply to the regulation by low glucose (Supplementary information, Fig. S5b). We also performed cell-free assays, and found that WT PDZD8, but not the AMPK-unphosphorylable T527A mutant, promoted GLS1 activity in an AMPK-dependent manner (Fig. 3c, d; see *K*_m_ and *k*_cat_ values of each reaction in Supplementary information, Table S2). On the other hand, the phospho-mimetic, PDZD8-T527E mutant increased the activity of GLS1 in vitro even without being phosphorylated by AMPK (Fig. 3e). Free inorganic phosphate in cell-free systems could further activate GLS1 on top of the activation by PDZD8 (Fig. 3f–h), in line with inorganic phosphate being a co-factor of GLS1,^72,73^ indicating that the phosphorylation of PDZD8 and the inorganic phosphate stimulate GLS1 via two independent mechanisms. Data in Fig. 3c–h also revealed that AMPK-phosphorylated PDZD8 increased the affinity of GLS1 towards the substrate glutamine (after phosphorylation by AMPK: *K*_m_ of KGA decreased from 14.63 mM to 6.08 mM in the absence of inorganic phosphate, and from 7.30 mM to 3.46 mM in the presence of inorganic phosphate; and ditto for GAC). Note that the whole-cell concentrations of glutamine were ∼2 mM (see also ref. ^74–76^), and remained similar after low glucose treatment (Fig. 3i). These data indicate that GLS1 is unsaturated with its substrate at all times, consistent with the results that PDZD8 can boost glutamine catabolism to increase glutaminolysis.

**Fig. 4:**
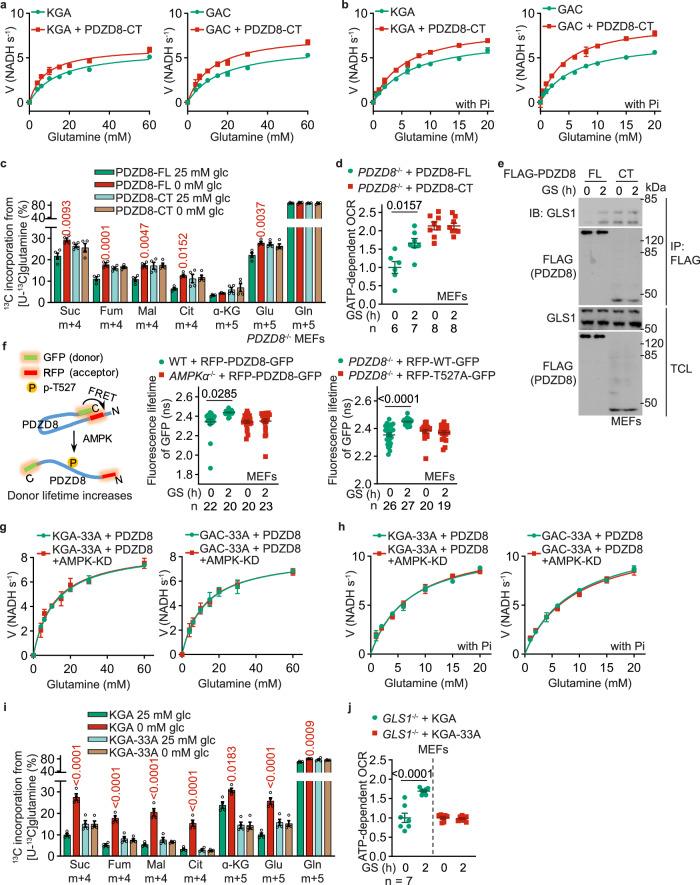
Interaction of PDZD8 promotes GLS1 activity. **a**, **b** PDZD8-CT that constitutively interacts with GLS1, promotes GLS1 activity in vitro independently of AMPK. Recombinant KGA (left panel) or GAC (right panel) isozyme of GLS1 was mixed with recombinant PDZD8-CT, followed by determining the enzymatic activities of GLS1 in the presence (**b**) or absence (**a**) of 20 mM K_2_HPO_4_ (Pi). Data are shown as mean ± SD; *n* = 3 for each condition. See also *K*_m_ and *k*_cat_ values for each reaction in Supplementary information, Table S2. The experiments in (**a**) and Fig. 3c were performed at the same time and shared control (the KGA- and GAC-alone groups), and ditto for **b** and Fig. 3f. **c**, **d** PDZD8-CT promotes glutaminolysis and OCR in high glucose. *PDZD8*^–/–^ MEFs were infected with lentiviruses carrying full-length (FL) PDZD8 or PDZD8-CT, followed by incubating in a medium containing doxycycline for 12 h. Cells were then labeled with [U-^13^C]-glutamine to determine glutaminolysis (**c**, performed as in Fig. 1a) or subjected to Seahorse analyzer to determine OCR (**d**). Data are shown as mean ± SEM; *n* = 4 (**c**), or labeled on the panel (**d**; representing biological replicates) for each condition; *P* values were determined by two-way ANOVA, followed by Tukey (*P* values in **c** represent the comparisons between the starved and the unstarved groups of each genotype). **e** AMPK releases the autoinhibition of PDZD8-NT towards PDZD8-CT. MEFs stably expressing FLAG-tagged PDZD8-FL or PDZD8-CT were glucose-starved for 2 h, followed by immunoprecipitation with anti-FLAG and immunoblotting for co-precipitated GLS1. **f** AMPK causes PDZD8-NT to move away from PDZD8-CT. *AMPKα*^–/–^ MEFs (middle panel), or *PDZD8*^–/–^ MEFs (right panel) were infected with lentiviruses carrying RFP-PDZD8-GFP (middle and right panels) or RFP-PDZD8-T527A-GFP (right panel), followed by determination of the fluorescence lifetime of GFP (FRET donor; see principles of this assay on the left panel). Data are shown as mean ± SEM; *n* values were labeled on the panel representing cell numbers; *P* values were determined by two-way ANOVA, followed by Tukey. **g**–**j** GLS1-33A that loses the interface for PDZD8 fails to promote GLS1 activity (**g**, **h**), glutaminolysis (**i**), or OCR (**j**) in low glucose. Experiments in **g** and **h** were performed as in **a** and **b**, except that the recombinant KGA-33A (left panel) and GAC-33A (right panel) were mixed with AMPK-phosphorylated PDZD8. See also lowered *K*_m_ and increased *k*_cat_ values in each reaction in Supplementary information, Table S2. Experiments in **i** and **j** were performed as in **c** and **d**, except that *GLS1*^–/–^ MEFs with WT KGA or KGA-33A stably expressed were used. Data are mean ± SD; *n* = 3 (**g**, **h**) or 4 (**i**), or labeled on the panel (**j**; representing biological replicates) for each condition; *P* values were determined by two-way ANOVA, followed by Tukey (**i**) or by unpaired two-tailed Student’s *t*-test (**j**). Experiments in this figure were performed three times.

We also tested for possible interaction between PDZD8 and GLS1, and found that they indeed interacted with each other, at endogenous or ectopic levels, and that the interaction became more prominent in cells starved for glucose, as determined by co-immunoprecipitation (Fig. 3j; Supplementary information, Fig. S5c, d). This low glucose-enhanced PDZD8–GLS1 interaction could also be detected in situ, by both the proximity ligation assays (PLAs) in fixed MEFs, and the FRET-FLIM assay in living MEFs (Fig. 3k, l). We also observed that GLS1 is juxtaposed with PDZD8 as determined by both structured illumination microscopy (SIM; Supplementary information, Fig. S5e) and stochastic optical reconstruction microscopy (STORM; Fig. 3m). Knockout of *AMPKα*, or reintroduction of PDZD8-T527A into the *PDZD8*^–/–^ MEFs, abrogated the increase of the interaction between PDZD8 and GLS1 in low glucose (Fig. 3n–q). Consistently, in vitro reconstitution experiments showed that prior phosphorylation with recombinant AMPK increased the affinity of PDZD8, but not PDZD8-T527A towards bacterially purified GLS1 (Fig. 3r). Domain mapping experiments showed that the C-terminus of PDZD8 (PDZD8-CT) constitutes the interface for interaction with GLS1 (Supplementary information, Fig. S5f), as PDZD8-CT alone was sufficient to promote GLS1 activity to the same extent as the full-length PDZD8 pre-treated with AMPK (Fig. 4a, b). Consistently, the re-introduction of PDZD8-CT into *PDZD8*^–/–^ MEFs promoted the utilization of glutamine and OCR, even in high glucose, to similar levels by full-length PDZD8 in low glucose (Fig. 4c, d; Supplementary information, Fig. S5g). These data all suggest that the CT domain acts in a dominant-positive manner for interacting with GLS1. Consistently, we found the N-terminus of PDZD8 (PDZD8-NT) interacts with PDZD8-CT, when expressed separately as truncate proteins in high glucose, and the interaction was abolished in low glucose when AMPK is activated, which indicates that AMPK phosphorylation releases the intramolecular autoinhibition of the N-terminal region towards the C-terminal region of PDZD8 (Fig. 4f; Supplementary information, Fig. S5h). Indeed, AMPK phosphorylation led to an increased affinity of full-length PDZD8 towards GLS1 to an extent similar to that of PDZD8-CT alone towards GLS1 (Fig. 4e; see Supplementary information, Note S4 for details). In addition, we generated a GLS1 mutant (GLS1-33A) carrying mutations to alanine of the 33 amino acid residues on the interface for interacting with PDZD8, which were identified by in silico docking assays (Supplementary information, Fig. S5i). Although GLS1-33A showed similar enzymatic activities to that of the WT GLS1, it was no longer regulated by PDZD8 (Fig. 4g, h). GLS1-33A also blocked the promotion of glutaminolysis or OCR in low glucose (Fig. 4i, j; Supplementary information, Fig. S5j). Results above demonstrate that PDZD8 promotes GLS1 activity through direct interaction in low glucose.

### Physiological functions of the AMPK-PDZD8-GLS1 axis

**Fig. 5:**
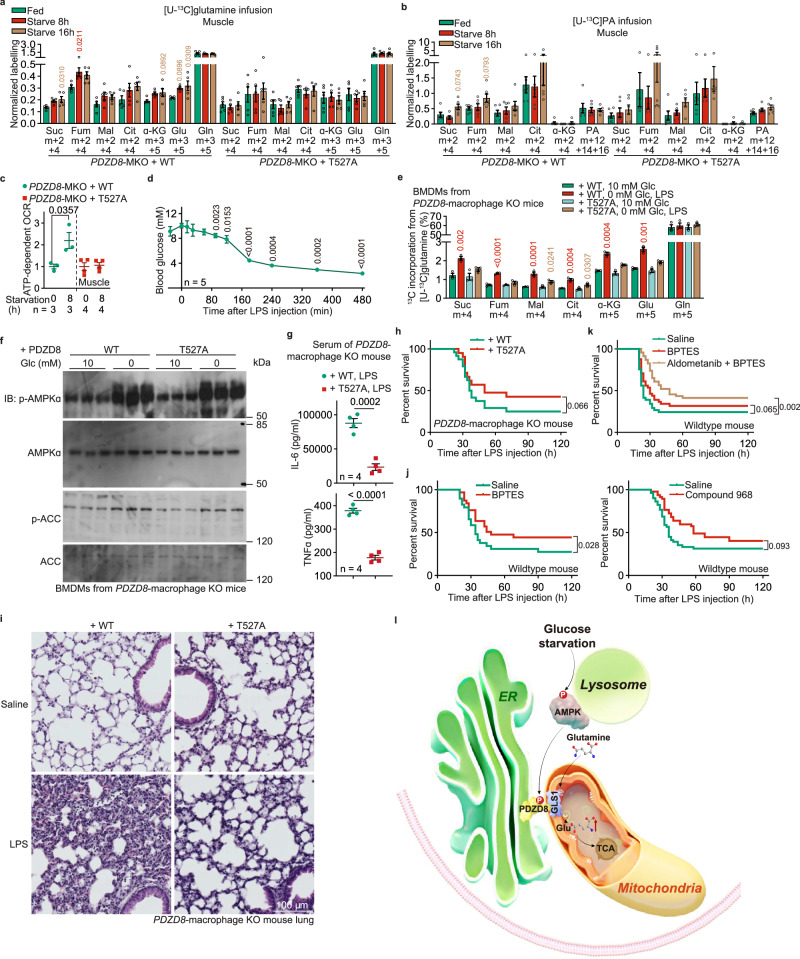
AMPK-PDZD8-GLS1 axis is required for the promotion of glutaminolysis in low glucose in muscle and macrophages. **a**, **b** PDZD8 depends on AMPK to promote the utilization of glutamine in muscle during early starvation. Mice with muscular PDZD8 replaced with WT PDZD8 or PDZD8-T527A were starved for 8 h or 16 h, followed by jugular-vein infusion with [U-^13^C]-glutamine or [U-^13^C]-PA tracer, for 2 h, respectively. Mice were then sacrificed, followed by determining the rates of glutaminolysis and FAO as in Fig. 1a, b, respectively. After normalization to the serum levels of corresponding labeled tracers, data were shown as mean ± SEM; *n* = 5 samples for each condition; *P* values were determined by one-way ANOVA, followed by Dunn (citrate and α-KG of WT, and fumarate, citrate and α-KG of T527A in **b**) or Tukey (others). **c** PDZD8 depends on AMPK to promote muscular OCR during early starvation. Mice with muscular PDZD8 replaced with WT PDZD8 or PDZD8-T527A were starved for 8 h, followed by determining OCR in muscle through Seahorse Analyzer. Data are shown as mean ± SEM; *n* = 3 (muscles from starved WT mice and the PDZD8-WT-reintroduced *PDZD8*-MKO mice), or 4 (others) biological replicates for each condition; *P* values were determined by unpaired two-tailed Student’s *t*-test. **d** Acute LPS treatment causes a decrease in blood glucose. Mice were peritoneally injected with 10 mg/kg LPS, followed by measuring blood glucose at the indicated time points. Results are shown as mean ± SEM; *n* = 5 mice, and *P* values were determined by one-way repeated-measures ANOVA followed by Tukey’s test. **e**, **f** PDZD8 depends on AMPK to promote glutaminolysis in macrophages in low glucose. BMDMs isolated from mice with macrophagic PDZD8 replaced with WT PDZD8 or PDZD8-T527A, were incubated in RPMI 1640 containing 10 mM or 0 glucose and 10 ng/mL LPS for 6 h. Cells were then lysed, followed by determining the rates of glutaminolysis as in Fig. 1a (**e**; cells labeled with [U-^13^C]-glutamine for 1 h before sample collection) and the activation of AMPK (**f**). Results in **e** are shown as mean ± SEM; *n* = 3 mice, and *P* values were determined by two-way ANOVA followed by Sidak. **g**–**i** PDZD8 is required for the pro-inflammatory responses under LPS treatment in an AMPK-dependent manner. Mice with macrophagic PDZD8 replaced with WT PDZD8 or PDZD8-T527A were intraperitoneally injected with 10 mg/kg LPS and were divided into two batches. One batch was used to determine their survival (**h**), which is displayed as Kaplan–Meier curves (see also statistical analyses in Supplementary information, Table S3, and the same hereafter for all lifespan data). The other batch was used to determine the levels of IL-6 and TNF in serum (**g**, collected at 6 h after LPS injection; data shown are shown as mean ± SEM; *n* = 4 mice, and *P* values were determined by unpaired two-tailed Student’s *t*-test) and the damages of lungs (**i**, collected at 24 h after LPS injection). **j**, **k** Inhibition of glutaminolysis blocks the LPS-induced death in mice. WT mice were orally gavaged with 12.5 mg/kg BPTES, intraperitoneally injected with 10 mg/kg compound 968, or orally gavaged with 2 mg/kg aldometanib. After 0.5 h of injection, mice were intraperitoneally injected with 10 mg/kg LPS, followed by determining their survival. Survival curves are displayed as Kaplan–Meier curves. **l** Schematic diagram showing that AMPK-PDZD8 plays a crucial role in the shift of carbon utilization from glucose to glutamine. In low glucose, the ER-localized PDZD8 is phosphorylated at T527 by AMPK activated via the glucose-sensing pathway, which leads to the release of intramolecular autoinhibition (NT towards CT) of PDZD8. As a result, PDZD8 (CT) interacts with and activates the mitochondrial GLS1 and promotes glutaminolysis. Experiments in this figure were performed three times.

We first determined the roles of the enhanced glutaminolysis in maintaining energy homeostasis in low glucose. We found that the re-introduction of PDZD8-T527A mutant into the *PDZD8*^–/–^ MEFs did not cause any elevation of AMP:ATP or ADP:ATP ratio after 2 h of glucose starvation (Supplementary information, Fig. S6a), a duration in which FAO was not yet promoted by AMPK (Fig. 2i). We then generated mice with muscular PDZD8 replaced with PDZD8-T527A (by skeletal muscle-specific knockout of *PDZD8* and inducing expression of PDZD8-T527A knocked-in into skeletal muscle; validated in Supplementary information, Fig. S6b), and confirmed that the fasting-induced glutaminolysis, as well as OCR, was blocked in the skeletal muscle of these mice as did in MEFs (Fig. 5a–c; Supplementary information, Fig. S6c–f). Similar to MEFs, we did not observe any elevation of AMP or ADP, or impaired mitochondrial electron transport chain, in the skeletal muscle of these mice after 8 h of fasting (Supplementary information, Fig. S6g). These findings are in sharp contrast to the results observed in the knockout of *AMPKα*, *AXIN*, or *LAMTOR1* (Supplementary information, Fig. S1o), suggesting that other nutrients can supplement the deprivation of glucose and that AMPK can maintain energy homeostasis through various mechanisms other than glutaminolysis, such as by inhibiting other pathways that consume ATP like protein and lipid synthesis.^77^

It is also known that glutaminolysis is critical for the secretion of cytokines following treatment of LPS,^78^ which leads to a rapid decline of blood glucose.^79^ As another readout of the physiological function of enhancement of glutaminolysis by the AMPK-PDZD8-GLS1 axis, we also tested the secretion of pro-inflammatory cytokines in macrophages. We first titrated the effects of different LPS doses on blood glucose levels and found that intraperitoneal injection of LPS at 10 mg/kg, which resulted in over 60% death of mice within 48 h, caused a decrease in blood glucose to 5 mM or below — the threshold for activating AMPK^37^ — within 3 h of administration (Fig. 5d, h). We then generated mice with macrophagic PDZD8 replaced with PDZD8-T527A (by specifically knocking out macrophagic *PDZD8* and inducing expression of PDZD8-T527A knocked-in into macrophages; validated in Supplementary information, Fig. S7a), and confirmed that the bone marrow-derived macrophages (BMDMs) of these mice showed an impaired glutaminolysis when stimulated with LPS, in which glucose was low and AMPK was activated (Fig. 5e, f, Supplementary information, Fig. S7b). When these mice were treated with 10 mg/kg LPS, we observed that PDZD8-T527A reduced the levels of pro-inflammatory cytokines, such as TNFα and IL-6, in their serum (Fig. 5g). This observation indicates that the AMPK-unphosphoryable PDZD8 mutant can retard cytokine secretion. As a result, the death rates of these mice were significantly decreased (with the median survival time improved by ∼40%; Fig. 5h; see also statistical analyses on Supplementary information, Table S3), accompanied with improved lung damage (Fig. 5i). The PDZD8-T527A-suppressed inflammatory effects could also be observed in vitro, as the PDZD8-T527A-reintroduced *PDZD8*^–/–^ BMDM showed a much lower ability to secrete pro-inflammatory cytokines when stimulated with LPS in low glucose (Supplementary information, Fig. S7c). The results above indicate that the AMPK-PDZD8-GLS1-enhanced glutaminolysis is important in eliciting pro-inflammatory responses under LPS administration. Consistently, when we treated mice with BPTES, at a dose of 12.5 mg/kg, which has been proven effective in inhibiting GLS1 in vivo,^80^ after administering LPS, we observed an ∼40% reduction in mouse death (Fig. 5j). Similar results were obtained in mice treated with 10 mg/kg compound 968 (ref. ^81^), another GLS1 inhibitor^82^ (Fig. 5j). Finally, as AMPK per se is a potent inhibitor of pro-inflammatory responses,^83^ we proposed that activation of AMPK prior to the LPS treatment could enhance the protective effects of GLS1 inhibitors against LPS-induced death in mice. Indeed, we found that mice treated with aldometanib that mimics low glucose-induced AMPK activation,^84^ in combination with BPTES, rescued death by ∼70% (Fig. 5k).

## Discussion

In this study, we have investigated the metabolic changes and the underlying mechanisms that entail AMPK activation by fasting or lowered glucose, and identified PDZD8 as a new substrate for AMPK. PDZD8 phosphorylated by AMPK releases its intramolecular inhibition, allowing its CT to interact with and promote the activity of GLS1 under physiological concentrations of glutamine (Fig. 5l). As a result, the enzymatic activity of GLS1 is increased, leading to increased glutaminolysis in MEFs, and in mouse macrophages and the skeletal muscle. Through isotopic tracing experiments, we have shown that the increase of glutaminolysis occurs prior to that of FAO, in line with glutamine being the most abundant circulating amino acid and rapidly replenished by other amino acids such as BCAA mobilized from labile proteins in muscle tissues during starvation.^18^ Glutamine offers several advantages over fatty acids. First of all, glutamine is an abundant amino acid, circulating at ∼500 μM in the serum, and is at even higher concentrations in the interstitial space of muscle during fasting.^6,85^ In contrast, the circulating and muscle-interstitial free fatty acid is ∼20-fold lower,^86^ perhaps as a way to prevent cells from lipotoxicity; free fatty acids are strictly constrained inside cells or tissues, as two-thirds of fatty acid mobilized from adipose tissues after starvation is re-esterified into triglyceride (futile cycle), while the remaining one-third is burned by muscles.^8,87^ In addition, the rates of glutamine oxidation, at least in the skeletal muscle and MEFs, are much faster than that of fatty acids. Glutamine, when administered as a labeled tracer, enters the TCA cycle of skeletal muscle and MEFs at a much faster rate than that of palmitic acid (ref. ^6^, also this study, see Supplementary information, Fig. S7d). Consistently, as also shown in this study, GLS1 activity is directly boosted upon lysosomal AMPK activation, as a rapid response to glucose/FBP decreases, during which no promotion of FAO was observed. Supporting this notion, we also observed that malonyl-CoA in MEFs that inhibits FAO, started to decrease only after 8 h of glucose starvation (Supplementary information, Fig. S7e), possibly owing to a lack of ACC2 phosphorylation when only the lysosomal pool of AMPK is activated under early starvation condition.^62^ In comparison, when a stronger activation of AMPK was achieved by the allosteric pan-activator MK-8722 (ref. ^88^), both FAO and glutaminolysis were promoted within 2 h of treatment, in an AMPK-dependent manner (Supplementary information, Fig. S7f, g). It should be noted that the mitochondrial inhibitors such as rotenone and oligomycin A, despite activating AMPK more robustly than glucose starvation through elevating AMP levels^62,89^ can instead inhibit glutaminolysis and FAO by blocking the TCA cycle (Supplementary information, Fig. S7h–j).

It is intriguing that PDZD8, as an ER/MAM-localized protein, interacts with mitochondrial GLS1 upon phosphorylation by AMPK. Given that PDZD8 is shown to be required for maintaining the ER–mitochondria contacts by acting as a member of the mammalian ER–mitochondria encounter structure (ERMES) complex,^70^ it is reasonable to speculate that the interaction between PDZD8 and GLS1 occurs at the contact site between ER and mitochondria. In mitochondria, GLS1 has been reported to be localized on both the external^90–92^ and internal sides of the inner mitochondrial membrane (IMM)^93,94^ and the mitochondrial matrix.^92,95,96^ It is, therefore, interesting to determine which pool of GLS1 can interact with PDZD8 in low glucose seen during fasting. To that end, we performed the APEX2 (ascorbate peroxidase 2) proximity labeling experiments^97^ using MEFs stably expressing a chimera of the biotinylating enzyme APEX2 fused to the CT of PDZD8 under the control of a doxycycline-inducible promoter, and found a significant enrichment of biotinylated GLS1 in purified IMM from starved cells, while GLS1 was hardly biotinylated in the purified mitochondria matrix regardless of starvation (Supplementary information, Fig. S7k). If PDZD8-APEX2 interacted with GLS1 localized on the internal side of IMM, the matrix GLS1 may probably be biotinylated, but this did not happen. Therefore, PDZD8 may interact with GLS1 on the external side of IMM. Consistently, the IMM pool of GLS1 is known to be enzymatically active, as the GLS1 localized in the internal sides of IMM and matrix is constantly inhibited by the high concentrations of glutamate in the matrix.^90,92,98–100^ Although how PDZD8 flips to the outer face of IMM is still unknown, proteins from ERMES, and those localized in the outer mitochondrial membrane (OMM), intermembrane space, and IMM might be required. In yeast, it has been shown that the ERMES integral member Mdm10 is also a component of the protein sorting and assembly machinery (SAM) complex on the OMM.^101,102^ In mammals, the IMM-localized ATAD3A was shown to interact with OMM and ER-resident proteins through chaperons localized in the ER–mitochondria contact sites.^103,104^ We also found that the chaperone protein 14-3-3 that facilitates the import of protein precursors into mitochondria,^105^ interacted with PDZD8 (Supplementary information, Fig. S7l). Despite the interaction, 14-3-3 does not play a role in the interaction of PDZD8 and GLS1, as the PDZD8–GLS1 interaction could be observed by using the bacterially expressed proteins (Fig. 3r). How these proteins/protein complexes facilitate the apposition between PDZD8 and GLS1 inside the cells will be of interest and awaits further exploration. In addition to the ER–mitochondria contact, PDZD8 is also shown to be localized on the ER–lysosome contacts,^70^ and this might explain how it is phosphorylated by the lysosomal pool of AMPK in low glucose.

Along with the role in the promotion of glutaminolysis, the AMPK-PDZD8-GLS1 axis is demonstrated here also to play an important role in the promotion of the secretion of pro-inflammatory cytokines by macrophages of mice exposed to lethal doses of LPS that causes the decline of blood glucose levels. Inhibition of the AMPK-PDZD8-GLS1 axis effectively suppresses the cytokine storm and prevents the death of the LPS-administered mice. However, there have been reports that show protective roles of the macrophage-secreted cytokines in preventing pathogen invasion and proliferation, as seen during the infection of lymphocytic choriomeningitis virus or influenza A virus (IAV)^106–108^ that also causes the decline of blood glucose.^109,110^ It would also be interesting to explore other benefits of the AMPK-PDZD8-GLS1 axis in mediating the extension of lifespan and healthspan by calorie restriction. Together, our study reveals an AMPK-PDZD8-GLS1 axis that transmits low glucose-activated AMPK activity to phosphorylation of PDZD8, and to enhancement of glutaminolysis via increased activity of GLS1. This axis not only compensates for the reduction of glucose usage, but also elicits other biological functions such as the promotion of the secretion of immunocytokines.

## Materials and methods

### Antibodies

Rabbit anti-phospho-AMPKα-Thr172 (Cat# 2535, RRID: AB_331250; 1:1000 for immunoblotting (IB)), anti-AMPKα (Cat# 2532, RRID: AB_330331; 1:1000 for IB), anti-phospho-AMPK substrate motif (Cat# 5759, RRID: AB_10949320; 1:1000 for IB and 1:25 for immunoprecipitation (IP)), anti-phospho-ACC-Ser79 (Cat# 3661, RRID: AB_330337; 1:1000 for IB), anti-ACC (Cat# 3662, RRID: AB_2219400; 1:1000 for IB), anti-cytochrome C (Cat# 4280, RRID: AB_10695410; 1:500 for IB), anti-PDI (Cat# 3501, RRID: AB_2156433; 1:1000 for IB), anti-calreticulin (Cat# 12238, RRID: AB_2688013; 1:1000 for IB), anti-erlin2 (Cat# 2959, RRID: AB_2277907; 1:1000 for IB), anti-PDH (Cat# 3205, RRID: AB_2277907; 1:1000 for IB), anti-COXIV (Cat# 4850, RRID: AB_2085424; 1:1000 for IB); anti-14-3-3 (Cat# 95422; 1:1000 for IB), anti-GST-tag (Cat# 2625, RRID: AB_490796; 1:4000 for IB), anti-His-tag (Cat# 12698, RRID: AB_2744546; 1:1000 for IB), anti-Myc-tag (Cat# 2278, RRID: AB_490778; 1:120 for immunofluorescence (IF)), horseradish peroxidase (HRP)-conjugated mouse anti-rabbit IgG (conformation-specific, Cat# 5127, RRID: AB_10892860; 1:2000 for IB), HRP-conjugated goat anti-rat IgG (conformation-specific, Cat# 98164; 1:2000 for IB) and mouse anti-Myc-tag (Cat# 2276, RRID: AB_331783; 1:500 for IB) antibodies were purchased from Cell Signaling Technology. Rabbit anti-calnexin (Cat# ab22595, RRID: AB_2069006; 1:1000 for IB), anti-transferrin (Cat# ab1223, RRID: AB_298951; 1:500 for IB), anti-GLS1 (Cat# ab202027; 1:120 for IF), and mouse anti-CPT1α (Cat# ab128568, RRID: AB_11141632; 1:1000 for IB), mouse anti-total oxidative phosphorylation (OXPHOS) complex (Cat# ab110413, RRID: AB_2629281; 1:1000 for IB) antibodies were purchased from Abcam. Rabbit anti-PDZD8 (Cat# NBP2-58671; 1:1000 for IB or 1:100 for IP; validated in Supplementary information, Fig. S4b) was purchased from Novus Biologicals. Mouse anti-ASCL4 (also known as FACL4; Cat# sc-365230, RRID: AB_10843105; 1:1000 for IB) and anti-HA-tag (Cat# sc-7392, RRID: AB_2894930; 1:1000 for IB, 1:500 for IP or 1:120 for IF) antibodies were purchased from Santa Cruz Biotechnology. Rabbit anti-GLS1 (KGA and GAC; Cat# 12855-1-AP, RRID: AB_2110381; 1:2000 for IB and 1:100 for IP), anti-TOMM20 (Cat# 11802-1-AP, RRID: AB_2207530; 1:1000 for IB), anti-PDK4 (Cat# 12949-1-AP, RRID: AB_2161499; 1:1000 for IB), anti-CPT1β (Cat# 22170-1-AP, RRID: AB_2713959; 1:1000 for IB), anti-PDH E1 alpha (PDHA1; Cat# 18068-1-AP, RRID: AB_2162931; 1:5000 for IB), and mouse anti-tubulin (Cat# 66031-1-Ig, RRID: AB_11042766; 1:20,000 for IB mammalian tubulin) antibodies were purchased from Proteintech. Rabbit anti-APEX2 (Cat# PA5-72607; 1:1000 for IB) antibody was purchased from ThermoFisher Scientific. Mouse anti-FLAG M2 (Cat# F1804, RRID: AB_262044; 1:1000 for IB) antibody was purchased from Sigma. Rabbit anti-RMDN3 (also known as PTPIP51; Cat# A5820, RRID: AB_2766572; 1:1000 for IB) antibody was purchased from Abclonal. The HRP-conjugated goat anti-mouse IgG (Cat# 115-035-003, RRID: AB_10015289; 1:5000 dilution for IB) and goat anti-rabbit IgG (Cat# 111-035-003, RRID: AB_2313567; 1:5000 dilution for IB and 1:120 dilution for IHC) antibodies were purchased from Jackson ImmunoResearch.

### Chemicals and assay kits

Aldometanib was synthesized as described previously^84^ and is now available at MedChemExpress (Cat# HY-148189), GLPBIO (Cat# GC66024), and CymitQuimica (Cat# TM-T60122). Glucose (Cat# G7021), DMSO (Cat# D2650), PBS (Cat# P5493), NaCl (Cat# S7653), KCl (Cat# P9333), HCl (Cat# 320331), ATP (disodium salt; Cat# A6419), ATP (magnesium salt, for kinase assay; Cat# A9187), SDS (Cat# 436143), CaCl_2_ (Cat# C5670), KH_2_PO_4_ (Cat# P5655), K_2_HPO_4_ (Cat# P9666), HEPES (Cat# H4034), MES (Cat# 69889), EDTA (Cat# E6758), EGTA (Cat# E3889), MgCl_2_ (Cat# M8266), CsCl (Cat# 289329), NaAc (Cat# S7670), ethanol (Cat# 459836), glycerol (Cat# G5516), IGEPAL CA-630 (NP-40, Cat# I3021), Triton X-100 (Cat# T9284), Tween-20 (Cat# P9416), cholesteryl hemisuccinate (CHS; Cat# C6512), sodium deoxycholate (Cat# S1827), dithiothreitol (DTT; Cat# 43815), IPTG (Cat# I6758), nuclease-free water (for IVF; Cat# W4502), L-glutathione reduced (GSH; Cat# G4251), mineral oil (Cat# M5310 for IVF, and Cat# M5904 for CsCl density gradient), biotinyl tyramide (biotin-phenol; Cat# SML2135), Trizma base (Tris; Cat# T1503), hexadimethrine bromide (polybrene; Cat# H9268), sodium pyrophosphate (Cat# P8135), β-glycerophosphate (Cat# 50020), hydrogen peroxide (H_2_O_2_; Cat# H1009), sodium azide (NaN_3_; Cat# S2002), sodium ascorbate (Cat# A4034), 6-Hydroxy-2,5,7,8-tetramethylchromane-2-carboxylic acid (Trolox; Cat# 238813), sodium carbonate (Na_2_CO_3_; Cat# S7795), *O*-(carboxymethyl)hydroxylamine hemihydrochloride (AOA; Cat# C13408), urea (Cat# U5378), myristic-d27 acid (Cat# 68698), glutamine (Cat# G8540), carnitine (Cat# C0283), BSA (Cat# A2153), fatty acid-free BSA (Cat# SRE0098), methoxyamine hydrochloride (Cat# 89803), MTBSTFA (with 1% t-BDMCS; Cat# M-108), pyridine (Cat# 270970), sodium palmitate (PA; Cat# P9767), methanol (Cat# 646377), chloroform (Cat# C7559), heparin sodium salt (Cat# H3149), acetonitrile (Cat# 34888), ammonium acetate (Cat# 73594), ammonium hydroxide solution (Cat# 338818), LC-MS-grade water (Cat# 1153332500), mannitol (Cat# M4125), L-methionine sulfone (Cat# M0876), D-campher-10-sulfonic acid (Cat# 1087520), 3-aminopyrrolidine dihydrochloride (Cat# 404624), *N,N*-diethyl-2-phenylacetamide (Cat# 384011), trimesic acid (Cat# 482749), diammonium hydrogen phosphate (Cat# 1012070500), ammonium trifluoroacetate (Cat# 56865), paraformaldehyde (Cat# 158127), haematoxylin solution (Cat# 03971), eosin Y solution (Cat# 318906), Canada balsam (Cat# C1795), xylene (Cat# 214736), oligomycin A (Cat# 75351), FCCP (Cat# C2920), antimycin A (Cat# A8674), rotenone (Cat# R8875), gentamycin (Cat# 345814), collagenase A (Cat# 11088793001), imidazole (Cat# I5513), taurine (Cat# T8691), ADP (Cat# 01897), phosphocreatine (Cat# V900832), leupeptin (L2884), saponin (Cat# S4521), lactobionate (Cat# L3375), glutamate (Cat# G8415), malate (Cat# M7397), succinate (Cat# S9512), sucrose (Cat# S7903), digitonin (Cat# D141), sodium pyruvate (for Oxygraph-2k measurement; Cat# P5280), formaldehyde solution (formalin; F8775), glutaraldehyde solution (Cat# G5882), glycine (Cat# G8898), K_3_Fe(CN)_6_ (Cat# 455946), thiocarbonohydrazide (Cat# 223220), Pb(NO_3_)_2_ (Cat# 203580), sodium citrate (Cat# 71497), potassium acetate (Cat# P1190), magnesium acetate (Cat# M5661), MEA (Cat# 30070), glucose oxidase (Cat# G2133), catalase (Cat# C40), OptiPrep (Cat# D1556), Percoll (Cat# P4937), Coomassie Brilliant Blue R-250 (Cat# 1.12553), chymotrypsin (Cat# C3142), formic acid (Cat# 5.43804), β-mercaptoethanol (Cat# M6250), MOPS (Cat# M3183), acetic acid (Cat# 27225), L-glutamic dehydrogenase (GDH; Cat# G2626), NAD^+^ (Cat# N3014), BPTES (Cat# SML0601), LPS (Cat# L2630), etomoxir (Cat# 236020), human tubal fluid (HTF) medium (Cat# MR-070-D), KSOM medium (Cat# MR-121-D), triple-free DMEM (Cat# D5030), Lysosome Isolation Kit (Cat# LYSISO1), Endoplasmic Reticulum Isolation Kit (Cat# ER0100), Glutamate Assay Kit (Cat# MAK004), anti-FLAG M2 affinity gel (Cat# A2220; 1:500 for IP), FLAG peptide (Cat# F3290), HIS-Select Nickel Affinity Gel (Cat# P6611), and Duolink In Situ Red Starter Kit (Mouse/Rabbit; Cat# DUO92101) were purchased from Sigma. MK-8722 (Cat# HY-111363), R162 (Cat# HY-103096), and compound 968 (Cat# HY-12682) were purchased from MedChemExpress. Torin 1 (Cat# S2827) was purchased from Selleck. Penicillin-streptomycin (Cat# 15140163), Phusion High-Fidelity DNA Polymerase kit (Cat# F530N), mMESSAGE mMACHINE T7 Transcription Kit (Cat# AM1344), MEGAclear Transcription Clean-Up Kit (Cat# AM1908), MEGAshortscript T7 Transcription Kit (Cat# AM1354), SulfoLink Immobilization Kit for Peptides (Cat# 44999), IL-6 Mouse Uncoated ELISA Kit (Cat# 88-7064-88), TNF alpha Mouse Uncoated ELISA Kit (Cat# 88-7324-88), DMEM, high glucose (DMEM; Cat# 11965175), glucose-free DMEM (Cat# 11966025), RPMI 1640 medium (Cat# 11875119), FBS (Cat# 10099141 C), Lipofectamine 2000 (Cat# 11668500), MEM non-essential amino acids solution (Cat# 11140050), GlutaMAX (Cat# 35050061), sodium pyruvate (Cat# 11360070), ProLong Diamond antifade mountant (Cat# P36970), ProLong Live Antifade reagent (Cat# P36975), Streptavidin Magnetic Beads (Cat# 88817; 1:100 for IP), NeutrAvidin agarose (Cat# 29204), EZ-Link Sulfo-NHS-SS-Biotin (Cat# 21331), and Prestained Protein MW Marker (Cat# 26612) were purchased from ThermoFisher Scientific. Red Cell Lysis Buffer (Cat# RT122) was purchased from TIANGEN. OsO_4_ (Cat# 18465) and uranyl acetate (Cat# 19481) were purchased from Tedpella. Paraplast (Cat# 39602012) was purchased from Leica. SPI-Pon 812 Embedding Kit (Cat# 02660-AB) was purchased from Structure Probe, Inc. *n*-dodecyl-β-D-maltopyranoside (DDM; Cat# D310) was purchased from Anatrace Products, LLC. Difco LB Broth (Cat# 240220) was purchased from BD. Seahorse XF base medium (Cat# 103334) and Seahorse XF Calibrant solution (Cat# 100840) were purchased from Agilent. Antifade Mounting Medium (Cat# H-1000-10) was purchased from Vector Laboratories, Inc. PrimeSTAR HS polymerase (Cat# R40A) was purchased from Takara. Polyethylenimine (PEI; Cat# 23966) was purchased from Polysciences. Nonfat dry milk (Cat# #9999) and normal goat serum (NGS; Cat# #5425) were purchased from Cell Signaling Technology. Protease inhibitor cocktail (Cat# 70221) was purchased from Roche. WesternBright ECL and peroxide solutions (Cat# 210414-73) were purchased from Advansta. [U-^13^C]-glutamine (Cat# 184161-19-1), [U-^13^C]-palmitate ([U-^13^C]-PA; Cat# CLM-3943), [alpha-^15^N]-glutamine (Cat# NLM-1016), tryptophan-d5 (Cat# DLM-1092), and [U-^13^C]-glucose (CLM-1396) were purchased from Cambridge Isotope Laboratories. The isotope-labeled AMP (Cat# 123603801), ADP (Cat# 129603601), and ATP (Cat# 121603801) standards were purchased from Silantes. 3-hydroxynaphthalene-2,7-disulfonic acid disodium salt (2-naphtol-3,6-disulfonic acid disodium salt; Cat# H949580) was purchased from Toronto Research Chemicals. Hexakis(1H,1H,3H-perfluoropropoxy)phosphazene (hexakis(1H,1H, 3H-tetrafluoropropoxy)phosphazine; Cat# sc-263379) was purchased from Santa Cruz Biotechnology. MinElute PCR Purification Kit (Cat# 28004) was purchased from Qiagen. Human chorionic gonadotrophin (hCG) and pregnant mare’s serum gonadotrophin (PMSG) were purchased from Sansheng Biological Technology Co., Ltd. (Ningbo, China). rProtein A Sepharose Fast Flow (Cat# 17127904), Protein G Sepharose 4 Fast Flow (Cat# 17061806), Glutathione Sepharose 4 Fast Flow (Cat# 17513203), and Superdex 200 Increase 10/300 GL (Cat# 28990944) were purchased from Cytiva.

### Mouse strains

Protocols for all rodent experiments were approved by the Institutional Animal Care and the Animal Committee of Xiamen University (XMULAC20180028 and XMULAC20220050). WT C57BL/6 J mice (#000664) were obtained from The Jackson Laboratory. *AXIN*^F/F^ and *LAMTOR1*^F/F^ mice were generated and validated as described previously.^38^ *AMPKα1*^F/F^ (Cat# 014141), *AMPKα2* ^F/F^ (Cat# 014142), and *RICTOR*^F/F^ (Cat# 020649) mice were obtained from The Jackson Laboratory, provided by Dr. Sean Morrison. *LKB1*^F/F^ mice were obtained from Frederick National Laboratory for Cancer Research and provided by Dr. Ronald A. DePinho. *PDZD8*^–/–^ (KO-first; *Pdzd8*^*tm1a(EUCOMM)Wtsi*^) mice were obtained from Wellcome Trust Sanger Institute, and *GLS1*^F/F^ mice (#T015195) from GemPharmatech. *AMPKα1*/*2*^F/F^ mice were crossed with *Mck*-*Cre* mice to generate skeletal muscle-specific knockout (*AMPKα*-MKO) mice (validated in ref. ^84^).

To generate mice with muscular PDZD8 replaced with WT PDZD8 or PDZD8-T527A, the *PDZD8*^–/–^ mice were first crossed with FLPo mice (036512-UCD; MMRRC) to generate the *PDZD8*^F/F^ mice. WT PDZD8 or its T527A mutant was then introduced to the *PDZD8*^F/F^ mice under the *Rosa26*-LSL(*LoxP*-*Stop*-*LoxP*) system,^111^ followed by crossing with *HSA-CreERT2* mice (Cat# 025750; The Jackson Laboratory). The removal of endogenous PDZD8 and the LSL cassette ahead of introduced PDZD8 and PDZD8-T527A (to trigger the expression of introduced PDZD8) was achieved by intraperitoneally injecting mice with tamoxifen (dissolved in corn oil) at 200 mg/kg, 3 times a week. To generate mice with macrophagic PDZD8 being replaced with PDZD8 or PDZD8-T527A, the *PDZD8*^F/F^ mice carrying PDZD8 or PDZD8-T527A under the *Rosa26*-LSL(*LoxP*-*Stop*-*LoxP*) cassette were crossed with *VAV1*-*Cre* (Cat# 008610; The Jackson Laboratory) mice.

To introduce PDZD8 or PDZD8-T527A into *PDZD8*^F/F^ mice, cDNA fragments encoding PDZD8 or PDZD8-T527A were inserted into the *Rosa26*-CTV vector, followed by purification of the plasmids using CsCl density gradient ultracentrifugation method. Some 100 μg of plasmid was then diluted with 500 μL of di-distilled water, followed by concentrating via centrifuge at 14,000× *g* at room temperature in a 30-kDa-cutoff filter (UFC503096, Millipore) to 50 μL of solution. The solution was diluted with 450 μL of di-distilled water, followed by another two rounds of dilution/concentration cycles. The plasmid was then mixed with 50 μL of di-distilled water to a final volume of 100 μL, followed by mixing with 10 μL of NaAc solution (3 M stock concentration, pH 5.2). The mixture was then mixed with 275 μL of ethanol, followed by incubating at room temperature for 30 min to precipitate plasmid. The precipitated plasmid was collected by centrifuge at 16,000× *g* for 10 min at room temperature, followed by washing with 800 μL of 75% (v/v) ethanol (in di-distilled water) twice. After evaporating ethanol by placing the plasmid next to an alcohol burner lamp for 10 min, the plasmid was dissolved in 100 μL of nuclease-free water. The plasmid, along with *SpCas9* mRNA and the sgRNAs against the mouse *Rosa26* locus, were then microinjected into the in vitro fertilized (IVF) embryos of the *PDZD8*^F/F^ mice. To generate the *SpCas9* mRNA, 1 ng of pcDNA3.3-hCas9 plasmid (constructed by inserting the Cas9 fragment released from Addgene Cat# 41815 into the pcDNA3.3 vector; diluted to 1 ng/μL) was amplified using the Phusion High-Fidelity DNA Polymerase kit on a thermocycler (Cat# T100, Bio-Rad) with the following programs: pre-denaturing at 98 °C for 30 s; denaturing at 98 °C for 10 s, annealing at 68 °C for 25 s, then extending at 72 °C for 2 min in each cycle; and final extending at 72 °C for 2 min; cycle number: 33. The following primer pairs were used: 5′-CACCGACTGAGCTCCTTAAG-3′, and 5′-TAGTCAAGCTTCCATGGCTCGA-3′. The PCR product was then purified using the MinElute PCR Purification Kit following the manufacturer’s instructions. The purified SpCas9 PCR product was then subjected to in vitro transcription using the mMESSAGE mMACHINE T7 Transcription Kit following the manufacturer’s instruction (with minor modifications). Briefly, 5.5 μL (300 ng/μL) of SpCas9 PCR product as the template was mixed with 10 μL of 2× NTP/ARCA solution, 2 μL of 10× T7 Reaction Buffer, 0.5 μL of RNase inhibitor, 2 μL of T7 Enzyme Mix, and 4.5 μL of nuclease-free water, followed by incubating at 37 °C for 2 h. The mixture was then mixed with 1 μL of Turbo DNase, followed by incubating at 37 °C for 20 min to digest the template. The mixture was then mixed with 20 μL of 5× *E*-PAP Buffer, 10 μL of 25 mM MnCl_2_, 10 μL of 10 mM ATP, 4 μL of *E*-PAP enzyme, and 36 μL of nuclease-free water, followed by incubating at 37 °C for 20 min for poly(A) tailing. The tailed product was then purified using the MEGAclear Transcription Clean-Up Kit following the manufacturer’s instructions (with minor modifications). Briefly, some 20 μL of tailed RNA was mixed with 20 μL of Elution Solution, followed by mixing with 350 μL of Binding Solution Concentrate. Some 250 μL of ethanol was added to the mixture, then passed the mixture through the Filter Cartridge and washed with 250 μL of Wash Solution twice. The RNA was then eluted with 50 μL of pre-warmed (at 90 °C) Elution Solution. The sgRNAs were prepared as in the *SpCas9* mRNA preparation, except that: (a) the gRNA Cloning Vector (Cat# 41824, Addgene) was used as template, and the following programs: pre-denaturing at 98 °C for 30 s; denaturing at 98 °C for 10 s, annealing at 60 °C for 25 s, then extending at 72 °C for 20 s in each cycle; and final extending at 72 °C for 2 min; cycle number: 33; and following primers: 5′-GAAATTAATACGACTCACTATAGGCGCCCATCTTCTAGAAAGACGTTTTAGAGCTAGAAATAGC-3′, and 5′-AAAAGCACCGACTCGGTGCC-3′; were used; (b) in vitro transcription was performed using the MEGAshortscript T7 Transcription Kit, in which the mixture containing: 7.5 μL (100 ng/μL) of purified PCR product, 2 μL of T7 10× T7 Reaction Buffer, 2 μL of T7 ATP solution, 2 μL of T7 CTP solution, 2 μL of T7 GTP solution, 2 μL of T7 UTP solution, 0.5 μL of RNase inhibitor, 2 μL of T7 Enzyme Mix, and 7.5 μL of nuclease-free water was prepared. In addition, the poly(A) tailing assay was not performed.

To perform IVF on the *PDZD8*^F/F^ mouse strain (according to ref. ^112^ with modifications), the 4-week-old *PDZD8*^F/F^ female mice were intraperitoneally injected with PMSG at a dose of 10 U/mouse. At 46 h after the PMSG injection, 10 U/mouse hCG was intraperitoneally injected. At 12 h after the hCG injection, oocytes from the oviducts of female mice, along with sperms from cauda epididymides and vasa deferentia of 16-week-old, proven stud *PDZD8*^F/F^ male mice, were isolated. To isolate oocytes, oviducts were briefly left on a filter paper, followed by incubating in a human tubal fluid medium (HTF)/GSH drop on an IVF dish (prepared by placing 200 μL of HTF solution supplemented with 125 mM GSH on a 35-mm dish to form a drop, followed by covering the drop with mineral oil and pre-balancing in a humidified incubator containing 5% CO_2_ at 37 °C for 0.5 h before use). The ampulla was then torn down by forceps, and the cumulus-oocyte masses inside were collected and transferred to another HTF/GSH drop. To isolate sperms, cauda epididymides and vasa deferentia were briefly left on a filter paper, followed by penetrating with a 26 G needle on the cauda epididymides 5 times. Sperms were then released to an HTF drop on sperm capacitation dish (prepared by placing 200 μL of HTF solution on a 35-mm dish to form a drop, followed by covering the drop with mineral oil and pre-balancing in a humidified incubator containing 5% CO_2_ at 37 °C for 12 h before use) by slightly pressing/squeezing the cauda epididymides, followed by incubating in a humidified incubator containing 5% CO_2_ at 37 °C for 0.5 h. The capacitated, motile sperms (located on the edge of each HTF drop) were then collected, followed by adding to the oocyte masses soaked in the HTF/GSH drop, 8 μL per drop. The IVF dishes containing oocyte masses and sperms were then cultured in a humidified incubator containing 5% CO_2_ at 37 °C for 4 h, followed by collecting and washing oocytes in a KSOM drop (freshly prepared by placing 20 μL of KSOM medium on a 35-mm dish to form a drop, followed by covering the drop with mineral oil and pre-balancing in a humidified incubator containing 5% CO_2_ at 37 °C for 0.5 h) twice. The oocytes were then cultured in an HTF/GSH drop on an IVF dish for another 12 h in a humidified incubator containing 5% CO_2_ at 37 °C. The presumptive zygotes (in which 2 pronuclei and an extruded, second polar body could be observed) were then picked up. Some 10 pL of DNA mixture comprising *Rosa26*-*CTV-PDZD8* plasmid (20 ng/μL final concentration), *SpCas9* mRNA (120 ng/μL final concentration), and *Rosa26* sgRNA (100 ng/μL), was microinjected into each of the zygotes, and were cultured in KSOM medium at 37 °C in a humidified incubator containing 5% CO_2_. At 16 h of culturing, the zygotes/embryos at the two-cell stage were picked up and transplanted into pseudopregnant ICR female mice (8–10 weeks old, > 26 g; prepared by breeding the in-estrus female with a 14-week-old, vasectomized male at a day before the transplantation), 20 zygotes/embryos per mouse, and the offspring carrying the LSL-PDZD8 or LSL-PDZD8-T527A allele was further outcrossed 6 times to C57BL/6 mice before experiments.

The mice with muscular PDZD8 replaced with PDZD8 or its T527A mutant were validated as depicted in Supplementary information, Fig. S6b. For genotyping *Rosa26* locus, the following programs: pre-denaturing at 98 °C for 300 s; denaturing at 95 °C for 30 s, annealing at 64 °C for 30 s, then extending at 72 °C for 45 s in each cycle for 5 cycles; denaturing at 95 °C for 30 s, annealing at 61 °C for 30 s, then extending at 72 °C for 45 s in each cycle for 5 cycles; denaturing at 95 °C for 30 s, annealing at 58 °C for 30 s, then extending at 72 °C for 45 s in each cycle for 5 cycles; denaturing at 95 °C for 30 s, annealing at 55 °C for 30 s, then extending at 72 °C for 45 s in each cycle for 5 cycles; and final extending at 72 °C for 10 min; were used. For genotyping other genes and elements, the following programs: pre-denaturing at 95 °C for 300 s; denaturing at 95 °C for 30 s, annealing at 58 °C for 40 s, then extending at 72 °C for 30 s in each cycle; and final extending at 72 °C for 10 min; cycle number: 35; were used. The following primers: 5′-CGCATAACGATACCACGATATCAACAAG-3′ (Primer #1) and 5′-CCGCCTACTGCGACTATAGAGATATC-3′ (Primer #2) for cleaved *FRT*; 5′-ATCACGACGCGCTGTATC-3′ (Primer #3) and 5′-ACATCGGGCAAATAATATCG-3′ (Primer #4) for *LacZ*; 5′-ACTGTCTGTCCTTCCAGGGG-3′ (Primer #5) and 5′-GTGGAAAAGCCAAGAAAGGC-3′ (Primer #6) for *LoxP*; 5′-GCCACCTTCATGAGCTACAACACC-3′ and 5′-AACAGGAACTGGTACAGGGTCTTGG-3′ for FLPo; 5′-CAGGTAGGGCAGGAGTTGG-3′ and 5′-TTTGCCCCCTCCATATAACA-3′ for *HSA*-*Cre*; 5′-AGTGGCCTCTTCCAGAAATG-3′ and 5′-TGCGACTGTGTCTGATTTCC-3′ for the control of *HSA*-*Cre*; 5′-TCTCCCAAAGTCGCTCTGAG-3′, 5′-AAGACCGCGAAGAGTTTGTC-3′, and 5′-ATGCTCTGTCTAGGGGTTGG-3′ for *Rosa26*, 5′-GGAGTTCTATTAAGACGGTTG-3′ and 5′-GTGCTGGGTCTGTTATCTC-3′ for generating PCR products for sequencing T527. The mice with macrophagic PDZD8 replaced with PDZD8 or PDZD8-T527A were validated as described above (depicted in Supplementary information, Fig. S7a), except that *VAV1*-*Cre* (5′-GGTGTTGTAGTTGTCCCCACT-3′ and 5′-CAGGTTTTGGTGCACAGTCA-3′ for *VAV1*-*Cre-*#1; 5′-AGATGCCAGGACATCAGGAACCT-3′ and 5′-ATCAGCCACACCAGACACAGAGATC-3′ for *VAV1*-*Cre-*#2), rather than *HSA*-*Cre*, were genotyped.

The following ages of mice were used: (1) for analyzing AMPK activation: WT and *AMPKα*-MKO mice, 4 weeks old; (2) for analyzing glutaminolysis and FAO in the liver and skeletal muscle tissues: WT mice, *AMPKα*-MKO mice, and mice with muscular PDZD8 replaced with WT PDZD8 or PDZD8-T527A, aged 10 weeks; (3) for analyzing OCR in mouse skeletal muscles: WT mice, *AMPKα*-MKO mice, and mice with muscular PDZD8 replaced with WT PDZD8 or PDZD8-T527A, aged 8 weeks; (4) for determining the LPS-induced inflammatory responses, mice with macrophage PDZD8 replaced with WT PDZD8 or PDZD8-T527A, aged 8 weeks.

### Cell lines and viruses

In this study, no cell line used is on the list of known misidentified cell lines maintained by the International Cell Line Authentication Committee (https://iclac.org/databases/cross-contaminations/). HEK293T cells (Cat# CRL-3216) were purchased from ATCC. HEK293T cells and MEFs were maintained in DMEM supplemented with 10% FBS, 100 IU penicillin, 100 mg/mL streptomycin at 37 °C in a humidified incubator containing 5% CO_2_. All cell lines were verified to be free of mycoplasma contamination. HEK293T cells were authenticated by STR sequencing. PEI at a final concentration of 10 μM was used to transfect HEK293T cells. The total DNA to be transfected for each plate was adjusted to the same amount by using the relevant empty vector. Transfected cells were harvested at 24 h after transfection.

Lentiviruses, including those for knockdown or stable expression, were packaged in HEK293T cells by transfection using Lipofectamine 2000. At 30 h post-transfection, medium (DMEM supplemented with 10% FBS and MEM non-essential amino acids; approximately 2 mL) was collected and centrifuged at 5000× *g* for 3 min at room temperature. The supernatant was mixed with 10 μg/mL polybrene, and was added to MEFs or HEK293T cells, followed by centrifuging at 3000× *g* for 30 min at room temperature (spinfection). Cells were incubated for another 24 h (MEFs) or 12 h (HEK293T cells) before further treatments.

*AMPKα*^–/–^ MEFs, *RAPTOR*^–/–^ MEFs, and *AMPKα*^–/–^ HEK293T cells were generated and validated as described previously.^113^ *LAMTOR1*^F/F^, *AXIN*^F/F^, and *LKB1*^F/F^ MEFs were established by introducing SV40 T antigen via lentivirus into cultured primary embryonic cells from mouse litters as described previously,^38^ so does *GLS1*^F/F^ and *RICTOR*^F/F^ MEFs. *LAMTOR1*^–/–^, *AXIN*^–/–^, *LKB1*^–/–^, *GLS1*^–/–^ and *RICTOR*^–/–^ MEFs were generated by infecting each of MEFs with adenoviruses expressing the Cre recombinase (Cat# 1045, Vector Biolabs) for 12 h. The infected cells were then incubated in the fresh DMEM for another 12 h before further treatments. The *GLS1* gene (encoding both KGA and GAC) was knocked down and validated in MEFs as described previously.^114^ The sequence of siRNA used to knockdown mouse *RMDN3* is 5′-GAAGCCGACAAGACTTTCT-3′.

The mouse genes (*PDZD8*, *RMDN3*, *PDHA1*, *CPT1A*, and *CPT1B*) were deleted from MEFs using the CRISPR-Cas9 system. Nucleotides were annealed to their complements containing the cloning tag AAAC, and inserted into the back-to-back *Bsm*BI restriction sites of the lentiCRISPRv2 vector (#52961, Addgene). The sequence for each sgRNA is as follows: 5′-CACCCCTCGGCGCCGCCGCCATAA-3′ for *PDZD8*; 5′-TCTTATGGCGCTGCGGCGCG-3′ for *RMDN3*; 5′-GCTGTATCCCGCGTGTTGGC-3′ for *PDHA1*; 5′-GGCGGAGATCGATGCCATCA-3′ for *CPT1A*; and 5′-TCCACCGGAGTCTGGGCGAC-3′ for *CPT1B*. The constructs were then subjected to lentivirus packaging using HEK293T cells that were transfected with 2 µg of DNA in Lipofectamine 2000 transfection reagent per well of a 6-well plate. At 30 h post-transfection, the virus (approximately 2 mL) was collected to infect MEFs as described above, except cells cultured to 15% confluence were incubated with the virus for 72 h. When cells approached confluence, they were single-cell sorted into 96-well dishes. Clones were expanded and evaluated for knockout status by sequencing.

For glucose starvation, cells were rinsed twice with PBS and then incubated in glucose-free DMEM supplemented with 10% FBS and 1 mM sodium pyruvate for desired periods at 37 °C.

### Data reporting

The chosen sample sizes were similar to those used in this field: *n* = 3–10 samples to evaluate the levels of metabolites in cells and tissues^6,8,37,62,84^; *n* = 3–9 samples to determine OCR in cells and tissues,^84,115^ *n* = 3–4 samples to determine the activity of GLS1 (ref. ^73,116,117^); *n* = 3–4 samples to determine the expression levels and phosphorylation levels of a specific protein in animal cells or tissues^38^; *n* = 20–27 cells to determine protein interaction by FRET-FLIM assay in living cells^118^; *n* = 14–17 cells to determine protein interaction by PLA^119,120^; *n* = 15 to determine the septic deaths and *n* = 3–4 to determine cytokines.^121^ No statistical methods were used to predetermine the sample size. All experimental findings were repeated as stated in figure legends, and all additional replication attempts were successful. For animal experiments, mice were maintained under the same condition or place. For cell experiments, cells of each genotype were cultured in the same CO_2_ incubator and were parallel-seeded and randomly assigned to different treatments. Each experiment was designed and performed along with controls, and samples for comparison were collected and analyzed under the same conditions. Randomization was applied wherever possible. For example, during mass spectrometry analyses (metabolites and proteins), samples were processed and applied to the mass spectrometer in random orders. For animal experiments, sex-matched and age-matched litter-mate animals in each genotype were randomly assigned to different treatments. Otherwise, randomization was not performed. For example, when performing immunoblotting, samples needed to be loaded in a specific order to generate the final figures. Blinding was applied wherever possible. For example, samples or cages during sample collection and processing were labeled as code names that were later revealed by the individual who picked and treated animals or cells, but did not participate in sample collection and processing, until assessing the outcome. Similarly, during microscopy data collection and statistical analyses, the fields of view were chosen randomly and performed by different operators, preventing potentially biased selection for desired phenotypes. Otherwise, blinding was not performed, such as the measurement of GLS1 activity in vitro, as different reagents were added for particular reactions.

### Starvation and LPS treatments of mice

Unless stated otherwise, mice were housed with free access to water and a standard diet (65% carbohydrate, 11% fat, 24% protein) under specific pathogen-free conditions. The light was on from 8:00 to 20:00, with the temperature kept at 21–24 °C and humidity at 40%–70%. Only male mice were used in the study, and male littermate controls were used throughout the study.

For starvation, mice were individually caged for 1 week before each treatment. The diet was withdrawn from the cage at 17:00, and mice were sacrificed at desired time points by cervical dislocation. For LPS treatment, mice were intraperitoneally injected with LPS at 10 mg/kg as described previously.^122^ BPTES (oral gavage, 12.5 mg/kg; freshly prepared by dissolving BPTES powder in DMSO to form a 37.5 mg/mL solution, and then diluted to 1.25 mg/mL in saline), compound 968 (intraperitoneal injection, 10 mg/kg; freshly prepared by dissolving compound 968 powder in DMSO to form a 10 mg/mL solution, and then diluted to 1 mg/mL in saline) or aldometanib (oral gavage, 2 mg/kg; prepared as described previously^84^) was administered 30 min before LPS injection. Survival curves were covered up to three weeks after injection to ensure that the deaths at the late stage were not omitted. Levels of pro-inflammatory cytokines and lung damage were assessed in separate batches/sets of mice from those used for assessing survival.

### Serology

Blood glucose was measured through tail vein bleeding using the OneTouch UltraVue automatic Glucometer (LifeScan). For measuring insulin levels, approximately 100 μL of blood was collected (from the submandibular vein plexus) and was placed at room temperature for 20 min, followed by centrifugation at 3000× *g* for 10 min at 4 °C. Some 50 μL of the resultant serum was used to determine the levels of insulin using the Mouse or Rat Ultrasensitive Insulin ELISA kit according to the manufacturer’s instructions. The five-parameter logistic fitted standard curve for calculating insulin concentrations was generated from the website of Arigo Biolaboratories (https://www.arigobio.cn/ELISA-calculator/). For measuring free fatty acids, glycerol, β-hydroxybutyrate and glucagon, some 1.3, 10, 1 and 5 μL of serum were used, using the LabAssay NEFA kit, Free Glycerol Assay kit, Ketone Body Assay kit, and Mouse Glucagon ELISA Kit, respectively, all following the manufacturer’s instructions. For measuring TNFα and IL-6, some 1 μL (for IL-6) or 100 μL (for TNFα) of serum, or 100 μL of BMDM culture supernatant, was diluted to 1 mL with PBS, followed by taking 100 μL of solution to measure the cytokine levels following the manufacturer’s instructions.

### Histology

To measure the TAG content, mice were euthanized, and the gastrocnemius muscle was immediately removed and rinsed in PBS 3 times. Approximately 100 mg of tissue was homogenized in 1 mL of PBS containing 5% (v/v) Triton X-100. The homogenates were boiled for 5 min, followed by centrifugation at 20,000× *g* at 25 °C for 10 min. The TAG content (from the 3 μL of supernatant) was determined using Labassay triglyceride reagent.

Muscular glycogen contents were determined using the Glycogen Assay Kit according to the manufacturer’s instructions. Briefly, the mouse gastrocnemius muscle was quickly excised and immediately frozen in liquid nitrogen. Some 100 mg of muscle tissue was homogenized in 1 mL of double-distilled water on ice, then boiled for 5 min, followed by centrifugation at 20,000× *g* for 10 min. Then, 10 μL of supernatant was mixed with 40 μL of hydrolysis buffer, and mixed with 2 μL of hydrolysis enzyme mix, followed by incubation for 30 min at room temperature. Next, 50 μL of the master reaction mix (through mixing 46 μL of development buffer, 2 μL of development enzyme mixed with 2 μL of fluorescent peroxidase substrate) was added to the sample, followed by incubation for 30 min at room temperature in the dark. The OD_570_ was then recorded by a SpectraMax M5 microplate reader (Molecular Devices) using the SoftMax Pro software (v.5.4.1.1, Molecular Devices; and same hereafter), and the contents of glycogen were then calculated according to the standard curves generated with Glycogen Standards.

For H&E staining of lung tissues, tissues excised from blood-drained mice were cut into pieces, and were fixed in 4% (v/v) paraformaldehyde for 24 h at room temperature, then transferred to embedding cassettes. The cassettes were then washed in running water for 12 h, followed by successive soaking each for 1 h in 50% ethanol (v/v in water), 30 min in 70% ethanol, and 20 min in 95% ethanol twice. The fixed tissues were further dehydrated in anhydrous ethanol for 15 min twice, followed by immersing in 50% xylene (v/v in ethanol) for 5 min, two changes of xylene, 30 min each; and two changes of paraffin wax (Paraplast; 56 °C), 1.5 h each. The dehydrated tissues were embedded in paraffin on a HistoCore Arcadia Paraffin Embedding Machine (Leica). Paraffin blocks were then sectioned at a thickness of 5 μm, dried on an adhesion microscope slide, followed by rehydrating in the following order: three changes of xylene at 70 °C, 5 min each; two changes of anhydrous ethanol, 2 min each; two changes of 95% ethanol, 1 min each; two changes each for 1 min of 95% ethanol, and then briefly in water. The sections were then stained in hematoxylin solution for 1 min, washed in running water for 10 min, differentiated in 1% HCl (in ethanol) for 30 s, washed in running water for 1 min, immersed in 0.2% (v/v in water) ammonium hydroxide solution for 30 s, washed in running water for 1 min, and stained in eosin Y-solution for 1 min. The stained sections were dehydrated twice in 95% ethanol, 1 min each; twice in anhydrous ethanol, 1 min each; and two changes of xylene, 1 min each. The stained sections were mounted with Canada balsam and visualized on a Leica DM4 B microscope.

### Isolation and differentiation of BMDMs

BMDMs were isolated and differentiated as described previously,^123^ with modifications. Briefly, mice at 4 weeks old were anesthetized, followed by isolating the femur and tibia from the lower limbs by removing the surrounding muscles. After removing both the proximal and distal epiphyses of the femur and tibia, some 3 mL of PBS was injected into the medullary cavity via a 23 G needle from one end of each shaft, and the bone marrow of the other end was collected in a 50-mL conical tube on ice. The bone marrow suspension was then filtered by passing through a 70-μm strainer (BD Falcon), followed by centrifuge at 200× *g*, 5 min at 4 °C. The cell pellet was then resuspended in 5 mL of Red Cell Lysis Buffer for 5 min, followed by mixing with another 5 mL of RPMI 1640 medium, and then centrifuged at 500 × *g*, 5 min at 4 °C. The pellet was then resuspended in 10 mL of RPMI 1640 medium supplied with 10% inactivated FBS (heated at 56 °C, 30 min), 30% L929 conditioned medium (prepared as described previously^124^), penicillin, and streptomycin, and was cultured at 37 °C, 5% CO_2_ for 7 days, with culture medium being refreshed on day 3, for differentiation. The medium of differentiated BMDMs was then changed to RPMI 1640, supplied with 10% inactivated FBS before further uses.

For in vitro LPS stimulation, BMDM cultured in 6-well plates with a density of 2 × 10^6^ cells per well, were treated with 10 ng/mL LPS under desired glucose concentrations. After 6 h of LPS treatment, the culture supernatant was collected to detect cytokines, and the cells were lysed for immunoblotting.

### Generation of the antibody against p-T527-PDZD8

Rabbit polyclonal antibody against p-T527-PDZD8 (1:1,1000 dilution for IB) was raised using the peptide CPLSHSPKRTP(p-T)TLSI (amino acid residues 511–525 of human PDZD8) conjugated to the KLH immunogen (linked to the cysteine residue). A rabbit was then biweekly immunized with 100 µg of KLH-conjugated antigen, which is pre-incubated with 500 µg manganese adjuvant (kindly provided by Dr. Zhengfan Jiang from Peking University, see ref. ^125^) for 5 min and then mixed with PBS to a total volume of 500 µL, for 4 times, followed by collecting antiserum. The p-T527-PDZD8 antibody was then purified from the antiserum using the CPLSHSPKRTP(p-T)TLSI peptide-conjugated SulfoLink Coupling resin/column supplied in the SulfoLink Immobilization Kit. To prepare the column, 1 mg of the peptide was first dissolved with 2 mL of Coupling Buffer, followed by adding 0.1 mL of TCEP (25 mM stock concentration), then incubated at room temperature for 30 min. The mixture was then incubated with SulfoLink Resin in a column, which was pre-calibrated by 2 mL of Coupling Buffer twice on a rotator at room temperature for 15 min, followed by incubation at room temperature for 30 min without rotating. The excess peptide was then removed, and the resin was washed with 2 mL of Wash Solution 3 times, followed by 2 mL of Coupling Buffer 2 times. The nonspecific-binding sites on the resin were then blocked by incubating with 2 mL of cysteine solution (by dissolving 15.8 mg of L-cysteine-HCl in 2 mL of Coupling Buffer to make a concentration of 50 mM cysteine) on a rotator for 15 min at room temperature, followed by incubating for another 30 min without rotating at room temperature. After removing the cysteine solution, the resin was washed with 6 mL of Binding/Wash Buffer, followed by incubating with 2 mL of antiserum mixed with 0.2 mL of Binding/Wash Buffer for 2 h on a rotator. The resin was then washed with 1 mL of Binding/Wash Buffer for 5 times, and the antibody was eluted with 2 mL of Elution Buffer. The eluent was then mixed with 100 μL of Neutralization Buffer. The antibody against basal PDZD8 in the crude antibody eluent was then removed through a previously described membrane-based affinity purification method.^126^ Briefly, the bacterially purified, GST-tagged PDZD8-511–525 was subjected to SDS-PAGE, followed by transferring to a PVDF membrane. The PDZD8-bound-membrane was incubated in 5% (w/v) non-fat milk dissolved in TBST (40 mM Tris, 275 μM NaCl, 0.2% (v/v) Tween-20, pH 7.6) for 2 h, then incubated with the crude antibody preparation for 2 days, and then repeated for another 2 times. Antibody was validated for immunoblotting as shown in Supplementary information, Fig. S4g.

### Plasmids

All materials generated in this study, including mouse strains, cell lines, and antibodies, are available upon request. The expression plasmids constructed in this study have been deposited to Addgene (https://www.addgene.org/Sheng-cai_Lin/).

Full-length cDNAs used in this study were obtained either by PCR using cDNA from MEFs, or by purchasing from Origene or Sino Biological. Mutations of PDZD8 and GLS1 were performed by PCR-based site-directed mutagenesis using PrimeSTAR HS polymerase. Expression plasmids for various epitope-tagged proteins were constructed in the pcDNA3.3 vector for transfection (ectopic expression in mammalian cells), in the pBOBI vector for lentivirus packaging (stable expression in mammalian cells), in the pLVX-IRES for doxycycline-inducible expression, or the pET-28a and pGEX4T-1 (bacterial expression) vectors. PCR products were verified by sequencing (Invitrogen, China). The lentivirus-based vector pLV-H1-EF1a-puro (for GLS1) was used for the expression of siRNA in MEFs. All plasmids used in this study were purified by the CsCl density gradient ultracentrifugation method.

### IP and IB assays

To determine the interaction between endogenous GLS1 and PDZD8, four 10-cm dishes of MEFs (grown to 80% confluence) were collected for each experiment. Cells, starved or unstarved, were lysed with 500 μL per dish of ice-cold DDM/CHS lysis buffer (20 mM HEPES, pH 7.4, 50 mM NaCl, 10 mM MgCl_2_, 0.5% (w/v) DDM, 0.1% (w/v) CHS with protease inhibitor cocktail) without pre-washing with PBS (same hereafter for all IP and IB assays), followed by sonication and centrifugation at 4 °C for 15 min. Cell lysates were incubated with GLS1 or PDZD8 antibody overnight. Overnight protein aggregates were pre-cleared by centrifugation at 20,000× *g* for 10 min, and protein A/G beads (1:200 dilution, balanced with DDM/CHS lysis buffer) were then added into the lysate/antibody mixtures for another 3 h at 4 °C. The beads were centrifuged and washed with 100 times the volume of ice-cold DDM/CHS wash buffer (20 mM HEPES, pH 7.4, 50 mM NaCl, 10 mM MgCl_2_, 0.01% (w/v) DDM, 0.002% (w/v) CHS) 3 times (by centrifuging at 2000× *g*) at 4 °C and then mixed with an equal volume of 2× SDS sample buffer and boiled for 10 min before IB.

To determine the interaction between ectopically expressed GLS1 and PDZD8, a 6-cm dish of HEK293T cells were transfected with different expression plasmids. At 24 h after transfection, cells were collected and lysed in 500 µL of ice-cold DDM/CHS lysis buffer, followed by sonication and centrifugation at 4 °C for 15 min. Anti-HA-tag (1:100) or anti-Myc-tag (1:100) antibodies, along with protein A/G beads (1:100), or anti-FLAG M2 Affinity Gel (1:200, pre-balanced in DDM/CHS lysis buffer) was added into the supernatant and mixed for 4 h at 4 °C. The beads were washed with 200 times the volume of DDM/CHS wash buffer 3 times at 4 °C, mixed with an equal volume of 2× SDS sample buffer, and boiled for 10 min before immunoblotting.

To determine the interaction between stably expressed FLAG-tagged PDZD8 and endogenous 14-3-3, one 10-cm dish of MEFs (grown to 80% confluence) were collected for each experiment. IP was performed as in determining the interaction between ectopically expressed GLS1 and PDZD8, except that ice-cold Triton lysis buffer (20 mM Tris-HCl, pH 7.5, 150 mM NaCl, 1 mM EDTA, 1 mM EGTA, 1% (v/v) Triton X-100, 2.5 mM sodium pyrophosphate, 1 mM β-glycerophosphate, with protease inhibitor cocktail) was used to lyse cells and wash ANTI-FLAG M2 Affinity Gel. The M2 Affinity Gel was eluted with 30 μL of FLAG Peptide (400 μg/mL final concentration) for another 45 min at 4 °C. Some 30 μL of eluent was then collected, mixed with 7.5 μL of 5× SDS buffer, and boiled for 10 min before immunoblotting.

To verify the phosphorylation of MAM proteins (listed in Supplementary information, Table S1) by AMPK (using the anti-pan-phospho-AMPK substrate antibody), a 10-cm dish of HEK293T cells were transfected with different expression plasmids. IP was performed as in determining the interaction between ectopically expressed GLS1 and PDZD8, except that ice-cold Triton lysis buffer was used to lyse cells and wash protein A/G beads. In particular, antibodies were incubated with cell lysates for a time duration of 15 min to avoid the possible phosphorylation mediated by AMPK in the lysate (even in the unstarved cells).

The APEX2 proximity labeling assay was performed as described previously,^127^ with minor modifications. Briefly, protein biotinylation reactions in 60 10-cm dishes of MEFs with stable expression of APEX2-PDZD8 were treated with DMEM (10 mL per dish) containing 500 mM biotinyl tyramide at 37 °C for 30 min, followed by the addition of 1 mM hydrogen peroxide and incubated at room temperature for 1 min. The reactions were then terminated by removing the medium and adding ice-cold quenching buffer (10 mM sodium azide, 10 mM sodium ascorbate, 5 mM Trolox, in PBS), 10 mL per dish. Cells were washed with PBS, 10 mL per dish, followed by subcellular fractionation (see “Subcellular fractionation” section). Each fraction was lysed with 500 µL of ice-cold RIPA buffer (50 mM Tris-HCl, pH 7.5, 150 mM NaCl, 1% NP-40, 0.5% sodium deoxycholate, 0.1% SDS, with protease inhibitor cocktail), and was centrifuged at 20,000× *g* for 10 min at 4 °C. The supernatant was mixed with 20 μL of Streptavidin Magnetic Beads for 12 h on a rotator at 4 °C, then washed with 1 mL of ice-cold RIPA buffer twice. The beads were then washed with ice-cold RIPA buffer supplemented with 1 M KCl once, ice-cold RIPA buffer supplemented with 0.1 M Na_2_CO_3_ once, 2 M ice-cold urea dissolved in 10 mM Tris-HCl, pH 8.0 once, and ice-cold RIPA buffer twice. The beads slurry was then mixed with an equal volume of 2× SDS sample buffer and boiled for 10 min before immunoblotting.

To analyze the levels of p-AMPKα, p-ACC, and p-PDZD8 in MEFs or macrophages, cells grown to 70%–80% confluence in a well of a 6-well dish were lysed with 250 μL of ice-cold Triton lysis buffer. The lysates were then centrifuged at 20,000× *g* for 10 min at 4 °C, and an equal volume of 2× SDS sample buffer was added into the supernatant. Samples were then boiled for 10 min and then directly subjected to immunoblotting. To analyze the levels of p-AMPKα, p-ACC, and p-PDZD8 in muscle, liver, and heart tissues, mice were anesthetized after indicated treatments. Freshly excised (or freeze-clamped) tissues were immediately lysed with ice-cold Triton lysis buffer (10 μL/mg tissue weight for liver and 5 μL/mg tissue weight for skeletal muscle and heart), followed by homogenization and centrifugation as described above. The lysates were then mixed with 2× SDS sample buffer, boiled, and subjected to immunoblotting. All samples were subjected to immunoblotting on the same day of preparation, and any freeze-thaw cycles were avoided.

For immunoblotting, the SDS-polyacrylamide gels were prepared in-house. The thickness of the gels used in this study was 1.0 mm. Samples of less than 10 μL were loaded into wells, and the electrophoresis was run at 100 V (by PowerPac HC High-Current Power Supply, Bio-Rad) in a Mini-PROTEAN Tetra Electrophoresis Cell (Bio-Rad). In this study, all samples were resolved on 8% resolving gels, except that PDZD8-CT was run on 10% gels, and those smaller than 35 kDa including TOMM20, cytochrome C, ERLIN2 and some PDZD8 truncations on 15% gels. The resolved proteins were then transferred to the PVDF membrane (0.45 μm, Cat# IPVH00010, Merck). The PVDF membrane was then blocked by 5% (w/v) BSA (for all antibodies against phosphorylated proteins) or 5% (w/v) non-fat milk (for all antibodies against total proteins) dissolved in TBST for 2 h on an orbital shaker at 60 rpm at room temperature, followed by rinsing with the TBST for twice, 5 min each. The PVDF membrane was then incubated with a primary antibody overnight at 4 °C on an orbital shaker at 60 rpm, followed by rinsing with TBST 3 times, 5 min each at room temperature, and then the secondary antibodies for 3 h at room temperature with gentle shaking. The secondary antibody was then removed, and the PVDF membrane was further washed with TBST 3 times, 5 min each, at room temperature. PVDF membrane was incubated in an ECL mixture (by mixing equal volumes of ECL solution and Peroxide solution for 5 min), then life with Medical X-Ray Film (FUJIFILM). The films were then developed with X-OMAT MX Developer (Carestream) and X-OMAT MX Fixer and Replenisher solutions (Carestream) on a Medical X-Ray Processor (Carestream) using Developer (Model 002, Carestream). The developed films were scanned using a Perfection V850 Pro scanner (Epson) with an Epson Scan software (v.3.9.3.4), and were cropped using Photoshop 2023 software (Adobe). Levels of total proteins and phosphorylated proteins were analyzed on separate gels, and representative immunoblots were shown. Uncropped immunoblots are uploaded as a “Full scans” file.

### Determination of rates of glutaminolysis and FAO

To determine glutaminolysis and FAO rates, MEFs or mice were labeled respectively with [U-^13^C]-glutamine and [U-^13^C]-PA tracers for desired durations, followed by determination of the levels of TCA cycle intermediates through GC-MS. PA was conjugated to BSA after dissolving in 10% fatty acid-free BSA to a stock concentration of 10 mM before use.

To determine the glutaminolysis rates in MEFs, cells from one 10-cm dish (60%–70% confluence) were collected for each measurement. MEFs were glucose-starved for desired periods by incubating with triple-free (free of glucose, pyruvate, and glutamine) DMEM supplemented with 4 mM glutamine, 1 mM sodium pyruvate, 100 µM PA, 1 mM carnitine (according to ref. ^128^), and 10% FBS. At 20 min before sample collection, cells were incubated with pre-warmed triple-free DMEM supplemented with 3 mM glutamine, 1 mM [U-^13^C]-glutamine, 1 mM sodium pyruvate, 100 µM PA, 1 mM carnitine, and 10% FBS. Cells were then lysed with 1 mL of 80% methanol (v/v in water) containing 10 µg/mL myristic-d27 acid as an internal standard (IS), followed by 20 s of vortex. After centrifugation at 15,000× *g* for 15 min at 4 °C, 600 µL of each supernatant (aqueous phase) was freeze-dried in a vacuum concentrator (a LABCONCO #7310037 centrifuge connected to a LABCONCO #7460037 cold trap and an EDWARDS nXDS15i pump) at 4 °C for 24 h. The lyophilized samples were then subjected to derivatization by vortexing for 1 min after mixing each with 50 µL of freshly prepared methoxyamine hydrochloride (20 mg/mL in pyridine), followed by incubation at 4 °C for 1 h. The mixtures were sonicated at 0 °C by bathing in an ice slurry for 10 min, and were then incubated at 37 °C for 1.5 h, followed by mixing with 50 µL of MTBSTFA and incubated at 55 °C for 1 h. Before subjecting to GC-MS, samples were centrifuged at 15,000× *g* for 10 min, and some 60 μL of each supernatant was loaded into an injection vial (Cat# 5182-0714, Agilent; with an insert (Cat# HM-1270, Zhejiang Hamag Technology)) equipped with a snap cap (Cat# HM-0722, Zhejiang Hamag Technology). GC was performed on an HP-5MS column (30 m × 0.25 mm i.d., 0.25 μm film thickness; Cat# 19091S-433; Agilent) using a GC/MSD instrument (7890-5977B, Agilent) as described previously.^84^ Briefly, the injector temperature of GC/MSD was set at 260 °C. The column oven temperature was first held at 70 °C for 2 min, then increased to 180 °C at the rate of 7 °C/min, then to 250 °C at 5 °C/min, then to 310 °C at 25 °C/min, where it was held for 15 min. The MSD transfer temperature was 280 °C. The MS quadrupole and source temperature were maintained at 150 °C and 230 °C, respectively. Measurements were performed in both a scan mode (to assure the quality and purity of each TCA cycle intermediate peak) and a selected ion monitoring (SIM) mode (to maximize the sensitivity of GC-MS for quantifying each metabolite/isotopomer). In SIM mode, the fragment ion with m/z values of [M-57] (where M is the molecular mass of each derivatized metabolite, and the loss of the 57-Da facile is attributed to the loss of the *tert*-butyl moiety of the metabolite in the GC of each compound) was set as the quantitative ion. To ensure that all possible isotopomer peaks, including those of naturally occurring isotopes of a specific metabolite (with n carbon atoms), were recorded, the m/z values ranging from [M-57] to [M-57] + n + 1 were included during the data collection. In particular, for pyruvate and α-KG, m/z values from [M-57] to [M-57] + n + 2 were recorded, owing to the oximation of these two metabolites during the derivatization. The following m/z values were used for each compound: 174, 175, 176, 177 and 178 for pyruvate; 289, 290, 291, 292 and 293 for succinate; 287, 288, 289, 291 and 292 for fumarate; 346, 347, 348, 349, 350, 351 and 352 for α-KG; 419, 420, 421, 422 and 423 for malate; 418, 419, 420, 421 and 422 for aspartate; 432, 433, 434, 435, 436 and 437 for glutamate; 431, 432, 433, 434, 435, 436 for glutamine; and 591, 592, 593, 594, 595, 596 and 597 for citrate. Data were collected using the MassHunter GC/MS Acquisition software (v.B.07.04.2260, Agilent). For quantification, peaks were extracted and integrated using GC-MS MassHunter Workstation Qualitative Analysis software (v.B.07.01SP1, Agilent), and were corrected for naturally occurring isotopes using the IsoCor software^129,130^ with the matrix-based method. Rates of glutaminolysis in BMDMs were determined as described above, except that BMDMs were labeled with [U-^13^C]-glutamine for 1 h before collection.

Rates of FAO in MEFs were determined as described above for MEFs, except that MEFs were labeled with 100 µM [U-^13^C]-PA for 12 h before collection. Rates of glutamine deamination were also determined as described above, except that MEFs were labeled with 1 mM [alpha-^15^N]glutamine for 20 min, and the following m/z values were used for each compound: 431, 432, and 433 for glutamine; 432 and 433 for glutamate; 418 and 419 for aspartate; and 260 and 261 for alanine.

To determine carbon utilization in MEFs under glucose starvation, cells were separately labeled with the following isotopic tracers: (a) 1 mM [U-^13^C]-glutamine, added to the medium as described above; (b) 100 µM [U-^13^C]-PA, added to the medium as described above; all for 24 h (during which the media containing isotopic tracers were refreshed for 4 times, i.e., at 6 h, 12 h, 18 h and 22 h after labeling) to make sure the isotopic enrichment has reached steady states (been saturated; see ref. ^131^ for glutamine labeling, and ref. ^128^ for PA labeling).

Concentrations of [U-^13^C]-glutamine and [U-^13^C]-PA in the culture medium were determined as described above, except that some 100 µL of culture medium was used.

To determine the rates of glutaminolysis and FAO in mouse tissues, mice were cannulated on their right jugular veins to establish a catheter for tracer infusion at 24 h before the experiment.^132,133^ Mice were then starved for desired durations, followed by infusion with 6.87 mg/mL [U-^13^C]-glutamine and 2 mM [U-^13^C]-PA (both dissolved in 2.9 mg/mL heparin sodium salt), respectively, both at 3.3 µL/min for 2 h (titrated according to ref. ^8,134^ to achieve a pre-steady state). At the end of the infusion, the mouse was anesthetized, and 20 µL of serum, along with 100 mg of liver and muscle tissues, was collected by freeze clamping, immediately followed by freezing in liquid nitrogen. Metabolites of serum and tissues were extracted, followed by subjecting to GC-MS analysis as described above. The levels of each ^13^C-labeled metabolite were then normalized to the levels of [U-^13^C]-glutamine or [U-^13^C]-PA tracer detected in serum (see ref. ^6^).

### Determination of malonyl-CoA and glutamine

To determine levels of malonyl-CoA, HPLC-MS was performed.^84^ Briefly, MEFs collected from one 10-cm dish (grown to 60%–70% confluence) were frozen in liquid nitrogen and lysed in 1 mL of ice-cold methanol. The lysates were then mixed with 1 mL of chloroform and 400 µL of water (containing 4 µg/mL [U-^13^C]-glutamine as an IS), followed by 20 s of vortexing. After centrifugation at 15,000× *g* for another 15 min at 4 °C, 800 µL of the aqueous phase was collected, lyophilized in a vacuum concentrator at 4 °C for 8 h, and then dissolved in 30 µL of 50% (v/v, in water) acetonitrile, followed by loading 20 µL of solution into an injection vial (Cat# 5182-0714, Agilent; with an insert (Cat# HM-1270, Zhejiang Hamag Technology)) equipped with a snap cap (Cat# HM-2076, Zhejiang Hamag Technology). Measurements were based on ref. ^135^ using a QTRAP MS (QTRAP 5500, SCIEX) interfaced with a UPLC system (ExionLC AD, SCIEX). Some 2 µL of each sample were loaded onto a HILIC column (ZIC-pHILIC, 5 μm, 2.1 × 100 mm, PN: 1.50462.0001, Millipore). The mobile phase consisted of 15 mM ammonium acetate containing 3 mL/L ammonium hydroxide (> 28%, v/v) in the LC-MS grade water (mobile phase A) and LC-MS grade, 90% (v/v) acetonitrile in LC-MS grade water (mobile phase B) run at a flow rate of 0.2 mL/min. Metabolites were separated with the following HPLC gradient elution program: 95% B held for 2 min, then 45% B in 13 min, held for 3 min, and then back to 95% B for 4 min. The mass spectrometer was run on a Turbo V ion source in positive mode with a spray voltage of 5500 V. Source temperature was set at 550 °C, Gas No.1 at 50 psi, Gas No.2 at 55 psi, and curtain gas at 40 psi. Metabolites were measured using the multiple reactions monitoring mode (MRM), and declustering potentials and collision energies were optimized using analytical standards. The following transitions (parent ion/daughter ion) were used for monitoring each compound: 854/347 for malonyl-CoA and 152/88 for [U-^13^C]-glutamine. Data were collected using Analyst software (v.1.7.1, SCIEX), and the relative amounts of metabolites were analyzed using MultiQuant software (v.3.0.3, SCIEX).

To determine the intracellular glutamine levels, samples were prepared as described above, except that the ion source was set in negative mode with a spray voltage of −4500 V, and tryptophan-d5 (168.9/107.9) at 1 µg/mL final concentration was chosen as an IS. The [U-^13^C]-glutamine (149.9/114, on negative mode) dissolved in individual lysates was used to generate corresponding standard curves by plotting the ratios of labeled glutamine (areas) to IS, against the added concentrations of labeled glutamine. The amounts of intracellular glutamine were estimated according to standard curves. The average cell volume is 2000 μm^3^, as described previously.^62^

### Measurements of adenylates

Levels of AMP, ADP, and ATP were analyzed by capillary electrophoresis-based mass spectrometry (CE-MS) as described previously,^37^ with minor modifications. Briefly, each measurement required MEFs collected from one 10-cm dish (60%–70% confluence) or 100 mg of muscle tissues. Cells were washed with 25 mL of 5% (m/v) mannitol solution (dissolved in water), and were instantly frozen in liquid nitrogen. After thawing, cells were then lysed with 1 mL of methanol containing IS1 (50 µM L-methionine sulfone, 50 µM D-campher-10-sulfonic acid, dissolved in water; 1:500 (v/v) added to the methanol and used to standardize the metabolite intensity and to adjust the migration time). For analysis of adenylates in muscle, mice were anesthetized after indicated treatments. The tissue was then quickly excised by freeze-clamping, and then ground in 1 mL of methanol with IS1. The lysate was then mixed with 1 mL of chloroform and 400 μL of water, followed by 20 s of vortexing. After centrifugation at 15,000× *g* for 15 min at 4 °C, 450 μL of aqueous phase was collected and was then filtrated through a 5-kDa cutoff filter (Cat# OD003C34, PALL) by centrifuging at 12,000× *g* for 3 h at 4 °C. In parallel, quality control samples were prepared by combining 10 μL of the aqueous phase from each sample and then filtered alongside the samples. The filtered aqueous phase was then freeze-dried in a vacuum concentrator at 4 °C, and then dissolved in 100 μL of water containing IS2 (50 µM 3-aminopyrrolidine dihydrochloride, 50 µM *N,N*-diethyl-2-phenylacetamide, 50 µM trimesic acid, 50 µM 2-naphtol-3,6-disulfonic acid disodium salt, dissolved in methanol; used to adjust the migration time). A total of 20 μL of re-dissolved solution was then loaded into an injection vial (Cat# 9301-0978, Agilent; equipped with a snap cap (Cat# 5042-6491, Agilent)). Before CE-MS analysis, the fused-silica capillary (Cat# TSP050375, i.d. 50 µm × 80 cm; Polymicro Technologies) was installed in a CE/MS cassette (Cat# G1603A, Agilent) on the CE system (Agilent Technologies 7100). The capillary was then pre-conditioned with Conditioning Buffer (25 mM ammonium acetate, 75 mM diammonium hydrogen phosphate, pH 8.5) for 30 min, followed by balanced with Running Buffer (50 mM ammonium acetate, pH 8.5; freshly prepared) for another 1 h. CE-MS analysis was run at anion mode, during which the capillary was washed by Conditioning Buffer, followed by injection of the samples at a pressure of 50 mbar for 25 s, and then separation with a constant voltage at –30 kV for another 40 min. Sheath Liquid (0.1 μM hexakis(1H, 1H, 3H-tetrafluoropropoxy)phosphazine, 10 μM ammonium trifluoroacetate, dissolved in methanol/water (50% v/v); freshly prepared) flowed at 1 mL/min through a 1:100 flow splitter (Agilent Technologies 1260 Infinity II; actual flow rate to the MS: 10 μL/min) throughout each run. The parameters of the mass spectrometer (Agilent Technologies 6545) were set as: (a) ion source: Dual AJS ESI; (b) polarity: negative; (c) nozzle voltage: 2000 V; (d) fragmentor voltage: 110 V; (e) skimmer voltage: 50 V; (f) OCT RFV: 500 V; (g) drying gas (N_2_) flow rate: 7 L/min; (h) drying gas (N_2_) temperature: 300 °C; (i) nebulizer gas pressure: 8 psig; (j) sheath gas temperature: 125 °C; (k) sheath gas (N_2_) flow rate: 4 L/min; (l) capillary voltage (applied onto the sprayer): 3500 V; (m) reference (lock) masses: m/z 1,033.988109 for hexakis (1H, 1H, 3H-tetrafluoropropoxy)phosphazine, and m/z 112.985587 for trifluoroacetic acid; (n) scanning range: 50–1,100 m/z; and (n) scanning rate: 1.5 spectra/s. Data were collected using MassHunter LC/MS acquisition 10.1.48 (Agilent), and were processed using Qualitative Analysis B.06.00 (Agilent). The peak areas of adenylates were calculated using the following parameters (m/z, retention time (min)): (a) AMP: 346.0558, 9.302; (b) ADP: 426.0221, 10.930; and (c) ATP: 505.9885, 11.848. Note that the retention time of each adenylate may vary between each run and can be adjusted by isotope-labeled standards (dissolved in individual cell or tissue lysates) run between each sample, so do IS1 and IS2.

### Determination of the interaction interface between GLS1 and PDZD8

The interface between GLS1 and PDZD8 was determined through in silico docking using the FRODOCK 2.0 protein docking server (https://frodock.iqfr.csic.es/).^136^ The reported GAC structure (PDB ID: 3UO9,^137^; in which the BPTES molecule was removed from the structure) and the AlphaFold-predicted PDZD8 structure (https://alphafold.ebi.ac.uk/entry/Q8NEN9)^138^ was used. Data were then illustrated using the PyMOL (v. 2.5, Schrodinger) software. The amino acid residues P137, L139, E140, L142, Y145, G150, Q151, E152, K176, E177, D180, Q187, V209, T212, Q213, R216, K218, D223, S226, H230, F439, G444, E445, R446, V447, P450, R534, H535, F536, K538, L540, R544, and E545, a total of 33, which comprise the interface of GLS1 for PDZD8, were then mutated (all to alanine) to generate the GLS1-33A mutant.

### Determination of OCRs

For measuring OCRs in MEFs, cells were plated at 10,000 cells per well on a 96-well Seahorse XF Cell Culture Microplate (Agilent) in full medium (DMEM containing 10% FBS) overnight before the experiment, followed by glucose starvation for desired periods. For cells treated with inhibitors of glutaminolysis and FAO, etomoxir at 20 μM for 10 h and BPTES at 10 μM for 8 h were used. The medium was then changed to Seahorse XF Base Medium supplemented with 10% FBS, 25 mM glucose (not included under starvation condition, and same hereafter), 4 mM glutamine (GlutaMAX) and 1 mM sodium pyruvate 1 h before the experiment. Cells were then placed in a CO_2_-free, XF96 Extracellular Flux Analyzer Prep Station (Agilent) at 37 °C for 1 h. OCR was then measured at 37 °C in an XF96 Extracellular Flux Analyzer (Agilent), with a Seahorse XFe96 sensor cartridge (Agilent) pre-equilibrated in Seahorse XF Calibrant solution in a CO_2_-free incubator at 37 °C overnight. The assay was performed on a Seahorse XFe96 Analyzer (Agilent) at 37 °C following the manufacturer’s instruction. Concentrations of respiratory chain inhibitors used during the assay were: oligomycin A at 10 μM, FCCP at 10 μM, antimycin A at 1 μM and rotenone at 1 μM (all final concentrations). Data were collected using Wave 2.6.1 Desktop software (Agilent) and exported to Prism 9 (GraphPad) for further analysis according to the manufacturer’s instructions.

The OCR of intact muscle tissue was measured as described previously,^115^ with modifications. In brief, mice were starved for desired durations and were sacrificed through cervical dislocation. The gastrocnemius muscles from two hindlegs were then excised, followed by incubating in 4 mL of dissociation media (DM; by dissolving 50 μg/mL gentamycin, 2% (v/v) FBS, 4 mg/mL collagenase A in DMEM (21063-029)) in a 35-mm culture dish in a humidified chamber at 37 °C, 5% CO_2_, for 1.5 h. The digested muscle masses were then washed with 4 mL of pre-warmed collagenase A-free DM, incubated in 0.5 mL of pre-warmed collagenase A-free DM, and dispersed by passing through a 20 G needle 6 times. Some 20 μL of muscle homogenates were transferred to a well of a Seahorse XF24 Islet Capture Microplate (Agilent). After placing an islet capture screen by a Seahorse Capture Screen Insert Tool (Agilent) into the well, 480 μL of pre-warmed aCSF medium (120 mM NaCl, 3.5 mM KCl, 1.3 mM CaCl_2_, 0.4 mM KH_2_PO_4_, 1 mM MgCl_2_, 5 mM HEPES, 15 mM glucose, 1× MEM non-essential amino acids, 1 mM sodium pyruvate, and 1 mM GlutaMAX; adjust to pH 7.4 before use) was added, followed by equilibrating in a CO_2_-free incubator at 37 °C for 1 h. OCR was then measured at 37 °C in an XFe24 Extracellular Flux Analyzer (Agilent), with a Seahorse XFe24 sensor cartridge (Agilent) pre-equilibrated in Seahorse XF Calibrant solution (Seahorse Bioscience, Agilent) in a CO_2_-free incubator at 37 °C overnight. The respiratory chain inhibitor used during the assay was oligomycin A at 10 μM of final concentration. Data were collected using Wave 2.6.3 Desktop software (Agilent) and exported to Prism 9 (GraphPad) for further analysis according to the manufacturer’s instructions.

### Determination of electron transport chain integrity

The integrity of the electron transport chain in muscles was determined by measuring the OCR of the permeabilized myocytes supplied with an excessive substrate of each mitochondrial respiration complex on an Oxygraph-2k (Oroboros Instruments).^139^ In brief, the gastrocnemius muscle was dissected into thin fiber bundles and then immersed in ice-cold Isolation Solution A (10 mM Ca-EGTA buffer (2.77 mM CaK_2_EGTA and 7.23 mM K_2_EGTA), pH 7.1, 20 mM imidazole, 20 mM taurine, 49 mM K-MES, 3 mM K_2_HPO_4_, 9.5 mM MgCl_2_, 5.7 mM ATP, 15 mM phosphocreatine, and 1 mM leupeptin) and was then permeabilized by addition of 50 μg/mL saponin by gently mixing at 4 °C for 10 min. The permeabilized tissues were then washed three times by Respiration Medium B (0.5 mM EGTA, 3 mM MgCl_2_, pH 7.1, 20 mM taurine, 10 mM KH_2_PO_4_, 20 mM HEPES, 1 g/L BSA, 60 mM K-lactobionate, 110 mM mannitol and 0.3 mM dithiothreitol) before the assay. Some 5 mg of tissue suspended in Respiration Medium B was transferred to an oxygraphy chamber in an Oxygraph-2k (Oroboros Instruments), followed by incubation for 5 min. Glutamate (final 10 mM) and malate (5 mM) were added to the chamber to determine the resting complex I-supported respiration (without ADP addition), followed by the addition of 5 mM ADP to determine the maximal complex I-supported respiration. Succinate (10 mM) was then added to the chamber to induce the complex II-supported respiration. Data were collected using DatLab software (v.7.3.0.3, Oroboros Instruments) and exported to Prism 9 for further analysis.

The integrity of the electron transport chain in MEFs was determined on an Oxygraph-2k according to a previous study.^140^ Briefly, MEFs were glucose-starved, and were then harvested by trypsinizing, followed by centrifuged at 1000 rpm for 3 min at room temperature. The pellets were re-suspended in the mitochondrial respiration medium MiR05 buffer (110 mM sucrose, 60 mM K-lactobionate, 0.5 mM EGTA, 3 mM MgCl_2_, 20 mM taurine, 10 mM KH_2_PO_4_, 20 mM HEPES, pH 7.1 and 0.1% BSA) pre-warmed at 30 °C to a density of 0.5 × 10^6^ cells/mL, followed by transferring 2 mL of cell suspension to a chamber of Oxygraph-2k. After stabilizing for 10 min, the basal OCRs for intact cells were recorded. The baseline OCRs in permeabilized cells (also known as leak respiration, given that it is driven by the proton leak) were then determined after the addition of 1 μL of digitonin (10 mg/mL stock solution in DMSO) to the chamber to expose the electron transport chain. The resting complex I-supported respiration was then recorded after adding 5 mM pyruvate, 10 mM glutamate, and 2 mM malate to the chamber, followed by determining the maximal complex I-supported respiration through the addition of 2.5 mM ADP. Succinate (10 mM) was then added to the chamber to induce complex II-supported respiration. The maximal respiratory capacity was then determined by stepwise addition of FCCP to a final concentration of 0.5 μM. Data were collected using DatLab software (v.7.3.0.3, Oroboros Instruments) and exported to Prism 9 for further analysis.

### Confocal microscopy

The filamentation of GLS1 under glucose or glutamine starvation was determined as described previously.^141^ Briefly, MEFs grown to 80% confluence on coverslips in 6-well dishes were fixed for 20 min with 4% (v/v) formaldehyde in PBS, 2 mL per well/coverslip at room temperature. The coverslips were rinsed twice with 2 mL of PBS and permeabilized with 2 mL of 0.1% (v/v) Triton X-100 in PBS for 10 min at 4 °C. After rinsing twice with 2 mL of PBS, the coverslips were incubated with rabbit anti-GLS1 antibody (1:100, diluted in Block Buffer (10% (v/v) NGS in PBS, with 0.1% (w/v) saponin) overnight (by placing 50 μL of antibody solution on a piece of Parafilm M to form a drop, followed by mounting a coverslip on the drop) in a humidified chamber at 4 °C. The cells were then rinsed three times with 2 mL of PBS, and then incubated with a secondary antibody (Alexa Fluor 488 donkey anti-rabbit IgG; performed as in primary antibody incubation) for 8 h at room temperature in a humidified chamber in the dark. The coverslips were washed another 4 times with 2 mL of PBS, and then mounted on slides using ProLong Diamond Antifade Mountant. Confocal microscopic images were taken using an LSM 980 (Zeiss) with a 63× 1.4 NA oil objective, during which a diode laser module (Lasos) at 488 nm was used to excite Alexa Fluor 488 dye. All parameters were kept unchanged for each picture taken. Images were processed and analyzed on Zen Blue 3.3 software (Zeiss), and formatted on Photoshop 2023 software (Adobe).

The PLA/Duolink assay was performed using the Duolink In Situ Red Starter Kit (Mouse/Rabbit) according to the manufacturer’s instruction, with minor changes. In brief, MEFs expressing HA-tagged PDZD8 (or its mutants) were grown to 80% confluence on coverslips in 6-well dishes, followed by fixation for 20 min with 4% (v/v) formaldehyde in PBS, 2 mL per coverslip/well at room temperature. The coverslips were rinsed twice with 2 mL of PBS and permeabilized with 2 mL of 0.1% (v/v) Triton X-100 in PBS for 10 min at 4 °C. Cells were then blocked with Duolink Blocking Solution (50 μL per coverslip) in a humidified chamber at 37 °C for 1 h. Cells were then incubated with primary antibodies (mouse anti-HA-tag and rabbit anti-GLS1; 1:100 diluted with Duolink Antibody Diluent; 50 μL per coverslip) in a humidified chamber at 4 °C for 12 h, followed by washing with two changes of 2 mL of Wash Buffer A, 5 min per change, at room temperature. The coverslip was then incubated with PLUS and MINUS PLA probe solution (freshly prepared by mixing 10 μL of PLA probe MINUS stock, 10 μL of PLA probe PLUS stock with 30 μL of Duolink Antibody Diluent; 50 μL per coverslip) in a humidified chamber at 37 °C for 1 h, followed by washing with two changes of 2 mL of Wash Buffer A, 5 min per change, at room temperature. The coverslip was then incubated with Ligation Solution (freshly prepared by 1:5 diluting Duolink Ligation buffer with water, followed by the addition of Ligase stock at a ratio of 1:50; 50 μL per coverslip) in a humidified chamber at 37 °C for 0.5 h, followed by washing with two changes of 2 mL of Wash Buffer A, 5 min per change, at room temperature. The coverslip was then incubated with Amplification Solution (freshly prepared by 1:5 diluting Amplification buffer with water, followed by addition of Polymerase stock at a ratio of 1:80; 50 μL per coverslip) in a humidified chamber at 37 °C for 100 min, followed by washing with two changes of 2 mL of Wash Buffer B, 10 min per change, at room temperature. The coverslip was then washed with 2 mL of 0.01× Wash Buffer B for 1 min at room temperature, followed by mounting with 15 μL of Duolink PLA Mounting Medium with DAPI for 30 min, and then subjected to imaging using an LSM 980 (Zeiss) as described above, except that a DPSS laser module (Lasos) at 594 nm and a diode laser module (Lasos) at 405 nm were used to excite the PLA and DAPI, respectively.

### FRET-FLIM assay

FRET-FLIM experiments were carried out as described previously,^118^ with minor modifications. Briefly, MEFs stably expressing GFP (“donor only”, as a control), RFP-PDZD8-GFP, or different combinations of GFP-PDZD8 and GLS1-mCherry were cultured in 35-mm glass-bottom dishes (Cat# D35-20-10-N, In Vitro Scientific) to 60%–80% confluence. Cells were starved for glucose or not, followed by determining the fluorescence lifetime of GFP in different cells cultured in a humidified chamber with 5% CO_2_ at 37 °C using a STELLARIS 8 FALCON (Leica) systems equipped with HyD X and HyD SMD detectors and an HC PL APO CS2 63×/1.40 Oil objective (Leica). Cells were excited with a 460-nm laser via the systems’ tunable White Light Laser (WLL), and photon arrival times were recorded with a HyD X detector covering the GFP emission spectrum (460–510 nm). All parameters were kept unchanged between imaging. Images were taken and analyzed by LAS X Software (Leica). In all experiments, the position of the focal plane was actively stabilized using the Leica Auto Focus Control (AFC) to prevent any focal drift or focus artifacts.

### SIM and STORM imaging

MEFs grown to 50% confluence in a 35-mm dish (for SIM; Cat# D35-20-10-N, In Vitro Scientific), or in Lab-Tek II chambered no. 1.5 German coverglass system (for STORM; Cat# 155409, 8 Chamber, Nunc) were treated following the Semi-intact IF protocol described previously,^39^ with minor modifications. Briefly, cells were rinsed with PBS once, and treated with Buffer I (25 mM HEPES, pH 7.2, 125 mM potassium acetate, 5 mM magnesium acetate, 1 mM DTT, 1 mg/L glucose, and 25 μg/mL digitonin) for 1 min on ice, and then Buffer II (25 mM HEPES, pH 7.2, 125 mM potassium acetate, 5 mM magnesium acetate, 1 mM DTT and 1 mg/L glucose) for another 10 min on ice. The cells were then fixed with ice-cold methanol in PBS on ice for 10 min. The slides were rinsed twice with PBS, and cells were then permeabilized with 0.05% Triton X-100 in PBS for 5 min at 4 °C. After rinsing twice with PBS, the slides were blocked in Block Buffer for 30 min. The slides were washed twice with PBS and incubated with primary antibodies diluted in Block Buffer overnight at 4 °C. The cells were then rinsed three times with PBS, and then incubated with secondary antibodies for another 8 h at 4 °C in the dark, followed by washing for four times with PBS before imaging.

SIM images were acquired using a Multi-SIM (multimodality structured illumination microscopy) imaging system (NanoInsights-Tech Co., Ltd.) equipped with a 100×/ 1.49NA oil objective (CFI SR HP Apo, Nikon), a solid-state, single-mode laser (containing the 488-nm, 561-nm and 640-nm laser beams) and an sCOMS (complementary metal-oxide-semiconductor) camera (ORCA-Fusion C15440-20UP, HAMAMATSU). The immersion oil with a refractive index of 1.518 was chosen for this experiment, and the microscope was calibrated with 100-nm fluorescent spheres before the experiment. The SIM images were taken through the low NA GI-SIM mode with a 50-mw laser power and a 20-ms exposure time via SI-Recon 2.11.19 software (NanoInsights-Tech). Images were then reconstructed using the SI-Recon 2.11.19 software, during which the parameters were set as: (a) pixel size: 30.6 nm; (b) optical transfer functions: channel-specific; (c) Wiener filter: constant 0.01, for the TIRF-SIM mode; and (d) negative intensities background: discard. After reconstruction, images were denoised under the total variation constraint mode. The denoised images were then formatted using Photoshop 2023 software (Adobe).

STORM imaging was performed as described previously,^39,142^ with minor modifications. Briefly, the STORM imaging buffer supplemented with MEA was freshly prepared before the experiment by mixing 7 μL of GLOX (14 mg of glucose oxidase, 50 μL of catalase (17 mg/mL), 200 μL of buffer A (10 mM Tris, pH 8.0 and 50 mM NaCl), vortexed to dissolve and cooled on ice) with 70 μL of 1 M MEA (77 mg of MEA dissolved in 1.0 mL of 0.25 M HCl), followed by adding to 620 μL of buffer B (50 mM Tris, pH 8.0, 10 mM NaCl and 10% (m/v) glucose) in a 1.5-mL Eppendorf tube, and followed by brief vortex. The mixture was then added to each well, and images were taken on an N-STORM (Nikon). The imaging was performed using an inverted microscope system (Ti-E Perfect Focus; Nikon) equipped with a monolithic laser combiner (MLC400, Agilent) containing solid-state lasers of wavelengths 405 nm (at 100 mW of maximum fiber output power), 488 nm (200 mW) and 561 nm (150 mW) and a 647-nm laser at 300 mW. After locating a suitable field, a diffraction-limited TIRF image was acquired for reference, followed by a STORM acquisition. The 647-nm laser was then sequentially fired at 100% power to excite all possible fluorophore molecules and photoswitch them into a non-emitting dark state, and then the 561-nm laser. The emitted wavelengths from Alexa Fluor 647 and CF 568 fluorophores were then sequentially collected by the plan-Apochromat 100×/1.49NA TIRF objective (Nikon), filtered by an emission filter set (FF01-586/20-25 × 3.5 and FF01-692/40-25; Semrock), and detected on an electron-multiplying charge-coupled device camera (iXon DU-897, Andor Technology). During imaging, 20,000 sequential frames of each channel were acquired. The image acquisition, lateral drift correction, and data processing were performed using NIS Elements software with STORM package (v.4.30 build 1053, Nikon) as previously described.^143^

### Subcellular fractionation

Mitochondria and MAMs were purified as described previously,^144^ with minor modifications.^62^ Briefly, 40 10-cm dishes of MEFs (60%–80% confluence) were collected by scrapping at room temperature, followed by centrifugation for 5 min at 500× *g* at 37 °C. Cells were then resuspended in 20 mL of ice-cold IB_cells_-1 buffer (225 mM mannitol, 75 mM sucrose, 0.1 mM EGTA, and 30 mM Tris-HCl, pH 7.4), and dounced for 100 strokes in a 40-mL Dounce homogenizer (using the small clearance pestle, or the pestle B; Cat# D9188, Sigma), followed by two times of centrifugation for 5 min at 600× *g* at 4 °C. The supernatants were then collected and centrifuged for 10 min at 7000× *g* at 4 °C. The pellets were then washed twice with 20 mL of ice-cold IB_cells_-2 buffer (225 mM mannitol, 75 mM sucrose, and 30 mM Tris-HCl pH 7.4). The suspensions were centrifuged at 7000× *g*, and again at 10,000× *g*, both for 10 min at 4 °C. The pellets were then resuspended in 2 mL of ice-cold MRB buffer (250 mM mannitol, 5 mM HEPES pH 7.4, and 0.5 mM EGTA), and were loaded on top of 10 mL of Percoll medium (225 mM mannitol, 25 mM HEPES pH 7.4, 1 mM EGTA and 30% Percoll (v/v)) in 14 × 89-mm centrifuge tubes (Cat# 344059, Beckman). The tubes were then centrifuged on a SW 41 Ti rotor (Beckman) at 95,000× *g* for 0.5 h at 4 °C. After centrifugation, the dense band located near the bottom of each tube was collected as mitochondrial fraction, while the band located at the interface between MRB and Percoll cushions as MAMs. The mitochondrial fractions were diluted with 10 volumes of MRB buffer, followed by centrifugation at 6300× *g* for 10 min at 4 °C; the pellets were resuspended and washed with 2 mL of MRB buffer, followed with centrifugation at 6300× *g* for 10 min at 4 °C to obtain pure mitochondria (the pellets). The MAM fractions were centrifuged at 6300× *g* for 10 min at 4 °C, and the supernatants were combined and transferred to 25 × 83-mm centrifuge tubes (Cat# 344367, Beckman), followed by centrifuge at 95,000× *g* on a SW 32 Ti rotor (Beckman) for 1 h at 4 °C to obtain the pure MAM (the pellets).

OMM was purified by suspending pure mitochondria with 100 μL of MRB buffer containing 0.5% (w/v) digitonin, followed by centrifuge at 10,000× *g* for 15 min at 4 °C. The supernatant contains OMM, and the pellet mitoplast. The mitoplasts were suspended with 100 μL of MRB buffer containing 1% (v/v) Triton X-100, followed by centrifuge in an 8 × 34-mm centrifuge tube (Cat# 45235-AV, ThermoFisher Scientific) at an S120-AT3 rotor (ThermoFisher Scientific) at 100,000× *g* for 30 min at 4 °C, and the pellets and supernatants contain IMM and matrix, respectively.

ER was purified according to the protocol optimized by combining the traditional microsome-based density gradient isolation method (Endoplasmic Reticulum Isolation Kit developed by Sigma) with the cell surface biotinylation reaction method (developed and optimized by Pierce), and was described previously.^39^ Briefly, MEFs from 40 10-cm dishes (80% confluence) were quickly washed with ice-cold PBS (10 mL each dish) twice, followed by incubating with 250 μg/mL of sulfo-NHS-SS-biotin (freshly dissolved in ice-cold PBS, 10 mL each dish) for 30 min with gentle agitation on an orbital shaker at 4 °C. Some 500 μL of 1 M Tris (pH 8.0 at 4 °C) was then added to each dish to quench the biotinylation reaction. Cells were collected afterward by scrapping, followed by centrifugation at 600× *g* for 5 min, and then washed with 40 mL of ice-cold PBS twice. Cells were then re-suspended in 10 mL of 1× Hypotonic Extraction Buffer and then incubated at 4 °C for 30 min, with gentle mixing in the middle. Cells were then centrifuged at 600× *g* at 4 °C for 5 min, and the pellet was re-suspended with 6 mL of 1× Isotonic Extraction Buffer, followed by mixing in a 7-mL Dounce homogenizer (using the small clearance pestle; Cat# D9063, Sigma) for 10 strokes. The homogenates were centrifuged at 1000× *g* for 10 min at 4 °C, and the supernatants (PNS) were further centrifuged at 12,000× *g* for 15 min at 4 °C, yielding the supernatants as the post-mitochondrial fraction (PMF). The PMF was loaded in two 11 × 60 mm centrifuge tubes (Cat# 344062, Beckman) and then centrifuged on an SW 60 Ti rotor (Beckman) at 100,000× *g* for 1 h at 4 °C. The pellet was re-suspended with 0.5 mL of 1× Isotonic Extraction Buffer, and was mixed in a 2-mL Dounce homogenizer (using the small clearance pestle; Cat# D8938, Sigma) for 20 strokes, yielding the microsomal suspension. The suspension was mixed with 0.25 mL of OptiPrep, and was carefully layered on the top of 1 mL of 30% OptiPrep solution (by mixing 0.5 mL of OptiPrep with 0.5 mL of 1× Isotonic Extraction Buffer) in an 11 × 60 mm centrifuge tube. Some 2 mL of 15% OptiPrep solution (by mixing 0.5 mL of OptiPrep with 1.5 mL of 1× Isotonic Extraction Buffer) was then carefully layered on the top of the sample. The tube was then centrifuged on an SW60 Ti rotor at 150,000× *g* for 3 h at 4 °C. The top 0.6 mL of 15% OptiPrep solution was discarded, and the remaining 200 μL of the fraction was collected as the crude ER fraction. The fraction was then incubated with 100 μL of NeutrAvidin Agarose (pre-balanced by 1× Isotonic Extraction Buffer) for another 2 h. The supernatant contains ER fractions.

### Identification of AMPK substrates in MAM

To identify the substrate(s) of AMPK in the mitochondria and MAM, the mitochondria and MAM fractions purified from 120 10-cm dishes of glucose-starved MEFs were dissolved with 5 mL of ice-cold Triton lysis buffer, followed by sonication and centrifugation at 4 °C, 20,000× *g* for 15 min. The lysates were incubated with anti-pan-phospho-AMPK-substrates antibodies overnight. Protein aggregates were pre-cleared by centrifugation at 20,000× *g* for 10 min, and protein A/G beads (1:250, pre-balanced with Triton lysis buffer) were then added into the lysate-antibody mixture, and incubated for another 3 h at 4 °C with rotating at 60 rpm The beads were centrifuged and washed with 100 times volume of Triton lysis buffer for 3 times (by centrifuging at 2000× *g*) at 4 °C and then mixed with an equal volume of 2× SDS sample buffer (without bromophenol blue addition), and boiled for 10 min before subjecting to SDS-PAGE. After staining with Coomassie Brilliant Blue R-250 dye, gels were decolored, and the excised gel segments were subjected to in-gel chymotrypsin digestion and then dried. Samples were analyzed on a nanoElute (Bruker) coupled to a timsTOF Pro (Bruker) equipped with a CaptiveSpray source. Peptides were dissolved in 10 μL of 0.1% formic acid (v/v) and were loaded onto a homemade C18 column (35 cm × 75 μm, ID of 1.9 μm, 100 Å). Samples were then eluted with linear gradients of 3%–35% acetonitrile (v/v, in 0.1% formic acid) at a flow rate of 0.3 μL/min, for 60 min. MS data were acquired with a timsTOF Pro mass spectrometer (Bruker) operated in PASEF mode, and were analyzed using Peaks Studio software (X^+^, Bioinformatics Solutions). The mouse UniProt Reference Proteome database was used for data analysis, during which the parameters were set as: (a) precursor and fragment mass tolerances: 20 ppm and 0.05 Da; (b) semi-specific digest mode: allowed; (c) maximal missed cleavages per peptide: 3; (d) variable modifications: oxidation of methionine, acetylation of protein N-termini, and phosphorylation of serine, threonine, and tyrosine; (e) fixed modification: carbamidomethylation of cysteine.

### Prokaryotic protein expression

The expression plasmid for the His-tagged, heterotrimeric AMPK was kindly provided by Dr. Dietbert Neumann, and the plasmid for rat kinase domain (KD) of CaMKK2 (amino acid residues 129–503) by Dr. Anthony Means. The cDNAs encoding human AMPKα1-KD (amino acid residues 27–290)^145^ and rat CaMKK2-KD^39^ were inserted into the pET-28a (Novagen) vectors for expressing His-tagged recombinant proteins as described previously. The expression plasmids for the two isozymes of GLS1, PDZD8, and mutants were constructed by inserting respective cDNAs into pGEX-4T-1 (Cytiva) vectors for expressing GST-tagged recombinant proteins. *KGA* and *GAC* cDNAs were also cloned into the pET-28a vectors for bacterial expression. The pET-28a and pGEX-4T-1 plasmids were transformed into the *E*. *coli* strain BL21 (DE3) (Cat# EC0114, ThermoFisher Scientific), followed by culturing in LB medium in a shaker at 200 rpm at 37 °C. The cultures of transformed cells were induced with 0.1 mM IPTG at an OD_600_ of 1.0. After incubating for another 12 h at 160 rpm at 16 °C, the cells were collected. For His-tagged proteins, cells were homogenized in a His binding buffer (50 mM sodium phosphate, pH 7.4, 150 mM NaCl, 1% Triton X-100, 5% glycerol, and 10 mM imidazole), and for GST-tagged proteins, with a GST binding buffer (PBS supplemented with 10 mM β-mercaptoethanol and 1% Triton X-100) on ice. The homogenates were then sonicated on ice and were subjected to centrifugation at 150,000 × *g* for 30 min at 4 °C, followed by purification of His-tagged proteins with Nickel Affinity Gel (pre-balanced with His binding buffer), or GST-tagged proteins with Glutathione Sepharose 4 Fast Flow Gel (pre-balanced with GST binding buffer) at 4 °C. The Nickel Affinity Gel was then washed with 100 times the volume of ice-cold His wash buffer (50 mM sodium phosphate, pH 7.4, 150 mM NaCl, and 20 mM imidazole), and the Glutathione Sepharose gel with 100 times the volume of ice-cold PBS. His-tagged proteins were eluted from the resin by His elution buffer (50 mM sodium phosphate, pH 7.4, 150 mM NaCl, and 250 mM imidazole), and GST-tagged proteins by GST elution buffer (50 mM Tris–HCl, pH 8.0, and 10 mM reduced glutathione) at 4 °C. In particular, to avoid the degradation of the full-length PDZD8 protein, a relatively large volume of GST binding buffer (i.e., a diluted bacteria homogenate, e.g., 300 mL of GST binding buffer for 3600 mL of bacteria suspension at an OD_600_ of 1.0), a low sonication power (e.g., < 25% maximal power output on a VCX 750 (Sonics) sonicator equipped with a 1/4” (6-mm) stepped microtip (630-0435, Sonics)), and a shorter sonication duration (100 cycles of 3 s pulse with 3 s interval for 50 mL of bacteria homogenate) were applied. Proteins were concentrated to approximately 3 mg/mL by ultrafiltration (Millipore, UFC905096) at 4 °C, then subjected to gel filtration (Cytiva, Superdex 200) balanced with a buffer containing 50 mM Tris-HCl, pH 7.4 and 150 mM NaCl.

### Phosphorylation of PDZD8 by AMPK in vitro

For those experiments using heterotrimeric AMPK complex as the kinase in the assay system, the AMPK complex was pre-activated by CaMKK2-KD as described previously,^39^ with minor modifications. Briefly, GST-tagged CaMKK2-KD was incubated with Glutathione Sepharose 4 Fast Flow Gel (5 μg of protein/μL gel; pre-balanced with PBS) in PBS at 4 °C for 1 h, followed by washing with 100 times the volume of ice-cold CaMKK2 kinase assay buffer (50 mM Tris, pH 8.0, 2 mM DTT, 100 mM NaCl, 10 mM MgCl_2_ and 1 mM ATP) twice. Some 1 μL of gel-immobilized CaMKK2 was then incubated with 5 μg of His-tagged AMPK complex in CaMKK2 kinase assay buffer supplemented with 5 mM ATP (total volume: 60 μL) on a thermomixer at 30 °C for 30 min. CaMKK2 was then removed by centrifugation at 2000× *g*, 4 °C for 30 s, yielding phosphorylated AMPK in the supernatant. For those experiments using His-tagged AMPK-KD as the kinase, proteins were directly subjected to the assay without being pre-phosphorylated by CaMKK2.

Phosphorylated PDZD8 proteins were prepared in an AMPK kinase assay system as described previously.^145^ Briefly, 50 μg of GST-tagged PDZD8 was incubated with 10 μL of Glutathione Sepharose gel (pre-balanced with PBS) in PBS at 4 °C for 1 h, followed by washing with 100 times the volume of ice-cold PBS twice. The gel was then incubated with 5 μg of AMPK-KD or phosphorylated AMPK complex in 250 μL of AMPK kinase assay buffer (50 mM MOPS, pH 7.0, 100 mM NaCl, 0.1 mM EDTA, 10 mM MgCl_2_, and 5 mM ATP) at 25 °C for 2 h, followed by washing with 100 times the volume of ice-cold PBS twice, and PDZD8 proteins were eluted with GST elution buffer. The eluents were either subjected to immunoblotting or to assays for enzymatic activities of GLS1 (see below).

### Enzymatic activity

Activity of GLS1 in cell-free system was determined through a GLS1-GDH-coupled assay system as described previously.^73^ Briefly, in each reaction, 10 nM GLS1 and 100 nM PDZD8 were pre-incubated in 100 μL of Reaction buffer (50 mM Tris-acetate, pH 8.6 and 0.2 mM EDTA) for 2 h on a rotator at 4 °C, followed by mixing with 3 U of GDH and 40 mM NAD^+^ in 10 μL of Reaction buffer at 25 °C for 30 min. The reaction was initiated by pipetting the GLS1–PDZD8 mixture into a well of a glass-bottom, 96-well microplate (Cat# 3635, Corning) containing 90 μL of glutamine solution (at a desired concentration used in the assay; dissolved in Reaction buffer) pre-warmed at 25 °C, followed by mixing on a SpectraMax M5 microplate reader (Molecular Devices). For the assays performed in the presence of phosphate, K_2_HPO_4_ (2 M stock, pH 9.4), at a final concentration of 20 mM, was added into the glutamine solution. The effects of PDZD8 on GLS1 activity were assessed with the initial velocities of NADH formation during the reaction through which the OD_340_, recorded at 30-s intervals on a SpectraMax M5 microplate reader using the SoftMax Pro software, was increased. All measurements were carried out in triplicate. The catalytic velocities were calculated by the extinction coefficient for NADH at 340 nm, which is 6220 cm^–1^ M^–1^, and 0.625 cm for path length. Data were collected using the SoftMax Pro software and exported to OriginPro software (v.9.2.0, OriginLab) for further analysis.

Activities of GLS1 in cells were determined through a semi-permeabilized system. Some 24 h before the measurement, MEFs were seeded in 24-well dishes (Cat# 142485, ThermoFisher Scientific), and were cultured to 90% confluence. Cells were then starved for glucose for desired periods, followed by gentle rinsing with 350 μL of PBS containing 0.01% (v/v) NP-40 (titrated according to the conditions described in ref. ^90^) and 1% (v/v) protease inhibitor cocktail for each well for 60 s at 25 °C. The permeabilized cells were then quickly washed with 300 μL of PBS twice to remove the detergents, and the reaction was initiated by pipetting 200 μL of Reaction Buffer (20 mM glutamine dissolved in PBS), or Reaction Buffer supplemented with 10 μM BPTES for determining baseline, into each well, followed by mixing on a thermomixer (Thermomixer R, Eppendorf) at 37 °C, 50 rpm for 1 h. For the validation assays with transaminases and glutamate dehydrogenases inhibited, 20 μM AOA and 20 μM R162 were included in the Reaction Buffer. The glutamate yielded was then measured using the Glutamate Assay Kit according to the manufacturer’s instructions. In brief, 100 μL of Reaction Buffer in each well was collected, followed by centrifugation at 20,000× *g* for 10 min. Some 20 μL of supernatant was collected, and then mixed with 30 μL of Glutamate Assay Buffer, followed by incubating with 100 μL of Reaction Mix (prepared by mixing 8 μL of Glutamate Developer and 2 μL of Glutamate Enzyme Mix in 90 μL of Glutamate Assay Buffer) at 37 °C for 30 min in the dark. The OD_450_ was then recorded by a SpectraMax M5 microplate reader using the SoftMax Pro software.

### Statistical analysis

Statistical analyses were performed using Prism 9 (GraphPad Software), except for the survival curves, which were analyzed using SPSS 27.0 (IBM). Each group of data was subjected to the Kolmogorov-Smirnov test, Anderson-Darling test, D’Agostino-Pearson omnibus test, or Shapiro–Wilk test for normal distribution when applicable. An unpaired two-tailed Student’s *t*-test was used to determine the significance between two groups of normally distributed data. Welch’s correction was used for groups with unequal variances. An unpaired two-tailed Mann–Whitney test was used to determine the significance between data without normal distribution. For comparisons between multiple groups with one fixed factor, an ordinary one-way ANOVA or one-way repeated-measures ANOVA (for blood glucose data) was used, followed by Tukey, Sidak, Dunnett, or Dunn as specified in the legends. The assumptions of homogeneity of error variances were tested using *F*-test (*P* > 0.05). For comparison between multiple groups with two fixed factors, an ordinary two-way ANOVA was used, followed by Tukey’s or Sidak’s multiple comparisons test as specified in the legends. Geisser-Greenhouse’s correction was used where applicable. The adjusted means and SEM, or SD, were recorded when the analysis met the above standards. Differences were considered significant when *P* < 0.05, or *P* > 0.05, with large differences in observed effects (as suggested in^146,147^).

## Supplementary information

Supplementary information, Fig. S1

Supplementary information, Fig. S2

Supplementary information, Fig. S3

Supplementary information, Fig. S4

Supplementary information, Fig. S5

Supplementary information, Fig. S6

Supplementary information, Fig. S7

Supplementary information notes

Supplementary information, Table S1

Supplementary information, Table S2

Supplementary information, Table S3

Full scans

## Source data

Source data

## Data Availability

The MS proteomics data have been deposited to the ProteomeXchange Consortium (http://proteomecentral.proteomexchange.org) through the iProX partner repository^148,149^ with the dataset identifier PXD041428. Full immunoblots are provided as a “Full scans” file. Raw data and the statistical analysis data are also provided in this study as a “Source data” file. This manuscript does not report the original code. Any additional information required to reanalyze the data reported in this paper is available upon request.
