# Supplementary material for: AMPK targets PDZD8 to trigger carbon source shift from glucose to glutamine
Source: Cell Res. 2024 Jun 19;34(10):683–706. doi: 10.1038/s41422-024-00985-6 (PMC11442470; doi:10.1038/s41422-024-00985-6)
Supplement: Supplementary file 1 — Supplementary information, Fig. S1 [file 41422_2024_985_MOESM1_ESM.pdf]

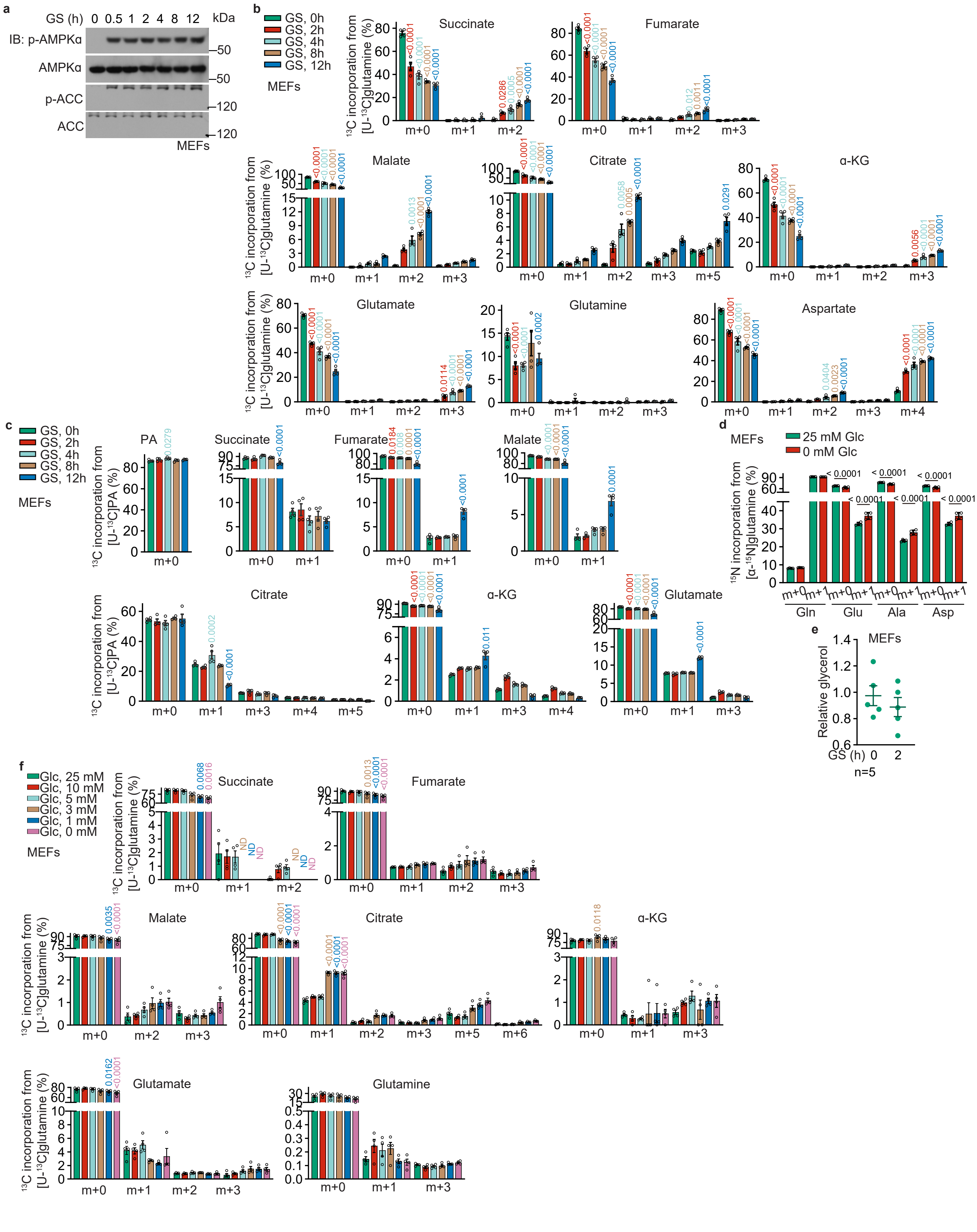

Supplementary information, Fig. S1

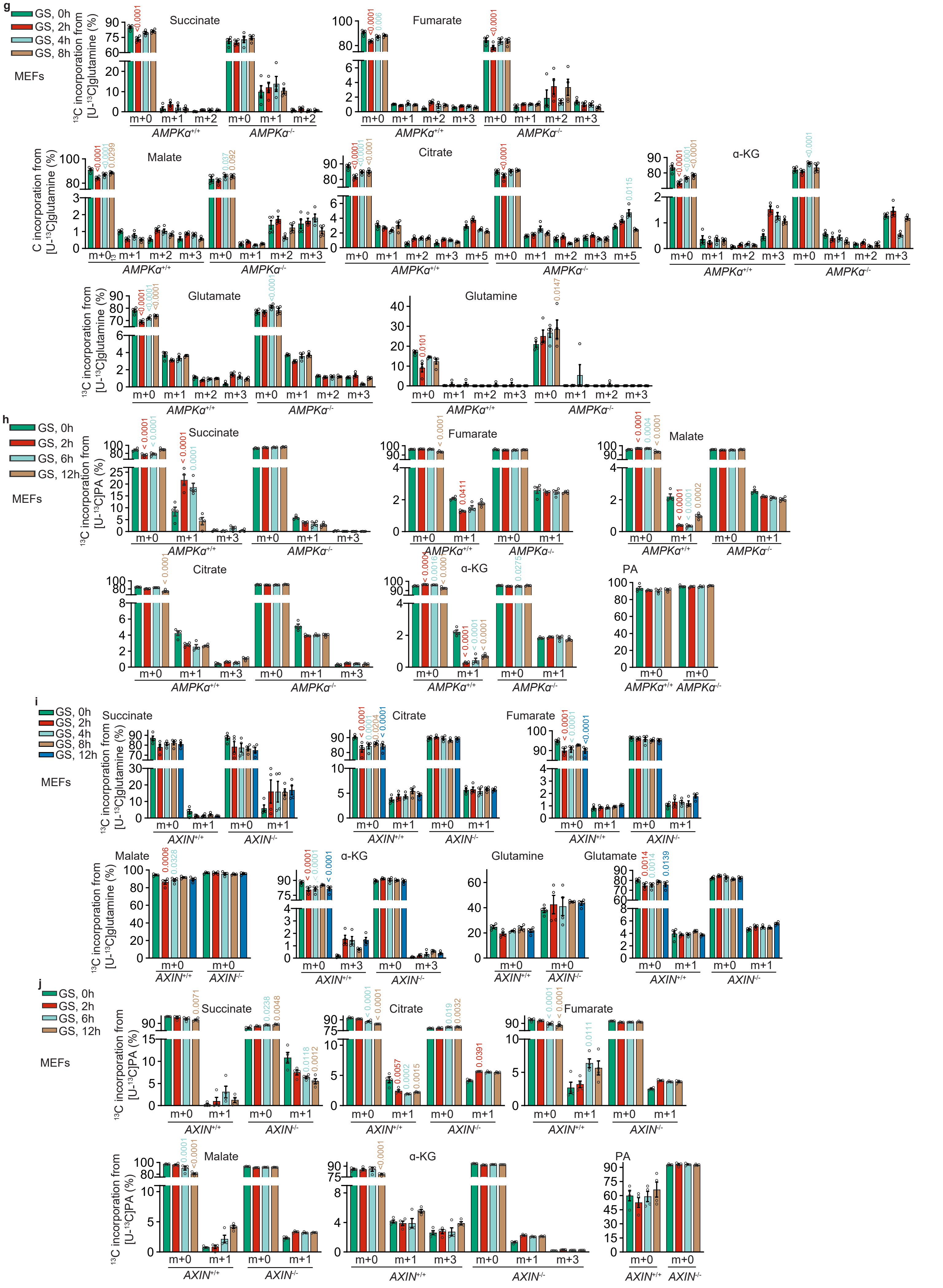

Supplementary information, Fig. S1 (cont.)

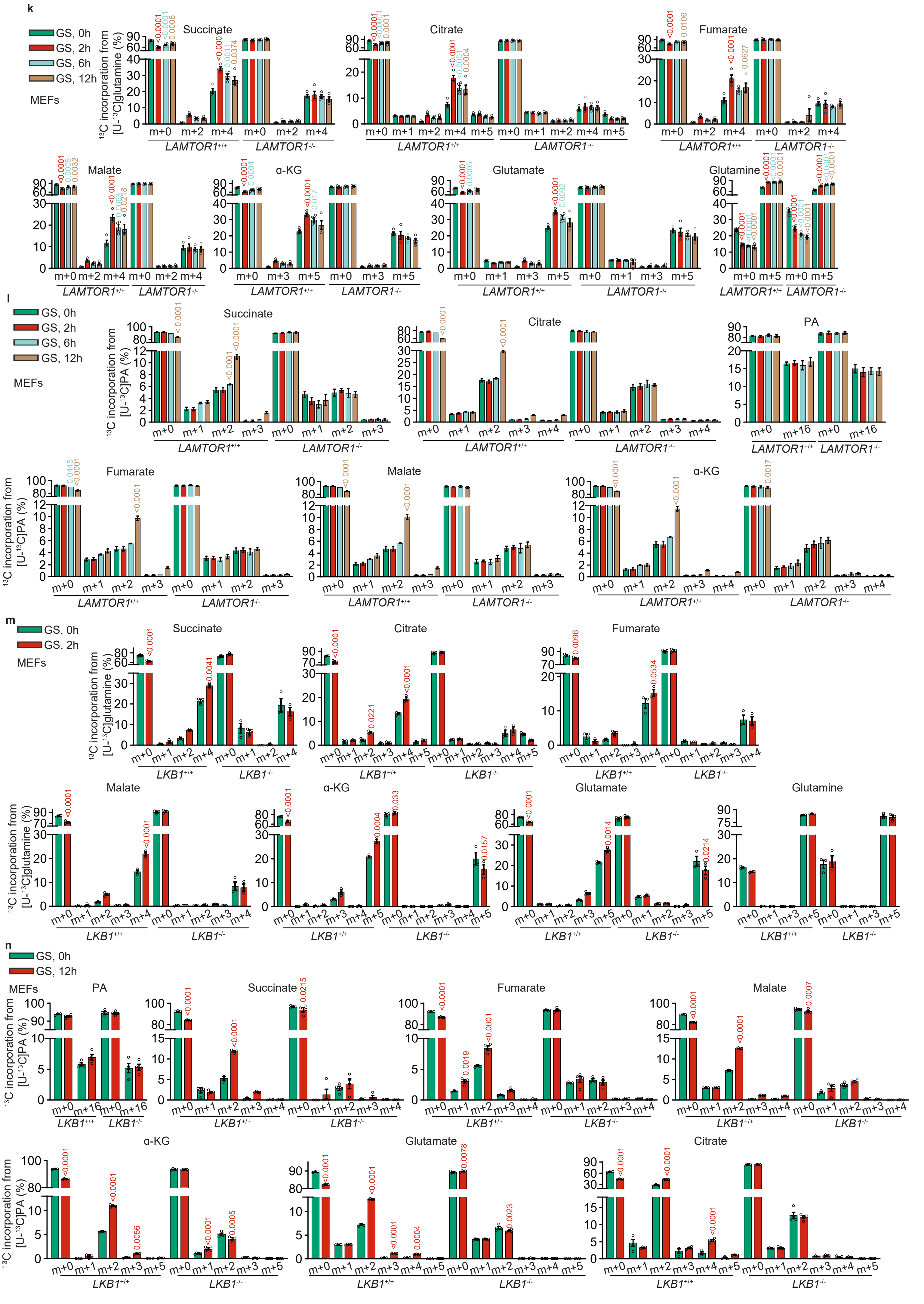

Supplementary information, Fig. S1 (cont.)

o

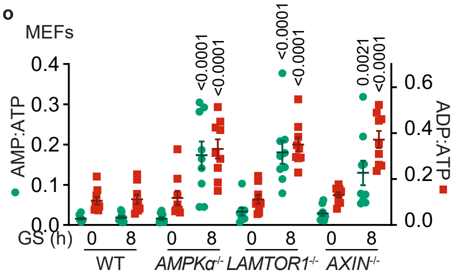

Supplementary information Fig. S1 AMPK promotes the utilization of glutamine

a Glucose starvation leads to a fast and persistent activation of AMPK. MEFs were glucose-starved for desired durations, followed by immunoblotting for p-AMPKα and p-ACC.

b, c, f-j Levels of other isotopomers of the labeled TCA cycle intermediates shown in Fig. 1a (**b**), 1b (**c**), 1d (**f**), 1e (**g**), 1f (**i**), 1g (**h**) and 1h (**j**). Data are shown as mean ± SEM; n = 4 for each condition; p values were determined by one-way ANOVA, followed by Tukey (PA of **c**, **h**, **j**, and malate and glutamine of **i**) or two-way ANOVA, followed by Tukey (**b**, **c** (except PA), **f** (succinate, citrate and glutamine), **g**, **h** (except PA), **i** (except malate and glutamine), **j** (except PA)) or Sidak (fumarate, malate, α-KG and glutamate of **f**).

d Deamination reaction is promoted under glucose starvation. MEFs were glucose starved for 2 h. At 20 min before sample collection, cells were labeled with [alpha-^{15}N]glutamine, followed by determination of the levels of m+1 glutamate (Glu), alanine (Ala), and aspartate (Asp), all indicators to the rates of deamination reactions, along with glutamine (Gln). Data are shown as mean ± SEM; n = 4 for each condition; p values were determined by two-way ANOVA, followed by Tukey.

e Glucose starvation does not promote lipolysis in MEFs. MEFs were glucose starved for 2 h, followed by determining free glycerol in a culture medium to reflect the rates of lipolysis. Data are shown as mean ± SEM; n = 5 biological replicates for each condition; p values were determined by unpaired two-tailed Student's *t*-test.

k-n LAMTOR1 and LKB1 are required for the promotion of glutamine utilization. Experiments were performed as in Fig. 1a (**k**, **m**) and 1b (**l**, **n**), respectively, except that *LAMTOR1*$^{-/-}$ MEFs (**k**, **l**) or *LKB1*$^{-/-}$ MEFs were used. Data are shown as mean ± SEM; n = 4 for each condition; p values were determined by two-way ANOVA, followed by Tukey.

o Ablation of lysosomal AMPK activation that blocks the promotion of both glutaminolysis and FAO in low glucose, caused energy deficiencies. MEFs with *AMPKα*, *AXIN*, or *LAMTOR1* knocked out were glucose-starved for 8 h, followed by determining the AMP:ATP and ADP:ATP ratios by CE-MS. Data are shown as mean ± SEM; n = 9 for each condition; p values were determined by two-way ANOVA, followed by Sidak.

Experiments in this figure were performed three times.
