# Supplementary material for: AMPK targets PDZD8 to trigger carbon source shift from glucose to glutamine
Source: Cell Res. 2024 Jun 19;34(10):683–706. doi: 10.1038/s41422-024-00985-6 (PMC11442470; doi:10.1038/s41422-024-00985-6)
Supplement: Supplementary file 2 — Supplementary information, Fig. S2 [file 41422_2024_985_MOESM2_ESM.pdf]

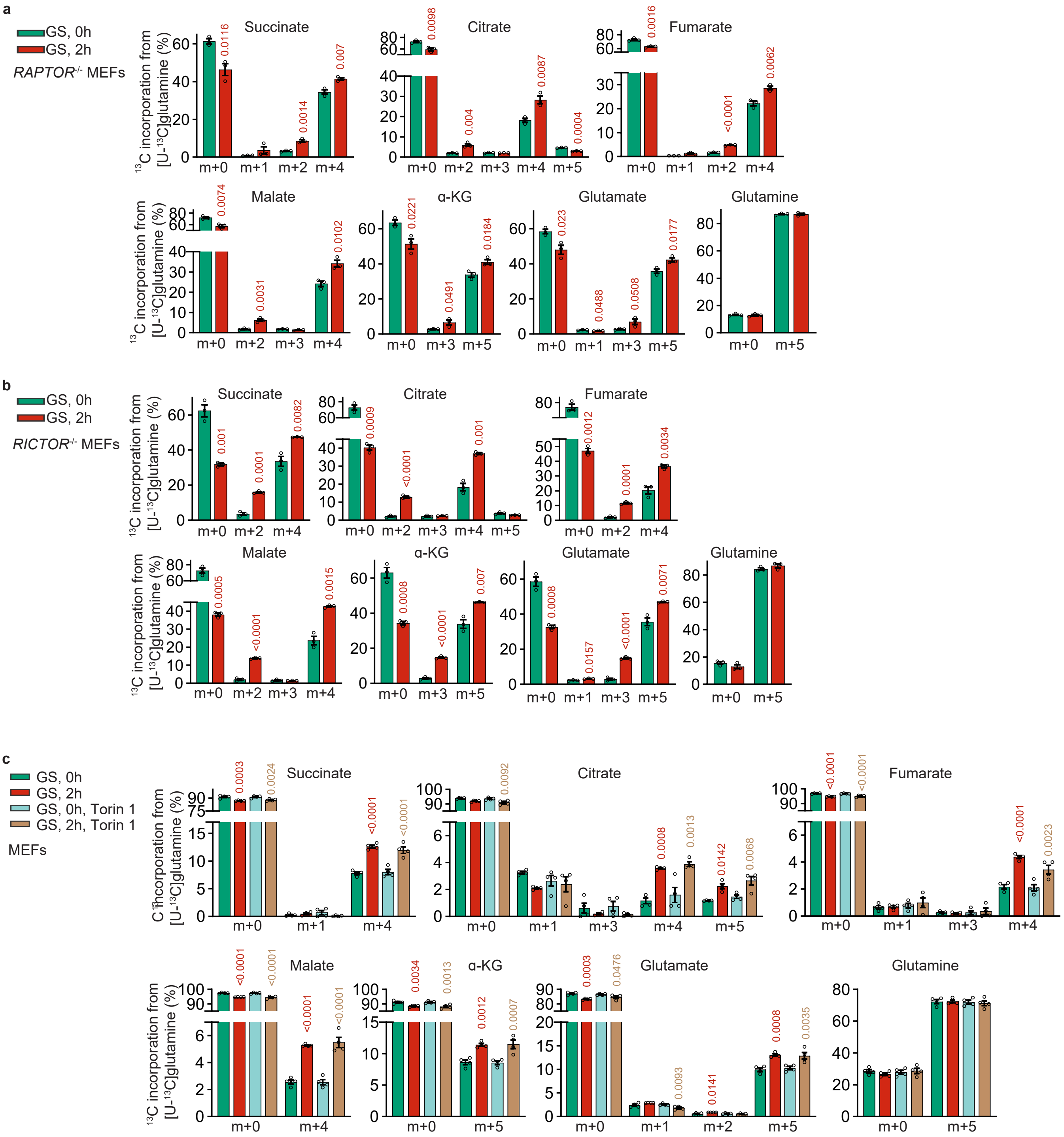

Supplementary information Fig. S2 mTOR is not required for the promotion of glutamine utilization in low glucose

a-c *RAPTOR*$^{-/-}$ MEFs (**a**), *RICTOR*$^{-/-}$ MEFs (**b**) or wildtype MEFs incubated in 10 nM Torin 1 (**c**) were starved for glucose for 2 h, followed by determination of glutamine utilization as in Fig. 1a. Data are shown as mean ± SEM; n = 4 (**c**) or 3 (others) for each condition; p values were determined by two-way ANOVA, followed by Sidak (**c**) or by unpaired two-tailed Student's *t*-test (others).

Experiments in this figure were performed three times.
