# Supplementary material for: AMPK targets PDZD8 to trigger carbon source shift from glucose to glutamine
Source: Cell Res. 2024 Jun 19;34(10):683–706. doi: 10.1038/s41422-024-00985-6 (PMC11442470; doi:10.1038/s41422-024-00985-6)
Supplement: Supplementary file 3 — Supplementary information, Fig. S3 [file 41422_2024_985_MOESM3_ESM.pdf]

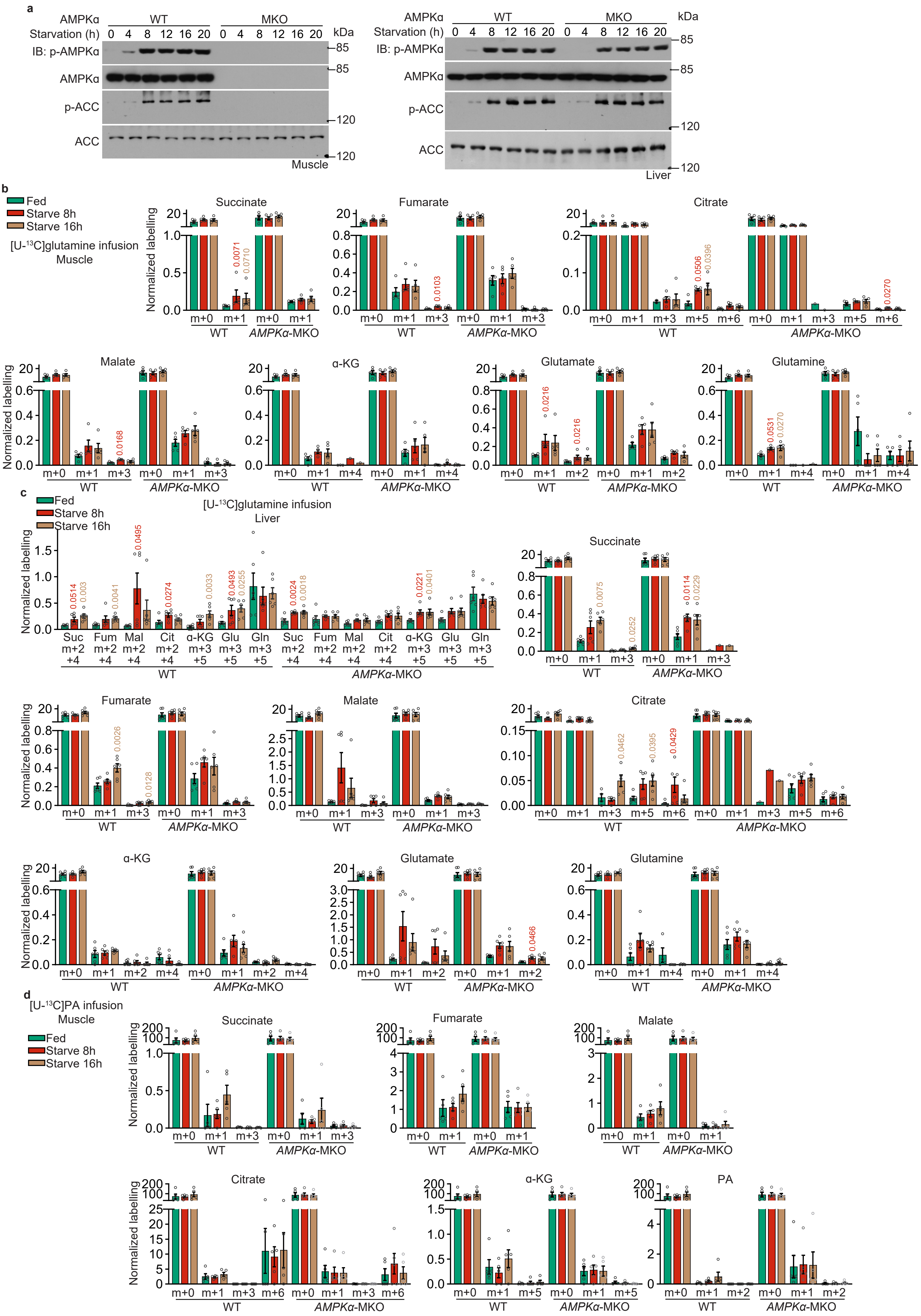

Supplementary information, Fig. S3

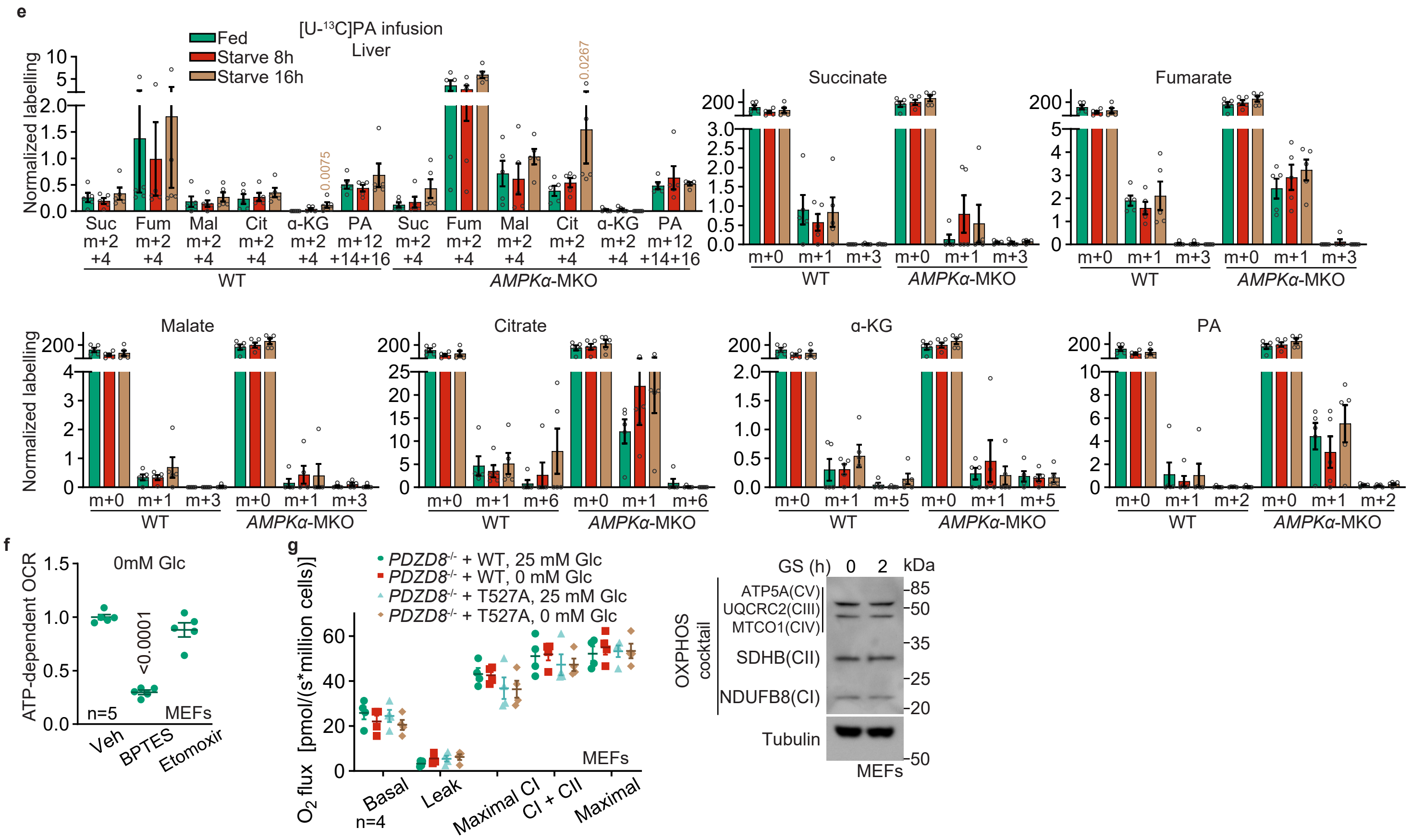

Supplementary information Fig. S3 PDZD8 specifically required for the utilization of glutamine

a Validation of *AMPKα*-MKO mice. *AMPKα*-MKO mice were starved for desired durations, and the muscle (left panel) and liver (right panel) tissues were excised, followed by immunoblotting.

b-e Levels of other isotopomers of the labeled TCA cycle intermediates shown in Fig. 1i (**b**; see also c for the rates of hepatic glutaminolysis, as a control) and 1j (**d**; see also **e** for the rates of hepatic FAO, as a control). Data are shown as mean ± SEM; n = 5 (**b**, **d**, and **e**), or 6 (**c**) biological replicates for each condition. p values in **b** were determined by: a) one-way ANOVA, followed by Dunn: succinate (m+1), citrate (m+0), malate (m+1), glutamate (m+1 and m+2) of WT MEFs, citrate (m+3) of KO MEFs, and α-KG (m+4) and glutamine (m+4) of both WT and KO MEFs; b) one-way ANOVA, followed by Sidak: citrate (m+3) of WT MEFs; and c) one-way ANOVA, followed by Tukey: others. p values in **c** were determined by one-way ANOVA, followed by: a) Dunn: fumarate (m+2+4; means that the sum of m+2 and m+4 isotopomers of fumarate; same hereafter), α-KG (m+2) and glutamine (m+4) of WT mice, and α-KG (m+3+5 and m+4), glutamate (m+3+5), succinate (m+3), fumarate (m+3) and citrate (m+3) of KO mice; b) Sidak: succinate (m+2+4), malate (m+2+4) and α-KG (m+3+5) of WT mice, and glutamine (m+3+5) of KO mice; c) Dunnett: citrate (m+2+4), glutamate (m+3+5) and glutamine (m+3+5) of WT mice, and succinate (m+2+4), fumarate (m+2+4), malate (m+2+4) and citrate (m+2+4) of KO mice; and d) Tukey: others. p values in **d** were determined by one-way ANOVA, followed by: a) Dunn: succinate (m+3) of WT mice, succinate (m+1), malate (m+1) and citrate (m+1) of KO mice; and α-KG (m+5) and PA (m+1 and m+2) of both WT and KO mice; and b) Tukey: others. p values in **e** were determined by one-way ANOVA, followed by: a) Dunn: fumarate (m+2+4), α-KG (m+2+4 and m+5), citrate (m+1), succinate (m+3), fumarate (m+3), malate (m+3) and PA (m+1, m+2 and m+12+14+16) of WT mice, and citrate (m+2+4, m+1 and m+6), fumarate (m+3), malate (m+1) and PA (m+12+14+16) of KO mice; and b) Tukey: others.

f Inhibition of glutaminolysis, but not FAO, prevents OCR increases at early starvation. MEFs were pre-treated with 20 μM BPTES for 10 h, or 10 μM etomoxir for 8 h, and then glucose-starved for 2 h (early starvation), followed by determination of OCR by Seahorse Analyzer. Data are shown as mean ± SEM; n = 5 biological replicates for each condition; p values were determined by one-way ANOVA, followed by Tukey.

g Glucose starvation does not affect the protein contents or the efficiency of the mitochondrial electron transport chain. MEFs were permeabilized with digitonin to expose the electron transport chain, followed by addition of substrate of each mitochondrial respiratory complex to determine its activity (see "Determination of electron transport chain integrity" in Methods section; left panel; data are shown as mean ± SEM; n = 4 for each condition); p values were determined by two-way ANOVA, followed by Tukey. See also the lower panel for the protein levels of each mitochondrial respiratory complex before and after glucose starvation.

Experiments in this figure were performed three times.
