# Supplementary material for: AMPK targets PDZD8 to trigger carbon source shift from glucose to glutamine
Source: Cell Res. 2024 Jun 19;34(10):683–706. doi: 10.1038/s41422-024-00985-6 (PMC11442470; doi:10.1038/s41422-024-00985-6)
Supplement: Supplementary file 4 — Supplementary information, Fig. S4 [file 41422_2024_985_MOESM4_ESM.pdf]

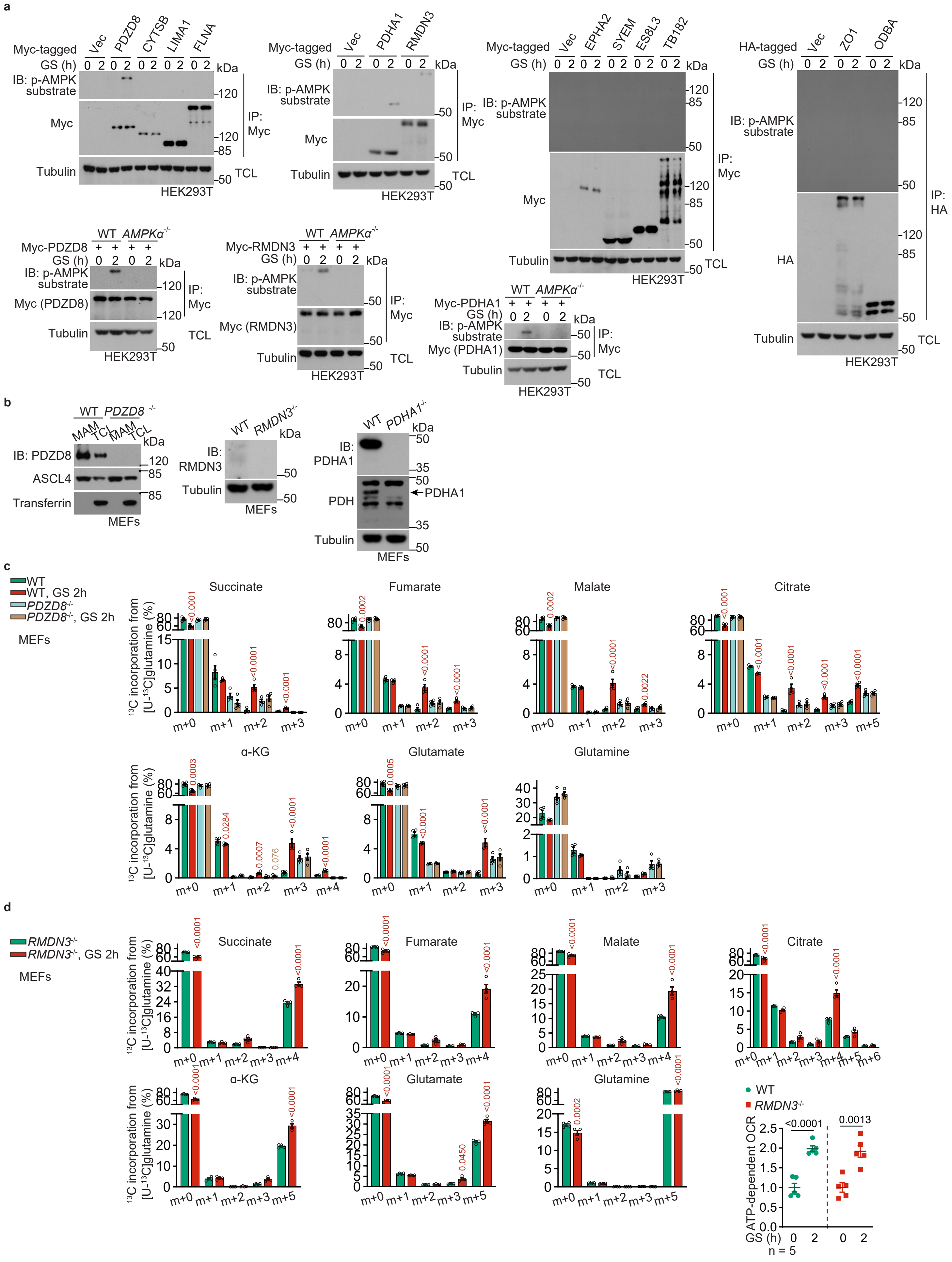

Supplementary information, Fig. S4

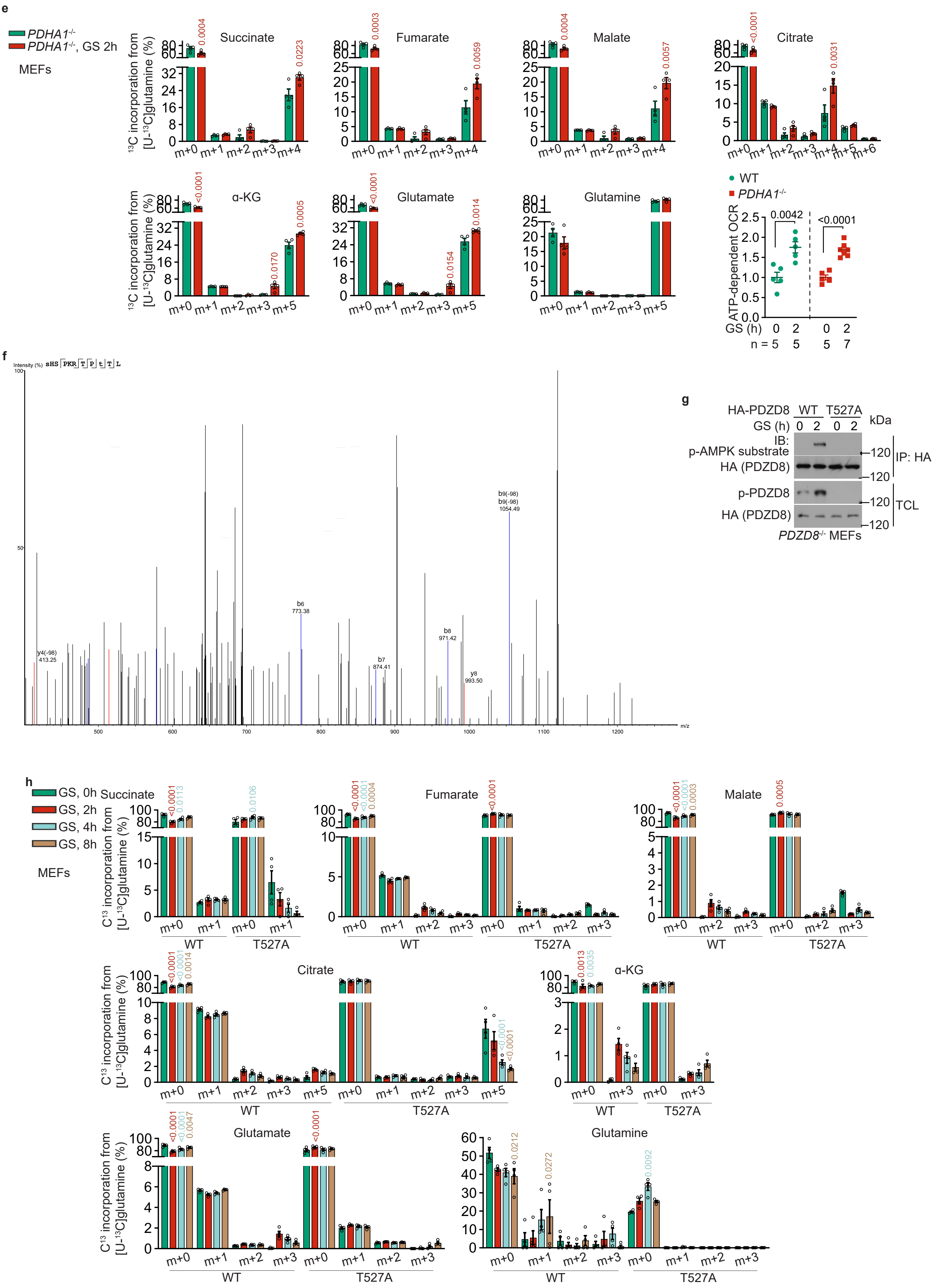

Supplementary information, Fig. S4 (cont.)

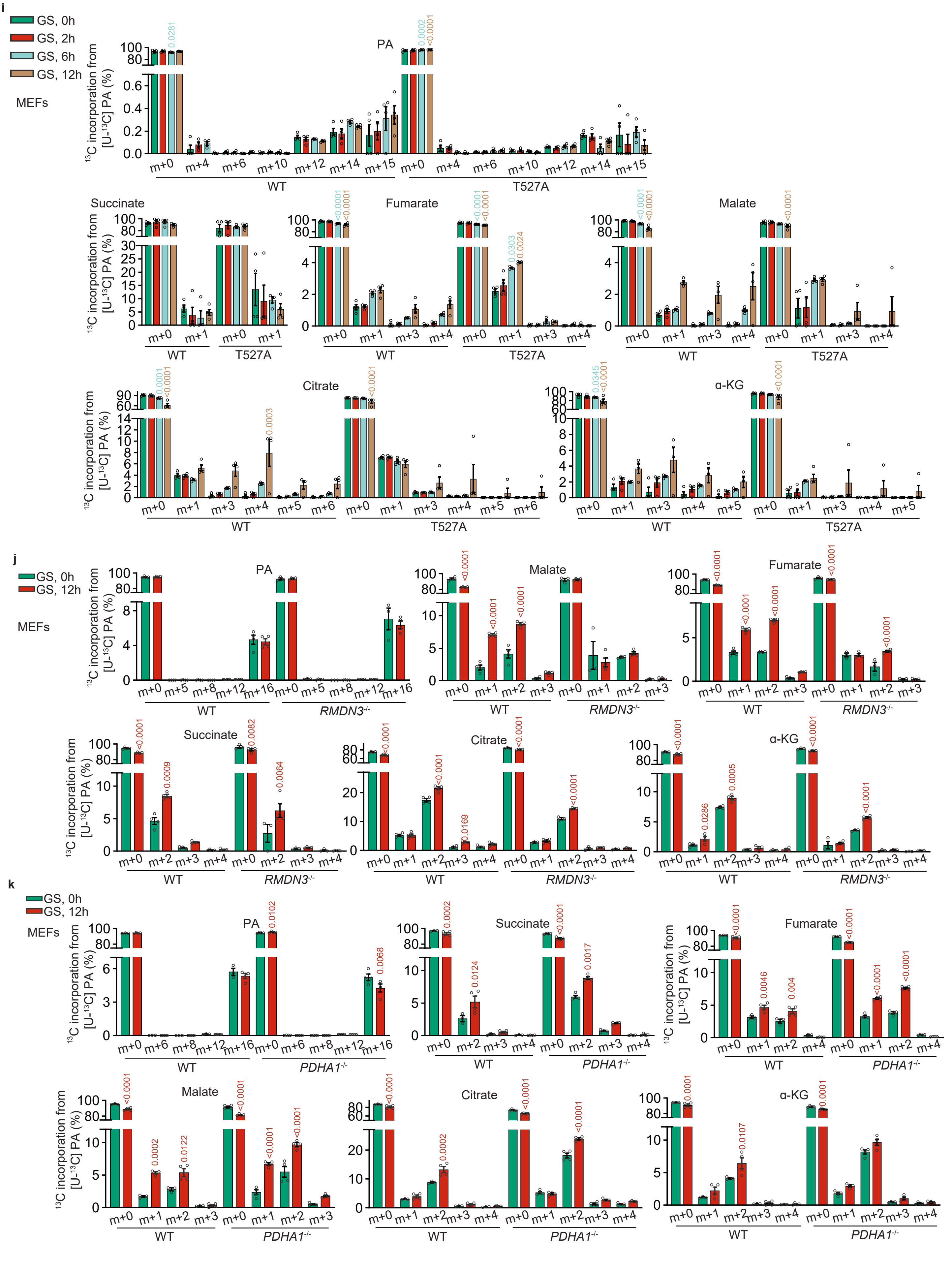

Supplementary information, Fig. S4 (cont.)

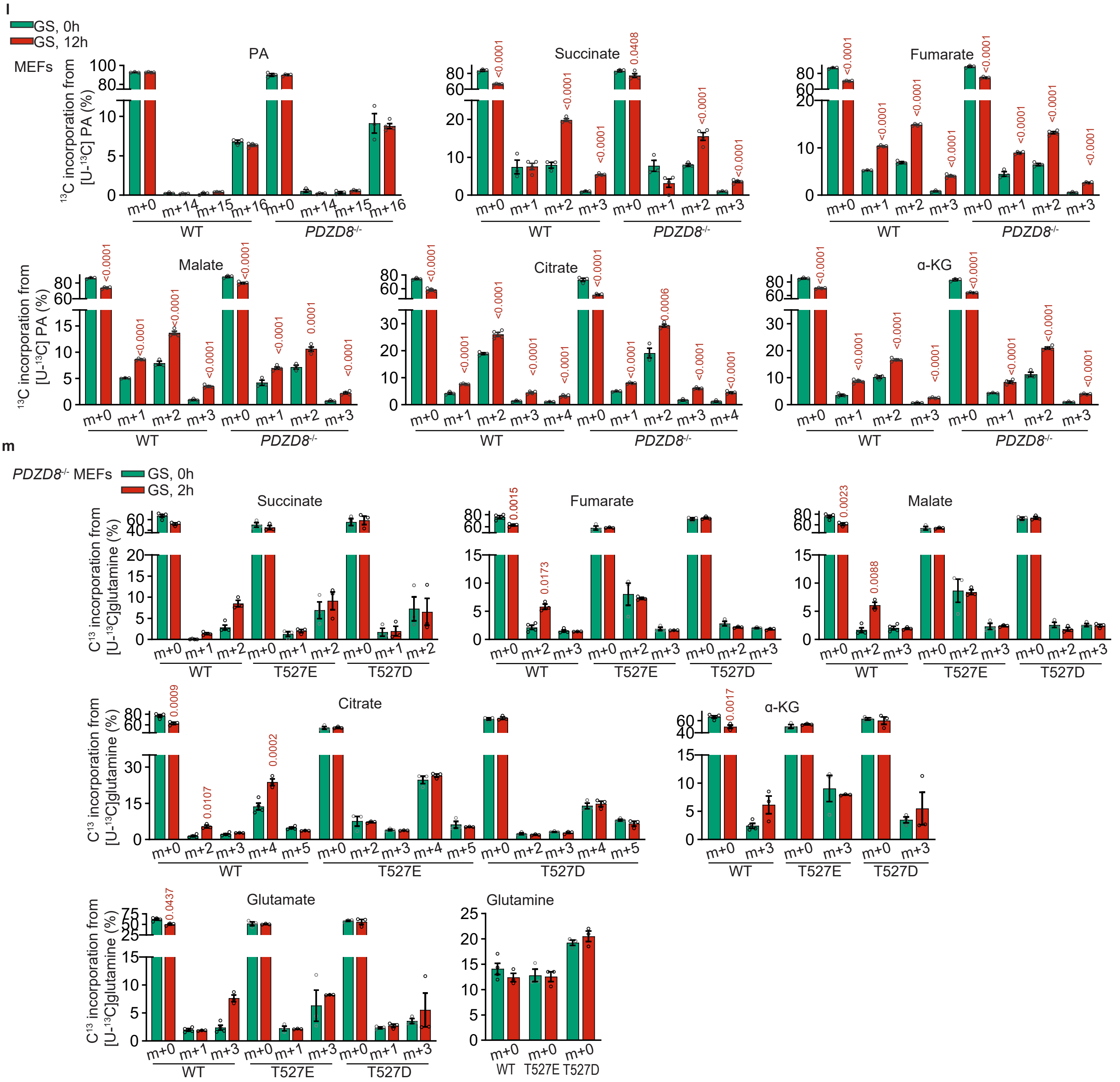

Supplementary information Fig. S4 PDZD8 is a substrate of AMPK

a Verification of possible AMPK substrate(s) in MAM. In the upper panel, HEK293T cells transfected with different constructs of potential AMPK substrates (HA- or Myc-tagged) hit by mass spectrometry (listed in Supplementary information, Table S1) were glucose-starved for 2 h, followed by immunoprecipitation using antibodies against the HA-tag or Myc-tag, and followed by immunoblotting using the antibody for pan-phospho-AMPK-substrates. In the lower panel, wildtype and *AMPKα*$^{-/-}$ HEK293T cells were transfected with Myc-tagged, PDZD8, RMDN3, and PDHA1, three phosphoproteins hit by the mass spectrometry, followed by glucose starvation, immunoprecipitation and immunoblotting as in the upper panel.

b Validation of *PDZD8*, *RMDN3* and *PDHA1* knockout in MEFs. Cells were lysed or subjected to the purification of MAM, followed by immunoblotting.

c PDZD8 is required for the promotion of glutaminolysis in low glucose. Levels of other isotopomers of the labeled TCA cycle intermediates in the upper panel of Fig. 2b are shown. Data are shown as mean ± SEM; n = 4 for each condition; p values were determined by two-way ANOVA, followed by Sidak.

d, e *RMDN3* and *PDHA1* are not responsible for the promotion of glutaminolysis in low glucose. *RMDN3*$^{-/-}$ MEFs (**d**) or *PDHA1*$^{-/-}$ MEFs (**e**) were glucose-starved for 2 h, followed by determination of the glutaminolysis as in Fig. 1a. Data are shown as mean ± SEM; n = 4 for each treatment; p values were determined by two-way ANOVA, followed by Sidak. See also the OCR levels of each cell line in the lower right panel, which are determined through Seahorse Analyzer. Data were normalized to the unstarved group of treatment, and are shown as mean ± SEM; n values represent biological replicates for each condition, and were labeled in each panel; p values were determined by unpaired two-tailed Student's *t*-test.

f Typical spectrogram showing that the T527 site of PDZD8 is phosphorylated.

g Validation of p-T527-PDZD8 antibody. *PDZD8*$^{-/-}$ MEFs stably expressing HA-tagged PDZD8 or PDZD8-T527A were glucose-starved for 2 h, followed by immunoblotting using the p-T527-PDZD8 antibody. As a control, HA-tagged PDZD8 was immunoprecipitated, followed by immunoblotting using the pan-phospho-AMPK-substrates antibody.

h, i Levels of other isotopomers of the labeled TCA cycle intermediates shown in Fig. 2h (**h**), 2i (**i**). Data are shown as mean ± SEM; n = 4 biological replicates for each condition; p values were determined by two-way ANOVA, followed by Tukey, all compared to the unstarved group of each genotype.

j-l RMDN3, PDHA, and PDZD8 are not required for the low glucose-induced FAO. *RMDN3*$^{-/-}$ MEFs (**j**), *PDHA1*$^{-/-}$ MEFs (**k**), or *PDZD8*$^{-/-}$ MEFs (**l**) were glucose-starved for 2 h, followed by determination of FAO as in Fig. 1b. Data are shown as mean ± SEM; n = 3 (unstarved group of *RMDN3*$^{-/-}$ MEFs in **j**, unstarved group of WT MEFs in **k**, and unstarved group of WT and *PDZD8*$^{-/-}$ MEFs in **l**) or 4 (others) for each treatment; p values were determined by two-way ANOVA, followed by Sidak (**l**) or Tukey (others).

m Levels of other isotopomers of the labeled TCA cycle intermediates shown in Fig. 2j. Data are shown as mean ± SEM; n = 4 (unstarved group of WT) or 3 (others) for each condition; p values were determined by one-way ANOVA, followed by Sidak.

Experiments in this figure were performed three times.
