# Supplementary material for: AMPK targets PDZD8 to trigger carbon source shift from glucose to glutamine
Source: Cell Res. 2024 Jun 19;34(10):683–706. doi: 10.1038/s41422-024-00985-6 (PMC11442470; doi:10.1038/s41422-024-00985-6)
Supplement: Supplementary file 5 — Supplementary information, Fig. S5 [file 41422_2024_985_MOESM5_ESM.pdf]

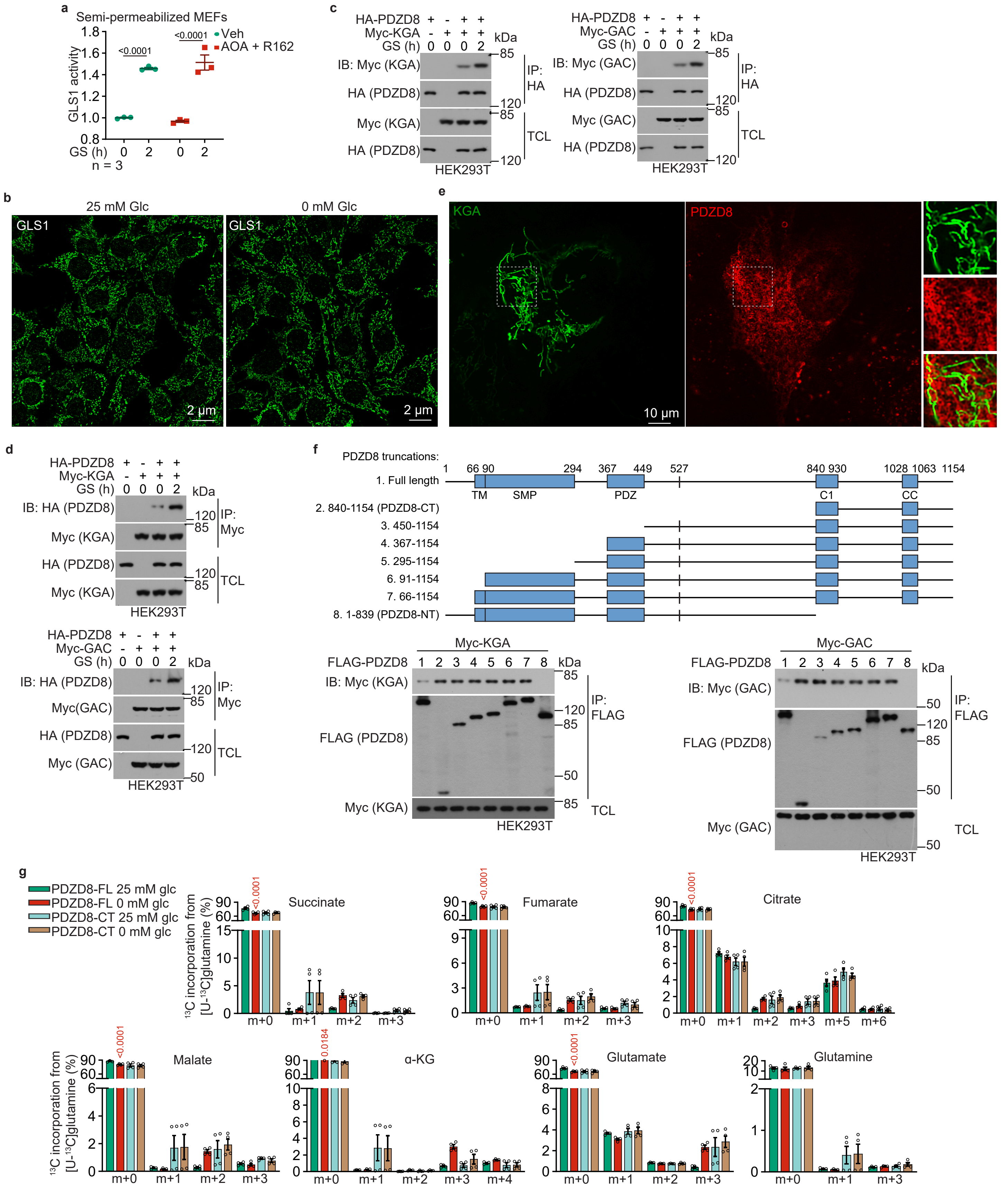

Supplementary information, Fig. S5

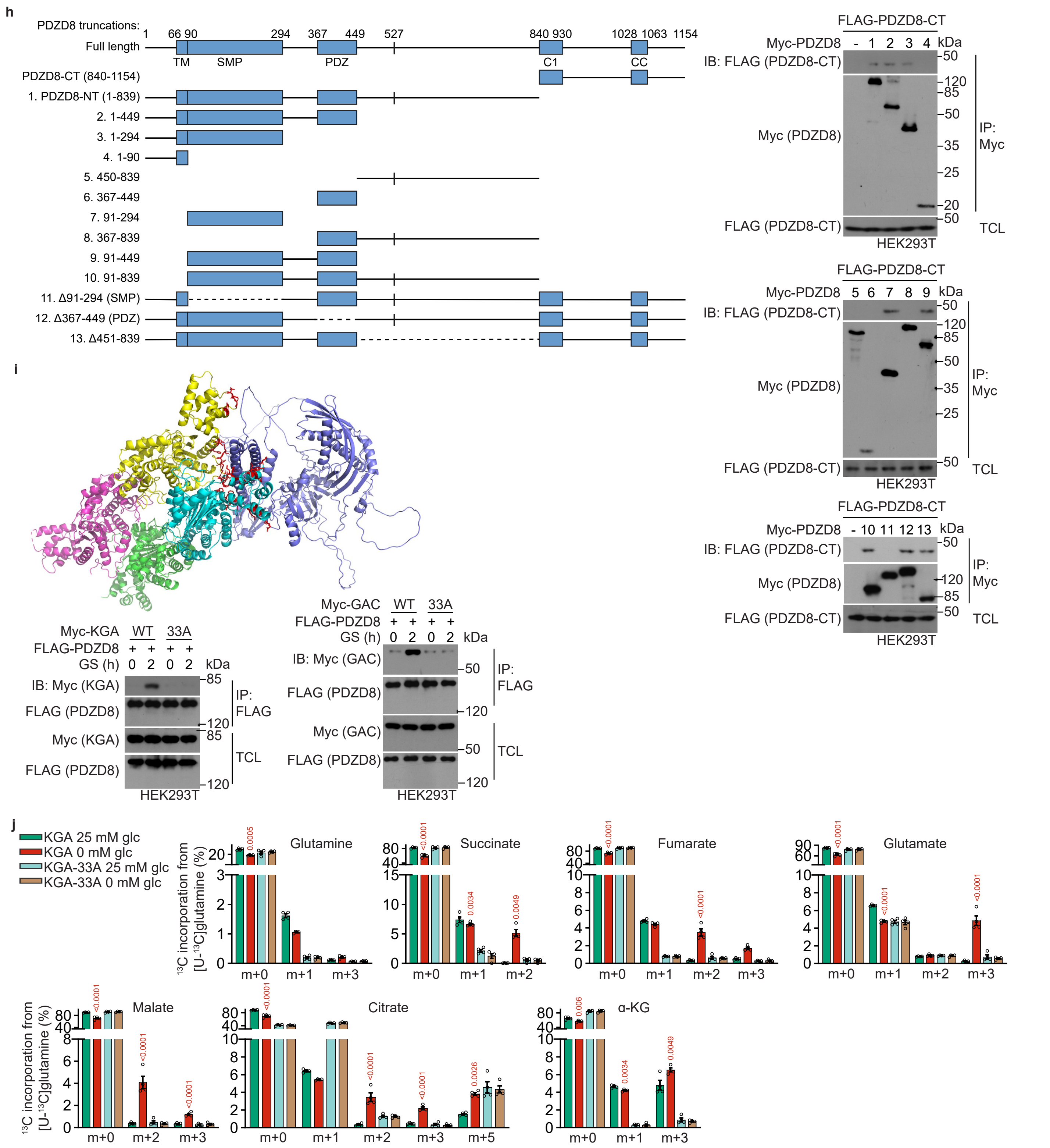

Supplementary information Fig. S5 PDZD8 promotes GLS1 activity through interacting with GLS1

a Validation data of Fig. 3a, b, indicating that the conversion of glutamate to α-ketoglutarate, catalyzed by GLUD1 and transaminases, is almost undetectable in the semi-permeabilized system. Experiments were performed as in Fig. 3a, b, except that 20 μM AOA (a pan-transaminase inhibitor) and 20 μM R162 (an inhibitor to GLUD1) were included in the system. Data are shown as mean ± SD; n = 3 biological replicates for each condition; p values were determined by two-way ANOVA, followed by Sidak.

b Glucose starvation does not cause GLS1 filamentation. MEFs were starved for glucose for 2 h, followed by determining the filamentation of GLS1 by immunofluorescent staining.

c, d Ectopically expressed PDZD8 and GLS1 interact with each other. HEK293T cells were transfected with different combinations of PDZD8 and GLS1 (GAC or KGA), and then glucose-starved for 2 h, followed by immunoprecipitation and immunoblotting.

e PDZD8 is juxtaposed with GLS1 in cells. MEFs stably expressing FLAG-tagged KGA and Myc-tagged PDZD8 were stained and subjected to SIM imaging.

f Domain mapping for the region on PDZD8 responsible for interacting with GLS1. Myc-tagged KGA (left panel) or GAC (right panel) was co-transfected with FLAG-tagged PDZD8 or deletion mutants into HEK293T cells. Immunoprecipitation was performed using an antibody against the FLAG tag, followed by immunoblotting.

g Levels of other isotopomers of the labeled TCA cycle intermediates shown in Fig. 4c. Data are shown as mean ± SEM; n = 4 biological replicates for each condition; p values were determined by two-way ANOVA, followed by Tukey.

h PDZD8-NT interacts with PDZD8-CT. Various Myc-tagged PDZD8 deletion mutants were co-transfected with FLAG-tagged PDZD8-CT into HEK293T cells. Immunoprecipitation was performed using an anti-Myc antibody, followed by immunoblotting.

i In silico modeling of PDZD8 (blue) bound to GAC (as a tetramer, colored in magenta, yellow, green and cyan each). The interface is shown as stick structures, and is colored in red. See the detailed list of the 33 residues of GLS1 involved in the interface in "Determination of GLS1-PDZD8 interface" in the Methods section. See also the right panel for the validation of the GLS1-PDZD8 interface, in which HEK293T cells were transfected with FLAG-tagged PDZD8 and Myc-tagged KGA or GAC. Immunoprecipitation was then performed using anti-FLAG antibody, followed by immunoblotting.

j Levels of other isotopomers of the labeled TCA cycle intermediates shown in Fig. 4i. Data are shown as mean ± SEM; n = 4 for each condition; p values were determined by two-way ANOVA, followed by Sidak.

Experiments in this figure were performed three times.
