# Supplementary material for: AMPK targets PDZD8 to trigger carbon source shift from glucose to glutamine
Source: Cell Res. 2024 Jun 19;34(10):683–706. doi: 10.1038/s41422-024-00985-6 (PMC11442470; doi:10.1038/s41422-024-00985-6)
Supplement: Supplementary file 6 — Supplementary information, Fig. S6 [file 41422_2024_985_MOESM6_ESM.pdf]

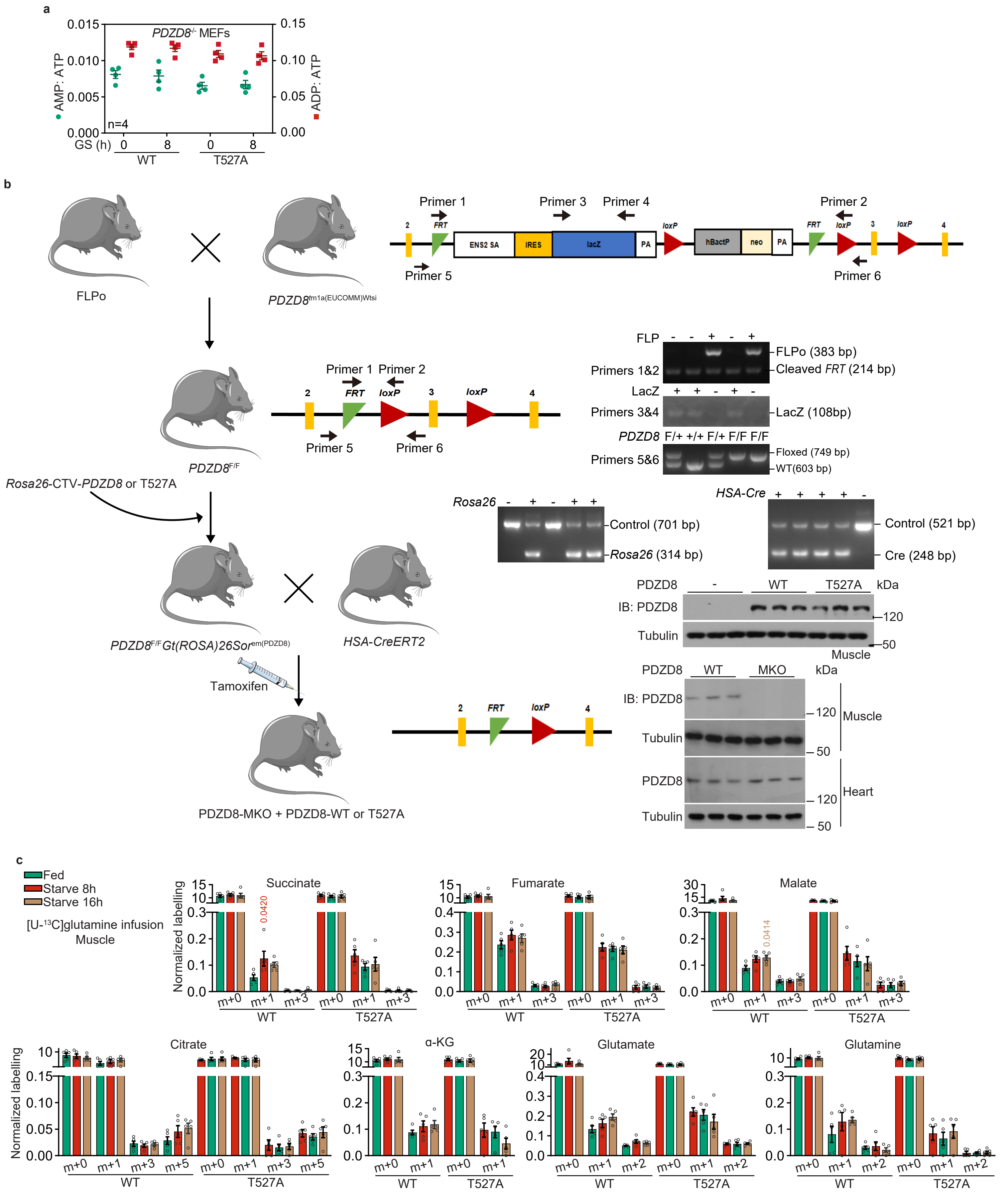

Supplementary information, Fig. S6

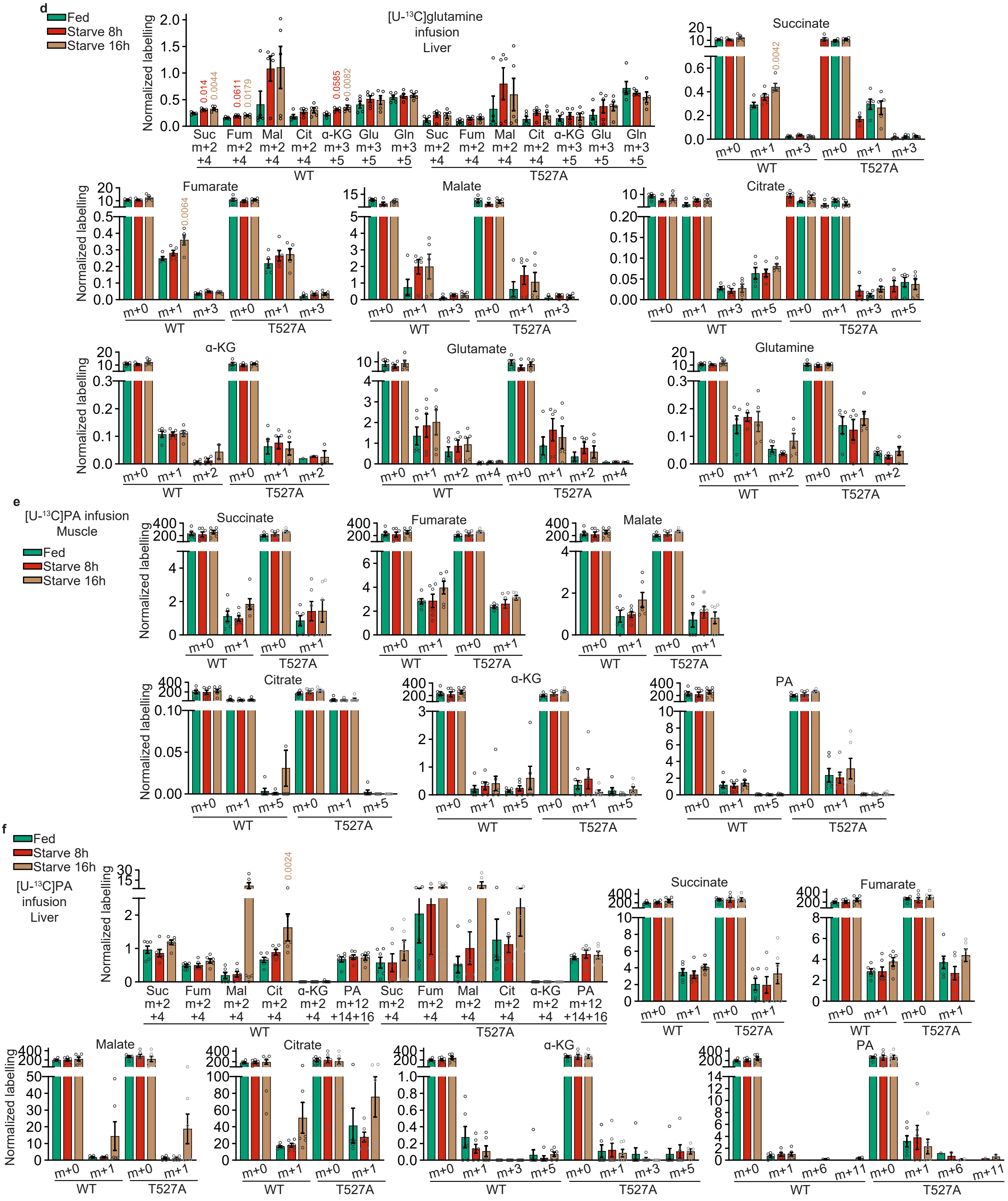

Supplementary information, Fig. S6 (cont.)

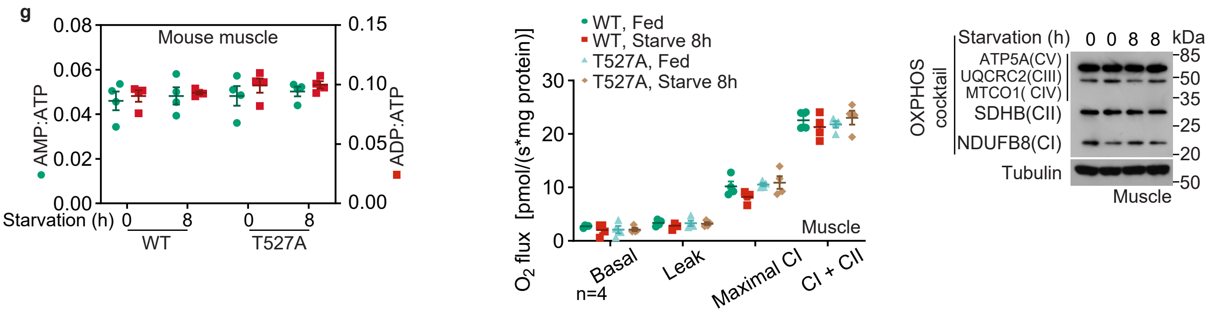

Supplementary information Fig. S6 PDZD8 depends on AMPK to promote glutaminolysis in muscle tissues

a, g Enhanced glutaminolysis is not required for the maintainance of energy levels in low glucose. The *PDZD8*$^{-/-}$ MEFs with wildtype PDZD8 or PDZD8-T527A re-introduction (**a**), or the mice with muscular PDZD8 replaced with wildtype PDZD8 or PDZD8-T527A (**g**), were glucose-starved for 8 h (**a**) or fasted for 8 h (**g**), followed by determining the AMP:ATP and ADP:ATP ratios by CE-MS (**g**, left) and the efficiency of the mitochondrial electron transport chain (**g**, right; as in Supplementary information Fig. S3g). Data are shown as mean ± SEM; n = 4 for each condition; p values were determined by one-way ANOVA, followed by Tukey (**a**, left panel of **g**) or two-way ANOVA, followed by Tukey (**g**, right).

b Validation of mice with muscular PDZD8 replaced with wildtype PDZD8 or PDZD8-T527A. The *PDZD8*$^{F/F}$ mice, generated through breeding the *PDZD8*-KO-first mice (*Pdzd8*$^{tm1a(EUCOMM)Wtsi}$) with the FLPo mice (to remove the FRT-flanked Stop element ahead of the *PDZD8* locus), were validated through: a) determining FRT cleavage (the "cleaved FRT" band; genotyped through using Primers #1 and #2); and b) determining the LacZ (of the Stop element) removal (genotyped through using Primers #3 and #4). See also the genotyping results for determining the existence of FLPo. The floxed PDZD8 was then validated through genotyping using Primers #5 and #6. After introducing the PDZD8 and PDZD8-T527A into the *PDZD8*$^{F/F}$ mice through the *Rosa26*-LSL system (see "Mouse strains" of Methods section; validated by genotyping the *ROSA26* sequence), mice were bred with *HSA-CreERT2* mice (validated by genotyping the *HSA-Cre* sequence). The muscle-specific expression of PDZD8 was then induced by tamoxifen injection, followed by validation through immunoblotting. See also primer sequences and PCR programs used for genotyping in the "Mouse strains" of the Methods section.

c-f Levels of other isotopomers of the labeled TCA cycle intermediates shown in Fig. 5a (**c**; see also **d** for the rates of hepatic glutaminolysis), 5b (**e**; see also **f** for the rates of hepatic FAO). Data are shown as mean ± SEM; n = 5 (**c, d**), or 6 (**e, f**) biological replicates for each condition. p values were determined by one-way ANOVA, followed by a) Dunn: malate (m+0) and glutamate (m+0) of WT mice in **c**, citrate (m+0 and m+1) of T527A mice in **c**, glutamate (m+2 and m+4) of WT mice in **d**, α-KG (m+2) of T527A mice in **d**, PA (m+1), α-KG (m+5) and citrate (m+1 and m+5) of WT mice in **e**, citrate (m+1 and m+5) and α-KG (m+5) of T527A mice in **e**, citrate (m+1 and m+2+4), malate (m+1 and m+2+4), α-KG (m+2+4) and PA (m+6 and m+11) of WT mice in **f**, and malate (m+1 and m+2+4), citrate (m+2+4), α-KG (m+2+4) and PA (m+6 and m+11) of T527A mice in **f**; b) Sidak: citrate (m+3 and m+5) of T527A mice in **c**, succinate (m+2+4), fumarate (m+2+4), malate (m+2+4), glutamate (m+3+5), glutamine (m+3+5) and α-KG (m+3+5) of WT mice in **d**, succinate (m+2+4), fumarate (m+2+4), malate (m+2+4), glutamate (m+3+5) and α-KG (m+3+5) of T527A mice in **d**, succinate (m+2+4), fumarate (m+2+4) and PA (m+12+14+16) of WT mice in **f**, and fumarate (m+2+4) of T527A mice in **f**; and c) Tukey: for others.

Experiments in this figure were performed three times.
