# Supplementary material for: AMPK targets PDZD8 to trigger carbon source shift from glucose to glutamine
Source: Cell Res. 2024 Jun 19;34(10):683–706. doi: 10.1038/s41422-024-00985-6 (PMC11442470; doi:10.1038/s41422-024-00985-6)
Supplement: Supplementary file 7 — Supplementary information, Fig. S7 [file 41422_2024_985_MOESM7_ESM.pdf]

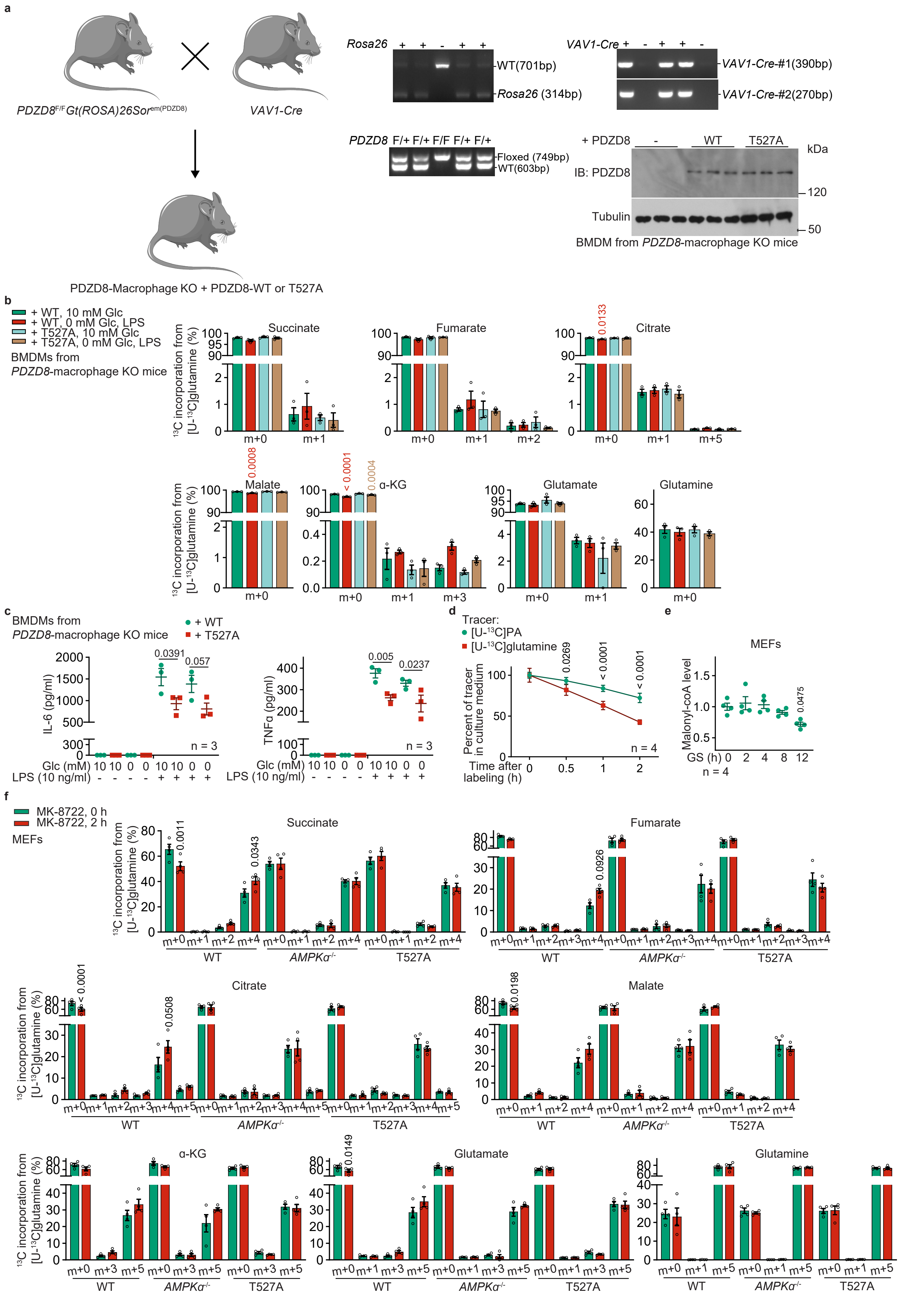

Supplementary information, Fig. S7

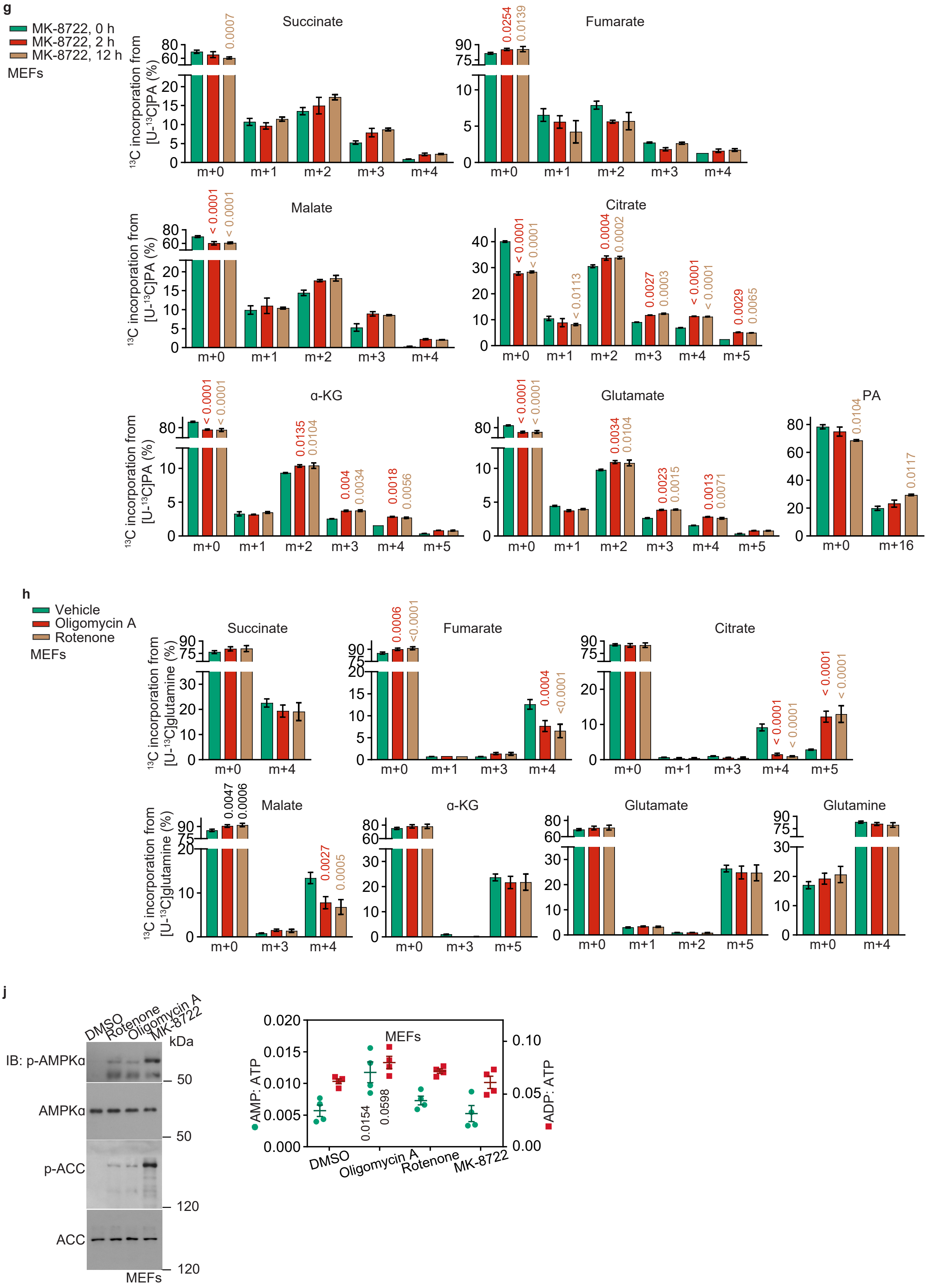

Supplementary information, Fig. S7 (cont.)

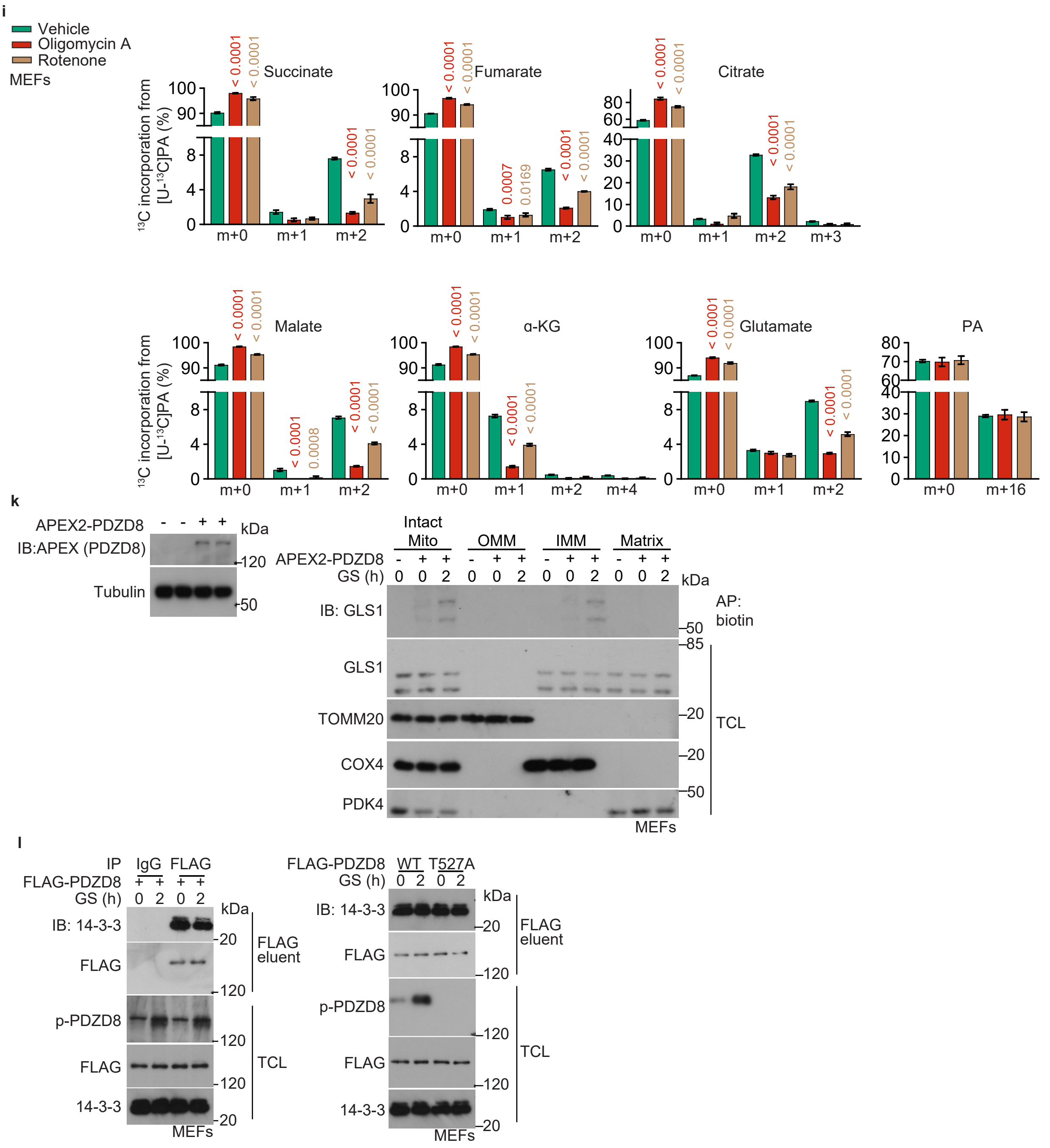

Supplementary information Fig. S7 PDZD8, depending on phosphorylation by AMPK, is required for the promotion of glutaminolysis in macrophages

a Validation of mice with macrophagic PDZD8 replaced with wildtype PDZD8 or PDZD8-T527A. Mice were generated as in Supplementary information, Fig. S6b, except that the *VAV1-Cre* mice were used to cross with the *PDZD8*$^{F/F}$ mice carrying *Rosa26*-LSL-PDZD8 or *Rosa26*-LSL-PDZD8-T527A. Representative genotyping results are shown on the left panel, and the validation results of PDZD8 expression (in isolated BMDMs), by immunoblotting, are shown on the left panel.

b Levels of other isotopomers of the labeled TCA cycle intermediates shown in Fig. 5e. Data are shown as mean ± SEM; n = 3 for each condition; p values were determined by one-way ANOVA, followed by Tukey (malate and glutamine); two-way ANOVA, followed by Tukey (others).

c PDZD8 is required for the secretion of pro-inflammatory cytokines in macrophages in vitro. BMDMs isolated from mice with macrophagic PDZD8 replaced with wildtype PDZD8 or PDZD8-T527A, were incubated in RPMI 1640 containing 10 mM or 0 mM glucose and 10 ng/mL LPS for 6 h. Cells were then lysed, followed by determining levels of TNFα and IL-6. Data are shown as mean ± SEM; n = 3 for each condition, and p values were determined by two-way ANOVA, followed by Tukey.

d The uptake of palmitic acid by MEFs is slower than glutamine. Cells were labeled with [U-^{13}C]-glutamine or [U-^{13}C]-PA, respectively, followed by determining the concentrations of each tracer in the culture medium at indicated time points. Data are shown as mean ± SEM; n = 4 samples for each condition; p values were determined by two-way ANOVA, followed by Sidak.

e Levels of malonyl-CoA in MEFs decrease only after prolonged glucose starvation. MEFs were glucose-starved at the desired time, followed by determining the levels of malonyl-CoA through HPLC-MS. Data are shown as mean ± SEM; n = 4 for each condition; p values were determined by one-way ANOVA, followed by Tukey.

f-j Effects of allosteric AMPK activators on glutaminolysis and FAO promotion. MEFs were treated with 10 μM MK-8722 (**f**, **g**), 0.2 μM oligomycin A, or 0.2 μM rotenone (**h**, **i**), followed by determining the rates of glutaminolysis as in Fig. 1a (**f**, **h**) and FAO as in Fig. 1b (**g**, **i**). Data are shown as mean ± SEM; n = 3 (**g**) or 4 (others) for each condition; p values were determined by two-way ANOVA, followed by Tukey. See also validation data on AMPK activation, as well as AMP:ATP and ADP:ATP ratios (mean ± SEM; n = 4 for each condition; p values were determined by one-way ANOVA, followed by Tukey) in **j**.

k PDZD8 interacts with GLS1 located on the external side of IMM. MEFs stably expressing PDZD8-APEX2 (induced by incubating with doxycycline at a final concentration of 100 ng/mL for 24 h; see validation data in the left panel) were treated with biotinyl tyramide and hydrogen peroxide, followed by purification of OMM, IMM, and matrix. The affinity pulldown (AP) of biotinylated proteins was then performed using Streptavidin Magnetic Beads, followed by immunoblotting.

l PDZD8 interacts with 14-3-3. MEFs with FLAG-tagged PDZD8 or PDZD8-T527A stably expressed were glucose-starved for 2 h, followed by immunoprecipitation of FLAG, and then eluted with FLAG peptide. The immunoprecipitates were then subjected to immunoblotting to determine the levels of co-immunoprecipitated 14-3-3.

Experiments in this figure were performed three times.
