# Supplementary material for: AMPK targets PDZD8 to trigger carbon source shift from glucose to glutamine
Source: Cell Res. 2024 Jun 19;34(10):683–706. doi: 10.1038/s41422-024-00985-6 (PMC11442470; doi:10.1038/s41422-024-00985-6)
Supplement: Supplementary file 8 — Supplementary information notes [file 41422_2024_985_MOESM8_ESM.docx]

**Supplementary information** **notes**

**Note S1**

We conclude that under glucose starvation, the rates of glutamine utilization through glutaminolysis were promoted based on the following two lines of evidence:

a) In a dynamic labeling assay in which MEFs were pre-treated with [U-^13^C]glutamine for 20 min, a time duration lay in a phase during which the ^13^C incorporation in the pool of TCA cycle intermediates was increased in a time-dependent manner (not saturated; see “Determination of rates of glutaminolysis and FAO” in the Methods section), the levels of ^13^C-isotopologs of TCA cycle intermediates were increased (Fig. 1a), indicating the overall rates of glutamine carbon entry into the TCA cycle were increased.

b) The deamination reaction, which reflects the conversion of glutamine to glutamate and then α-KG catalyzed separately by GLS1, glutamic-pyruvic transaminase 2 (GPT2), and glutamic-oxaloacetic transaminase 2 (GOT2), as determined by the levels of ^15^N-labeled alanine and aspartate (both are m+1) in MEFs pre-treated with [alpha-^15^N]glutamine, was significantly promoted in low glucose (Supplementary information, Fig. S1d). Furthermore, knockdown of *GLS1* or treatment of GLS1 inhibitor BPTES blocked the effects of enhanced glutaminolysis on OCR (Fig. 1o; Supplementary information, Fig. S3f). These data indicate that the channeling of glutamine to the TCA cycle through glutaminolysis is promoted in low glucose.

Data shown in Fig. 1a and Supplementary information, Fig. S1b also indicate an elevated, glutamine-derived cataplerosis from the TCA cycle in low glucose, including an elevated reductive carboxylation (determined by the levels of m+5 and m+3 citrate), an elevated citrate-pyruvate cycle (determined by the levels of m+3 malate), and an elevated malate-aspartate shuttle (determined by the levels of m+4 aspartate). The cataplerosis-mediated dissipation of the TCA cycle intermediates prevents the accumulation of anions in the mitochondrial matrix brought about by the increased glutaminolysis, which may inhibit TCA reactions (reviewed in ref. ^1^), thereby sustaining the high rates of TCA reactions observed in Fig. 1a.

We found that FAO was promoted much later than glutaminolysis in low glucose, as determined by levels of [U-^13^C]palmitate-labeled, ^13^C-isotopologs of TCA cycle intermediates during the starvation periods. One may argue that it is the utilization of stored (unlabeled) TAG first, and labeled palmitate next, in low glucose, that may lead to the delayed elevation of ^13^C-isotopologs of TCA cycle intermediates. We therefore determined the levels of free glycerol in the culture medium to reflect the rates of lipolysis in MEFs and found that glucose starvation did not elevate free glycerol contents (Supplementary information, Fig. S1e), ruling out the possibility that stored TAG utilization leads to the delayed promotion of FAO in low glucose. Consistently, we have shown that knockout of *CPT1* or treatment of CPT1 inhibitor etomoxir in low glucose did not block the promotion of glutaminolysis and OCR (Fig. 1p; Supplementary information, Fig. S3f).

**Note S2**

To determine the contributions of glutamine and palmitate to fuel the TCA in low glucose, we separately labeled MEFs with [U-^13^C]glutamine or [U-^13^C]palmitate until isotopic enrichment has reached steady states (been saturated, see “Determination of rates of glutaminolysis and fatty acid oxidation (FAO)” of the Methods section, and ref. ^2^ for glutamine labeling, and ref. ^3^ for PA labeling), and then determined the contribution of these carbon sources to the pool of TCA cycle intermediates. As shown in Fig. 1c, in high glucose, the contributions of glutamine and palmitate to succinate or malate were approximately 45% and 10%, respectively. Under 2-h glucose starvation, the contribution of glutamine to the pool of TCA cycle intermediates was increased, while palmitate increased until 12 h of glucose starvation. The abundance of TCA cycle intermediates, along with glutamine and PA (pool size of each metabolite), remained unchanged before and after glucose starvation.

**Note S3**

As described previously^4-7^, a typical AMPK substrate motif meets at least one of the following properties:

a) A hydrophobic residue (valine, isoleucine, leucine, methionine, phenylalanine, tryptophan and cysteine) in -5 position relative to the phosphoacceptor site (serine or threonine), and the following sites on PDZD8 meet this criteria: S10, T18, T135, S144, T153, T159, T171, S215, S223, T234, S269, T284, T300, S338, S352, S354, S386, T427, S503, S530, T662, S666, S672, S733, T746, T749, T790, S801, S822, S867, S889, T912, S925, S943, T961, S989, S1056, T1103, S1106, S1132 and S1153. We also included those residues show less hydrophobic properties at -5 position (alanine, tyrosine, histidine, threonine, serine, proline and glycine): S15, T79, T81, T86, T91, T94, T101, T120, T239, S244, T268, T326, S331, T348, S353, S403, T425, S426, S476, T489, S491, S496, T527, S538, S558, T569, S570, S631, S663, S673, T678, T696, S699, S747, T767, T769, S775, T837, S842, T894, S927, T932, S957, T971, S980, S991, S996, S1011, T1029, S1080, S1108, S1113, S1137, S1142, and S1144.

b) A basic residue (arginine, lysine, histidine) in -4 position, and sites T120, T234, T239, S244, T284, T326, S363, S386, T427, S497, T527, T528, S663, T696, S699, T767, S801, T807, T863, T901, T941, S957, T974, S996, T997, T1029, and S1074 conform to this criterion.

c) A basic residue in -3 position, and sites T268, S269, T284, T300, T319, T326, S331, S338, T348, S352, S353, S354, S362, S363, T366, S376, T380 and S386 conform to this criterion.

d) A hydrophobic residue in +4 position, and sites T425, S426, T427, S452, S471, S472, S473, S476, T486, T489, S491, S497, S503, S517, S519, S521, T527, T528, S530, S538, S549, S558, T569, S570, T573, S579, T582, S585, S603, S631, T662, S663, T665, S666, T669, S672, S673, T678, S681, S682, T694, T696, S699, S733, T746, S747, T749, S753, S761, T767, T769, S775, T790, S801, T807, S822, T837, S842, T846, T852, T863, S867, T888, S889, T894, T901, T912, S925, S927, T931, T932, T935, T941, S942, S943, S952, T954, S957, T961, S967, T971, T974, S975, S980, T982, S989, S991, S996, T997, S1011, T1029, S1056, T1064, T1065, T1067, S1071, S1074, S1080, T1088, and T1103 conform to this criterion.

Among these predicted sites, S10, S15, T18, T81, T101, T120, T135, S144, T153, T159, T171, S215, S223, T234, T239, S244, T319, T326, S331, S338, T348, S352, S362, S363, T366, S376, T380, S386, T425, S426, T427, S496, S519, S521, T527, T528, S530, S538, S549, S558, S579, S585, T696, S733, S747, S753, S761, T767, T769, S775, T790, S822, S842, T894, T901, S927, T931, T935, T941, S942, S943, S952, T954, S967, S996, S1011, T1029, S1056, T1064, S1071, S1074, S1080, T1088, T1103, S1108, S1113, S1132 and S1144 were hit in mass spectrometry analysis (Supplementary information, Table 1; all converted to human PDZD8 amino acid positions) and were evolutionarily conserved in human. After individually mutating these sites and the other predicted sites as well, we found that T527 is the site of PDZD8 that is phosphorylated by AMPK (Fig. 2c, d). T527 also fits the AMPK substrate motif refined by a very recent study^8^, in which a hydrophobic residue resides in the -2 position, and a proline residue in -1.

**Note S4**

As shown in Supplementary information, Fig. S5f, the truncate PDZD8-CT lacking the N-terminal region showed a significantly higher affinity towards GLS1 than that of full-length PDZD8, indicative of an intramolecular autoinhibition of the C-terminus of PDZD8 by the N-terminus for interacting with GLS1. Indeed, the truncate protein PDZD8-NT showed a strong interaction with PDZD8-CT regardless of glucose concentrations (Supplementary information, Fig. S5h). We also found that phosphorylation of full-length PDZD8 by AMPK led to an increased affinity towards GLS1, to a similar extent to that between PDZD8-CT and GLS1 (Fig. 4e), suggesting that phosphorylation of T527 removes the intramolecular autoinhibition. The FRET-FLIM experiment in live cells also indicated that the N-terminus of PDZD8 was moved away from its C-terminus under glucose starvation, as the fluorescent lifetimes of GFP fused to the C-terminus of PDZD8 were significantly increased due to the removal of FRET brought about by RFP fused to PDZD8-NT (Fig. 4f); knockout of *AMPKα*, or re-introduction of PDZD8-T527A mutant into *PDZD8*^-/-^ cells abolished the low glucose-induced conformational change of PDZD8 (Fig. 4f). Together, we stand to reason that upon phosphorylation at T527, the C-terminal region of PDZD8 is no longer inhibited by the N-terminus, and exhibits stronger affinity for GLS1, which consequentially promotes GLS1 activity.

**References** **for** **SI** **notes**

1 Owen, O. E., Kalhan, S. C. & Hanson, R. W. The key role of anaplerosis and cataplerosis for citric acid cycle function. *The Journal of biological chemistry* **277**, 30409-30412, doi:10.1074/jbc.R200006200 (2002).

2 Wiechert, W. & de Graaf, A. A. In vivo stationary flux analysis by 13C labeling experiments. *Adv Biochem Eng Biotechnol* **54**, 109-154, doi:10.1007/BFb0102334 (1996).

3 Vacanti, N. M. *et al.* Regulation of substrate utilization by the mitochondrial pyruvate carrier. *Molecular cell* **56**, 425-435, doi:10.1016/j.molcel.2014.09.024 (2014).

4 Weekes, J., Ball, K. L., Caudwell, F. B. & Hardie, D. G. Specificity determinants for the AMP-activated protein kinase and its plant homologue analysed using synthetic peptides. *FEBS letters* **334**, 335-339, doi:10.1016/0014-5793(93)80706-z (1993).

5 Dale, S., Wilson, W. A., Edelman, A. M. & Hardie, D. G. Similar substrate recognition motifs for mammalian AMP-activated protein kinase, higher plant HMG-CoA reductase kinase-A, yeast SNF1, and mammalian calmodulin-dependent protein kinase I. *FEBS letters* **361**, 191-195 (1995).

6 Scott, J. W., Norman, D. G., Hawley, S. A., Kontogiannis, L. & Hardie, D. G. Protein kinase substrate recognition studied using the recombinant catalytic domain of AMP-activated protein kinase and a model substrate. *J Mol Biol* **317**, 309-323, doi:10.1006/jmbi.2001.5316 (2002).

7 Gwinn, D. M. *et al.* AMPK phosphorylation of raptor mediates a metabolic checkpoint. *Mol. Cell* **30**, 214-226 (2008).

8 Johnson, J. L. *et al.* An atlas of substrate specificities for the human serine/threonine kinome. *Nature*, doi:10.1038/s41586-022-05575-3 (2023).
