# Supplementary material for: AMPK targets PDZD8 to trigger carbon source shift from glucose to glutamine
Source: Cell Res. 2024 Jun 19;34(10):683–706. doi: 10.1038/s41422-024-00985-6 (PMC11442470; doi:10.1038/s41422-024-00985-6)
Supplement: Supplementary file 11 — Supplementary information, Table S3 [file 41422_2024_985_MOESM11_ESM.pdf]

Supplementary information, Table S3. Summary of lifespan analysis in mice[a,b]

Genotypes/ treatments	Mean life span (hours)			Median life span (hours)						
		95% confidence interval			95% confidence interval					P-value Vs saline control within each genotype (Mantel-CoX)
	Estimated life span ± s.e.m.	Lower bound	Upper bound	Estimated life span ± s.e.m.	Lower bound	Upper bound	N[c]	N[d]	N[e]	
				**Fig. 5h**						
PDZD8-Macrophage KO + WT	57.417 ± 5.391	46.850	67.984	36.000 ± 1.386	33.284	38.716	36	12	48	N/A
PDZD8-Macrophage KO + T527A	73.429 ± 6.375	60.934	85.923	50.000 ± 17.262	16.166	83.834	24	18	42	0.066
				**Fig. 5j (left)**						
Saline	57.034 ± 5.364	46.522	67.547	34.000 ± 2.283	29.525	38.475	42	16	58	N/A
BPTES	72.762 ± 5.432	62.116	83.408	48.000 ± 12.883	22.749	73.251	32	28	60	0.028
				**Fig. 5j (right)**						
Saline	59.804 ± 5.776	48.482	71.126	36.000 ± 1.777	32.518	39.482	35	16	51	N/A
Compound 968	74.043 ± 5.815	62.647	85.440	58.000 ± 6.776	44.719	71.281	28	18	46	0.093
				**Fig. 5k**						
Saline	47.366 ± 6.475	34.676	60.056	24.000 ± 1.052	21.939	26.061	31	10	41	N/A
BPTES	56.244 ± 6.843	42.832	69.656	30.000 ± 3.832	22.490	37.510	28	13	41	0.065
Aldometanib + BPTES	69.783 ± 6.307	57.422	82.144	48.000 ± 9.009	30.343	65.657	27	19	46	0.002

[a]Independent repeats of each lifespan experiment were performed. Data from representative experiments are shown.
[b]Lifespan data sets within each panel of this table were done in parallel and statistical analyses was done within the data set.
[c]Number of mice scored (death events).
[d]Number of mice censored.
[e]Total number of mice.
