# Supplementary material for: AMPK targets PDZD8 to trigger carbon source shift from glucose to glutamine
Source: Cell Res. 2024 Jun 19;34(10):683–706. doi: 10.1038/s41422-024-00985-6 (PMC11442470; doi:10.1038/s41422-024-00985-6)

Full scans.

Blots, as shown in this figure, were cut into slices before incubation with primary antibodies. The Pierce™ Prestained Protein MW Marker, Cat. 26612, from ThermoFisher Scientific, was used as the protein marker.

Fig. 1p

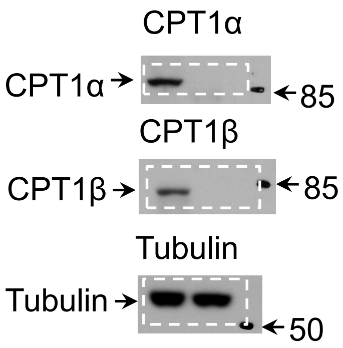

MEFs

Fig. 2a left

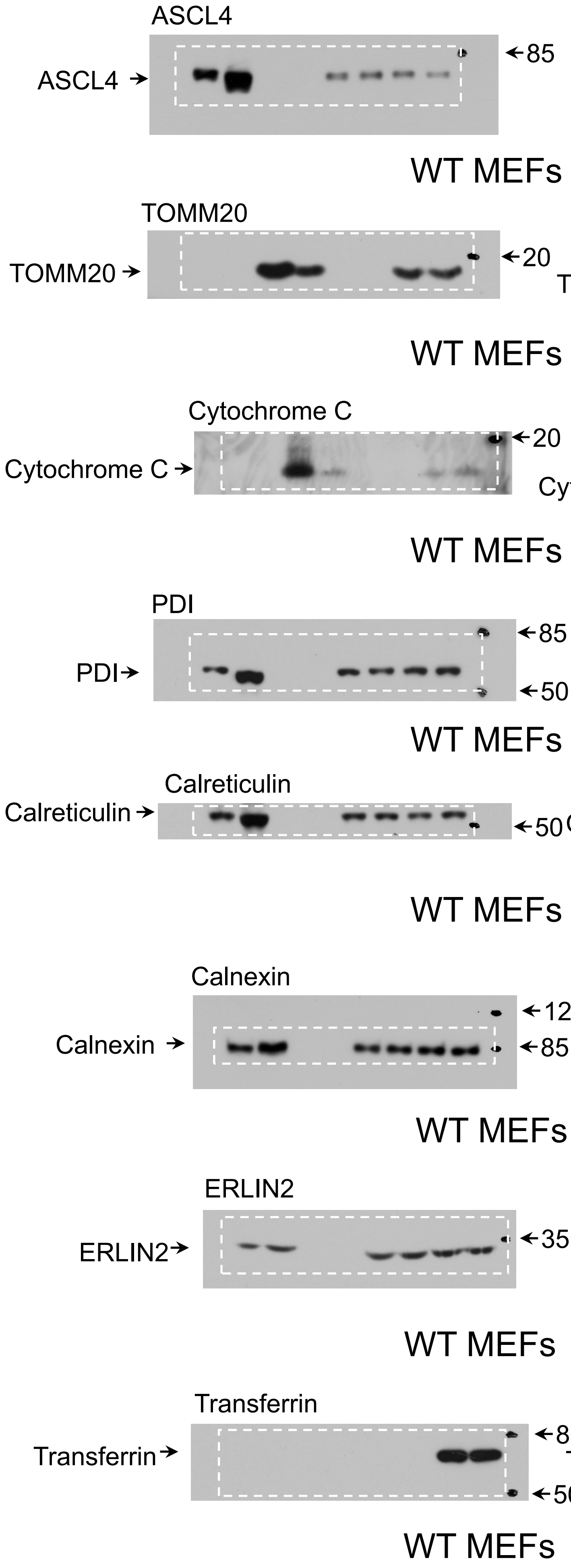

Fig. 2a right

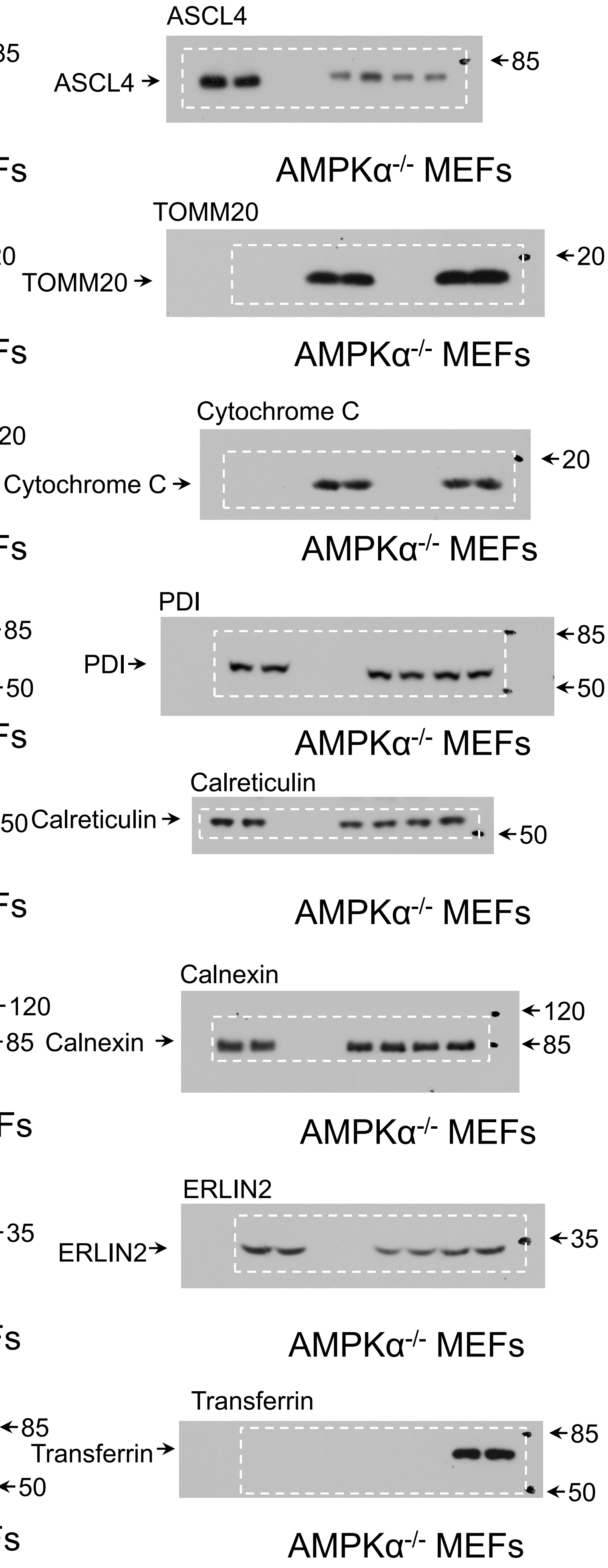

Fig. 2c

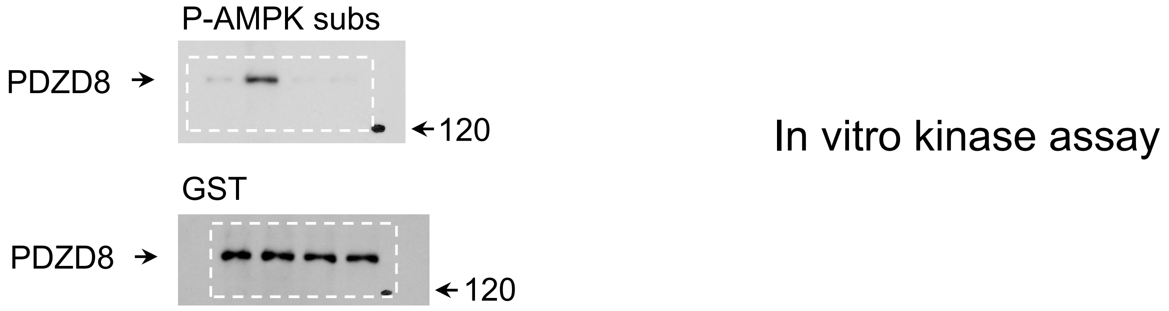

Fig. 2d

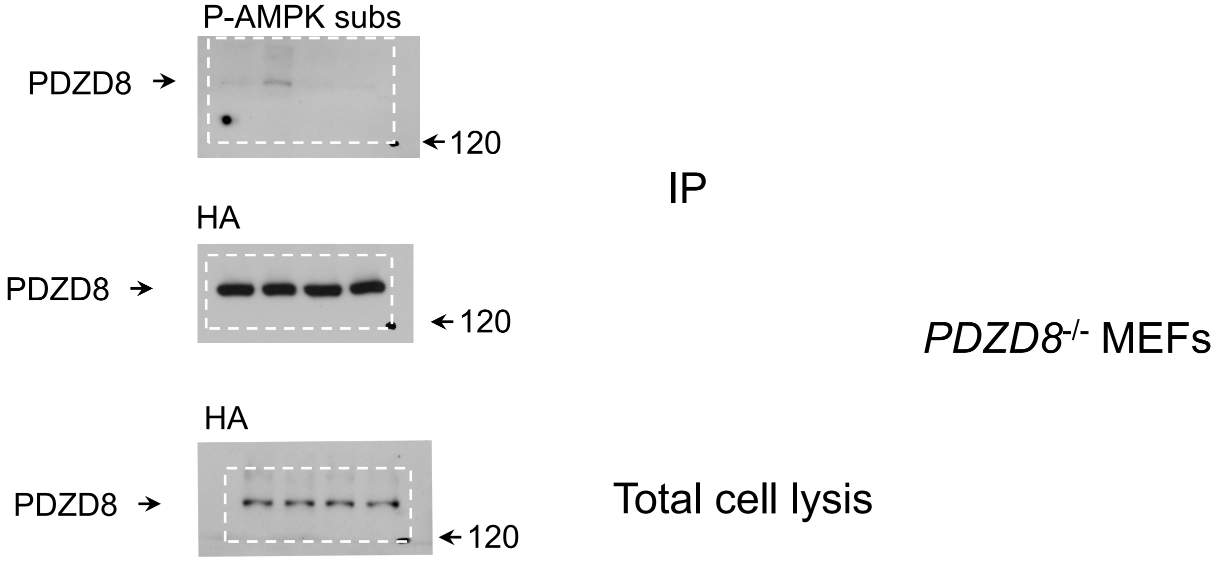

Fig. 2e

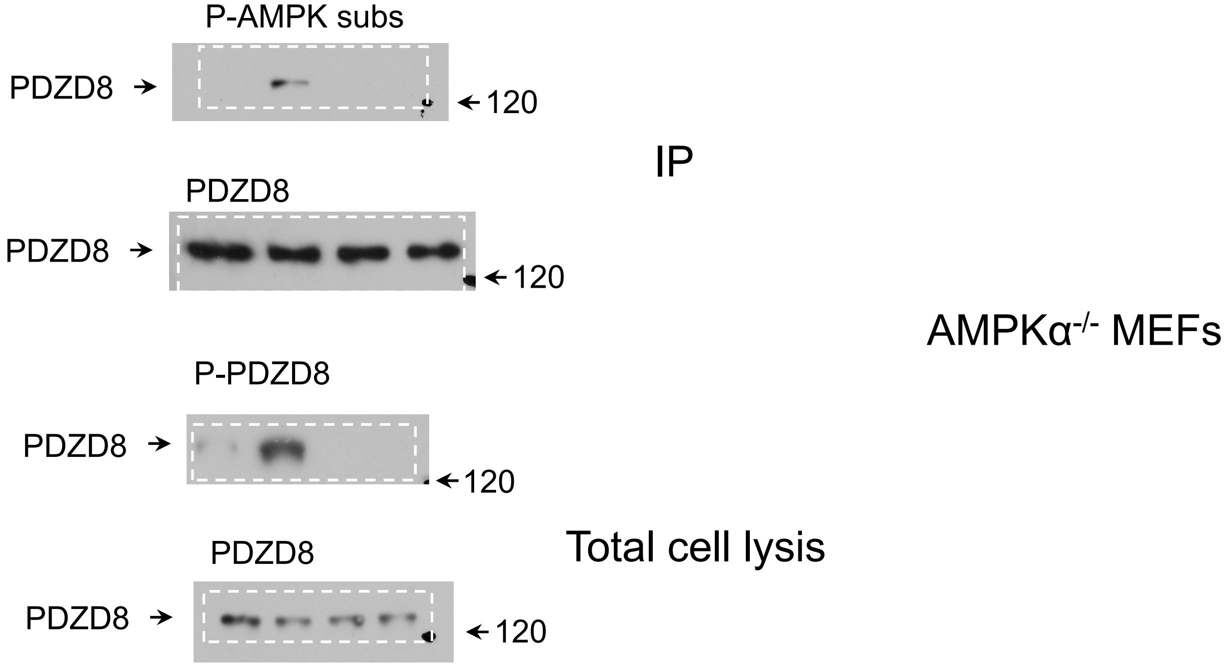

Fig. 2f

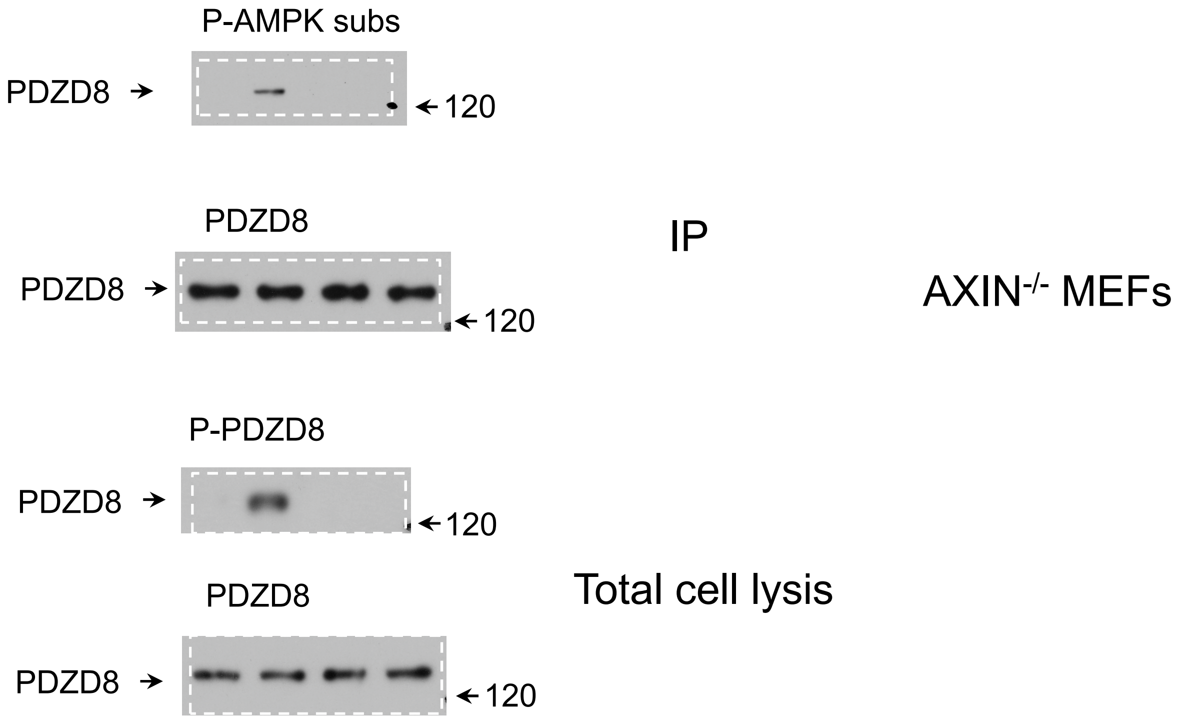
P-AMPK subs
PDZD8
120
PDZD8
IP
AXIN-/- MEFs
PDZD8
120
P-PDZD8
PDZD8
120
Total cell lysis
PDZD8
PDZD8
120

Fig. 2g

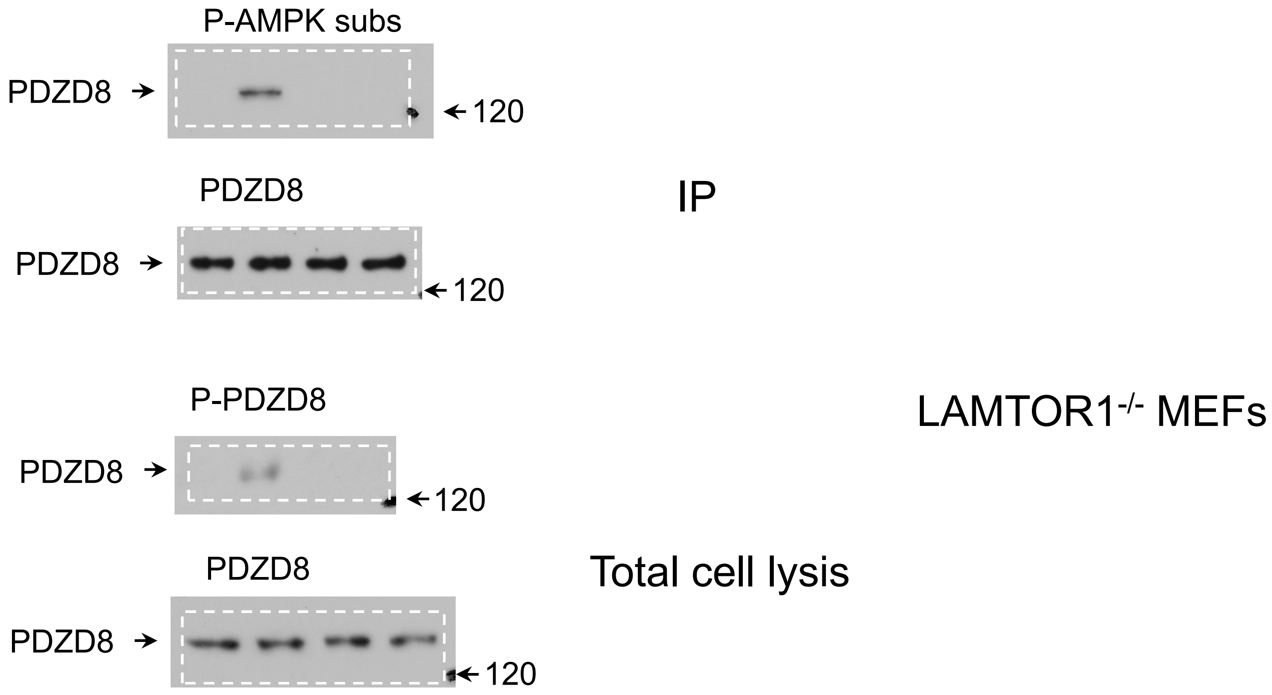
P-AMPK subs
PDZD8
120
PDZD8
IP
PDZD8
120
P-PDZD8
LAMTOR1-/- MEFs
PDZD8
120
PDZD8
Total cell lysis
PDZD8
120

Fig. 3J

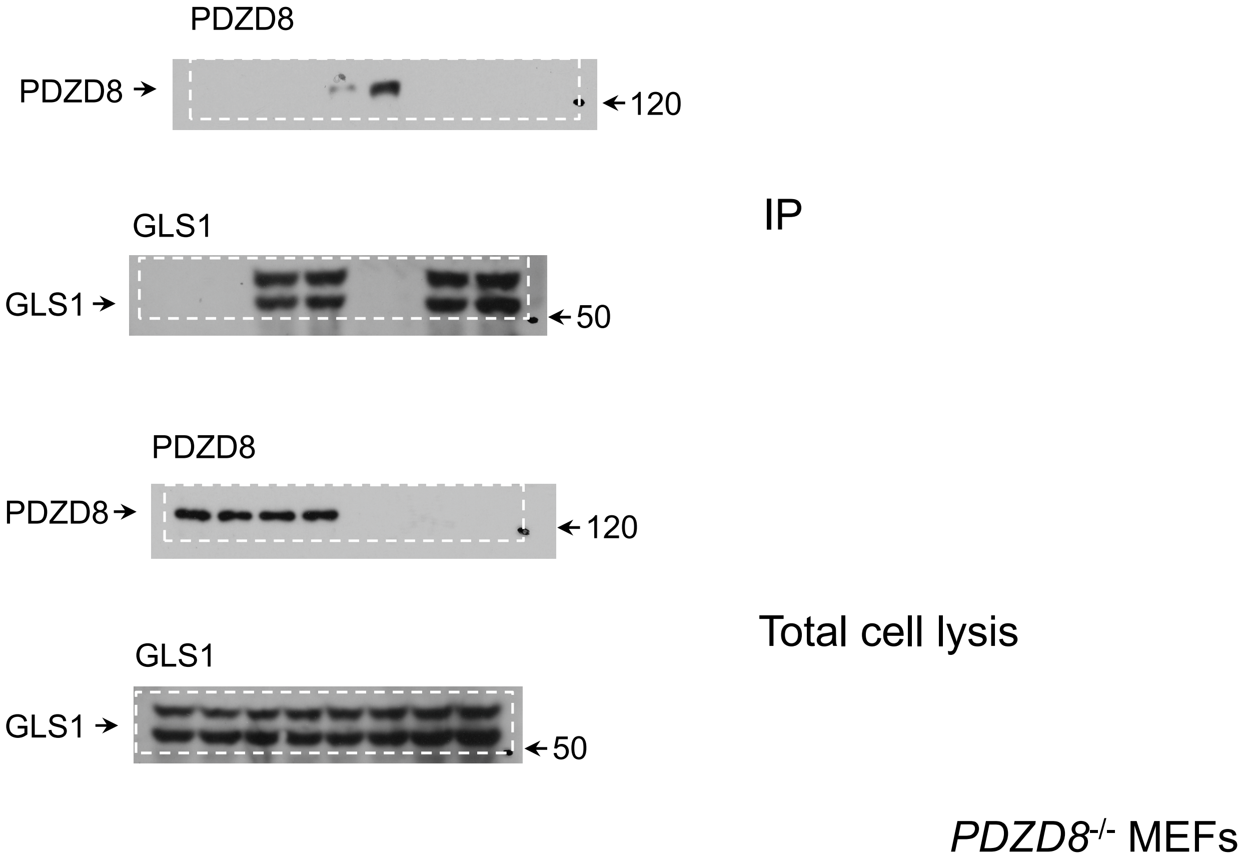
PDZD8
PDZD8
120
IP
GLS1
GLS1
50
PDZD8
PDZD8
120
Total cell lysis
GLS1
GLS1
50
PDZD8-/- MEFs

Fig. 3n

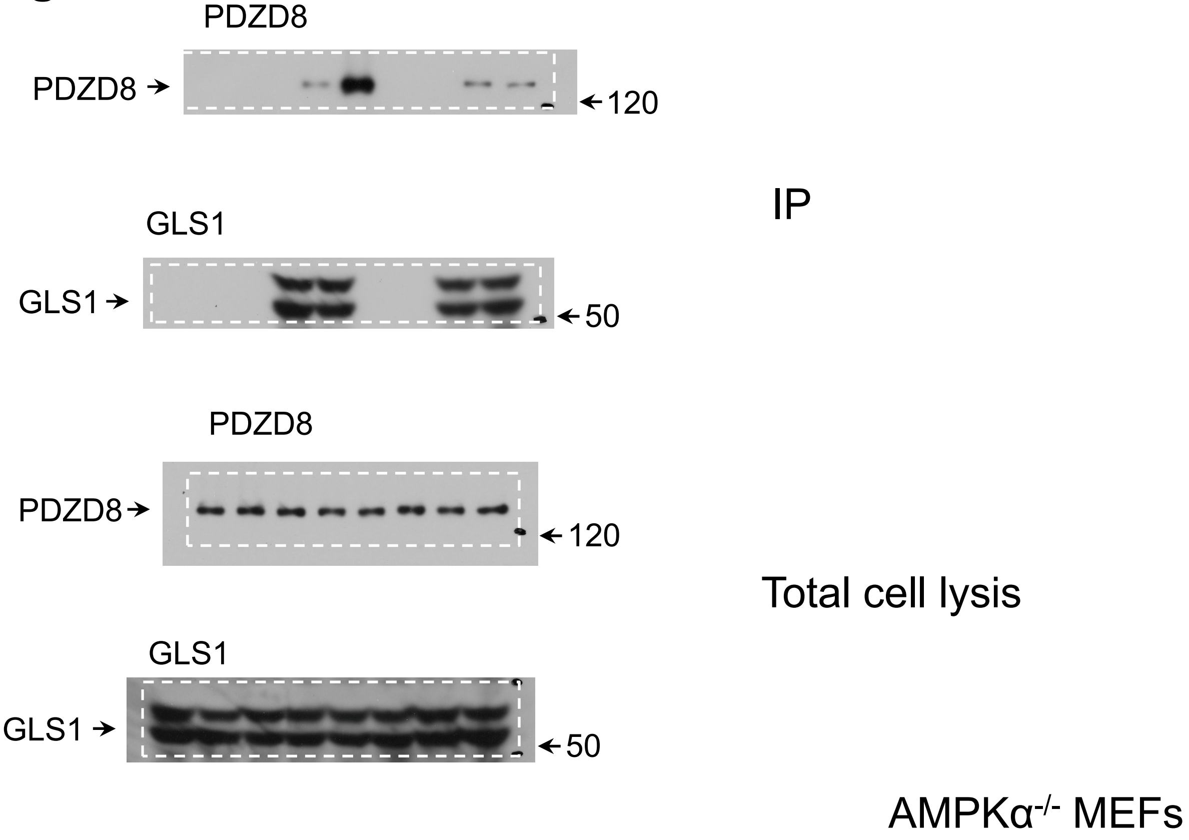
PDZD8
PDZD8
120
IP
GLS1
GLS1
50
PDZD8
PDZD8
120
Total cell lysis
GLS1
GLS1
50
AMPKα-/- MEFs

Fig. 3o

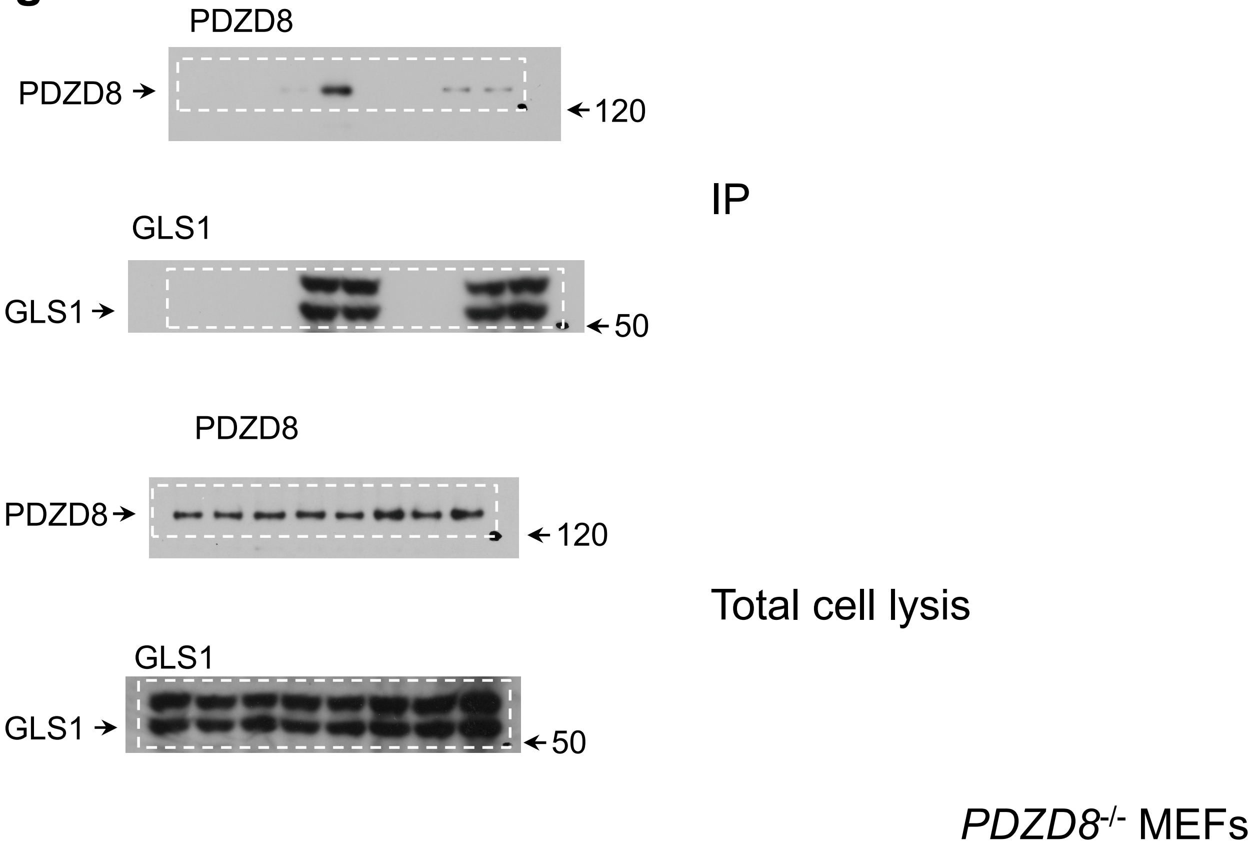

Fig. 3r up

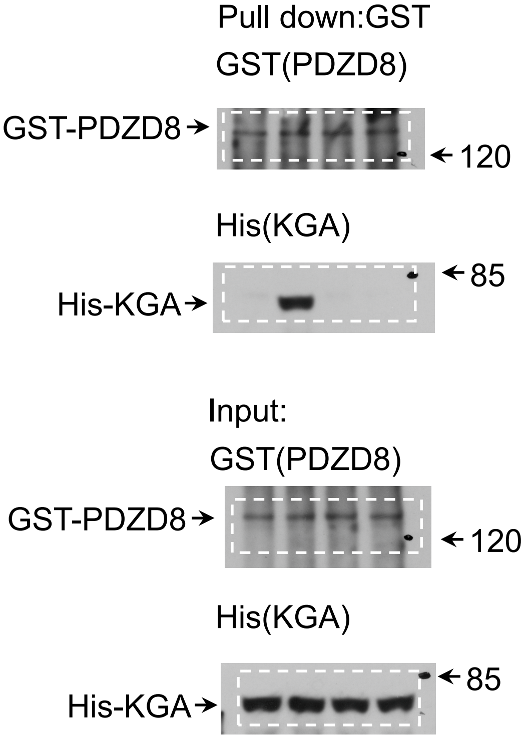

Fig. 3p bottom

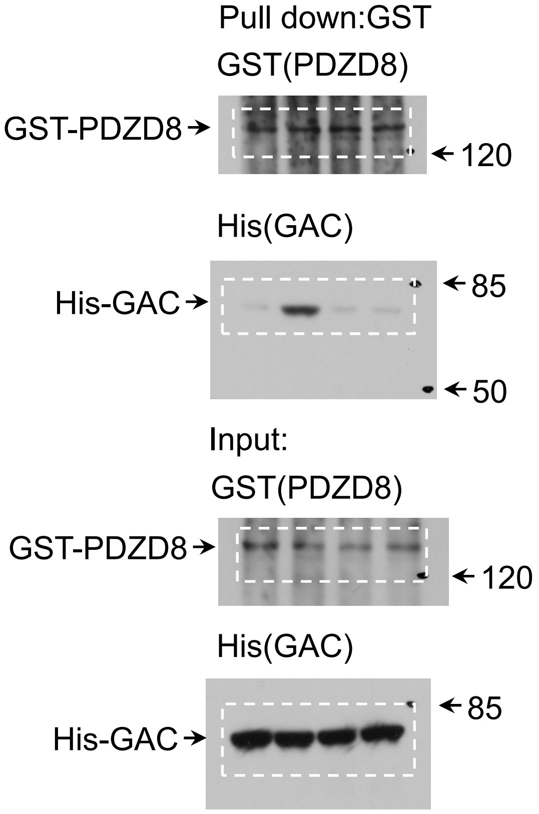

Fig. 4e

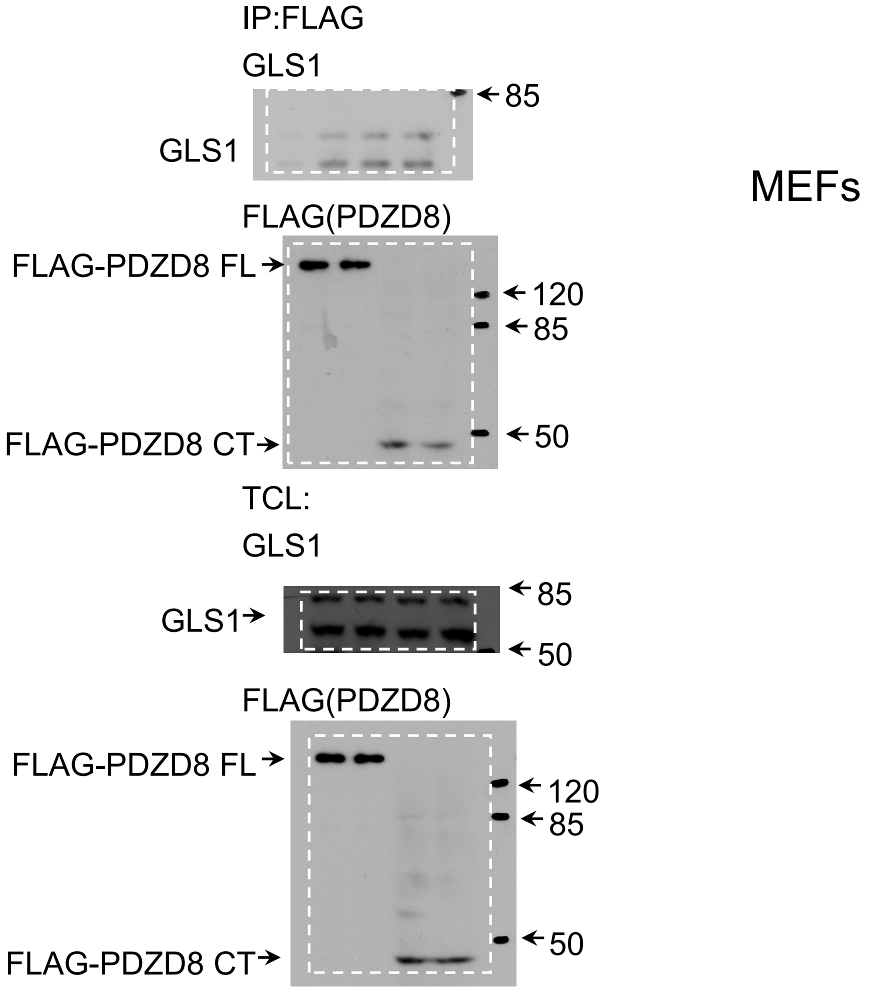
IP:FLAG
GLS1
GLS1
85
MEFs
FLAG(PDZD8)
FLAG-PDZD8 FL
120
85
FLAG-PDZD8 CT
50
TCL:
GLS1
GLS1
85
50
FLAG(PDZD8)
FLAG-PDZD8 FL
120
85
FLAG-PDZD8 CT
50

Fig. 5f

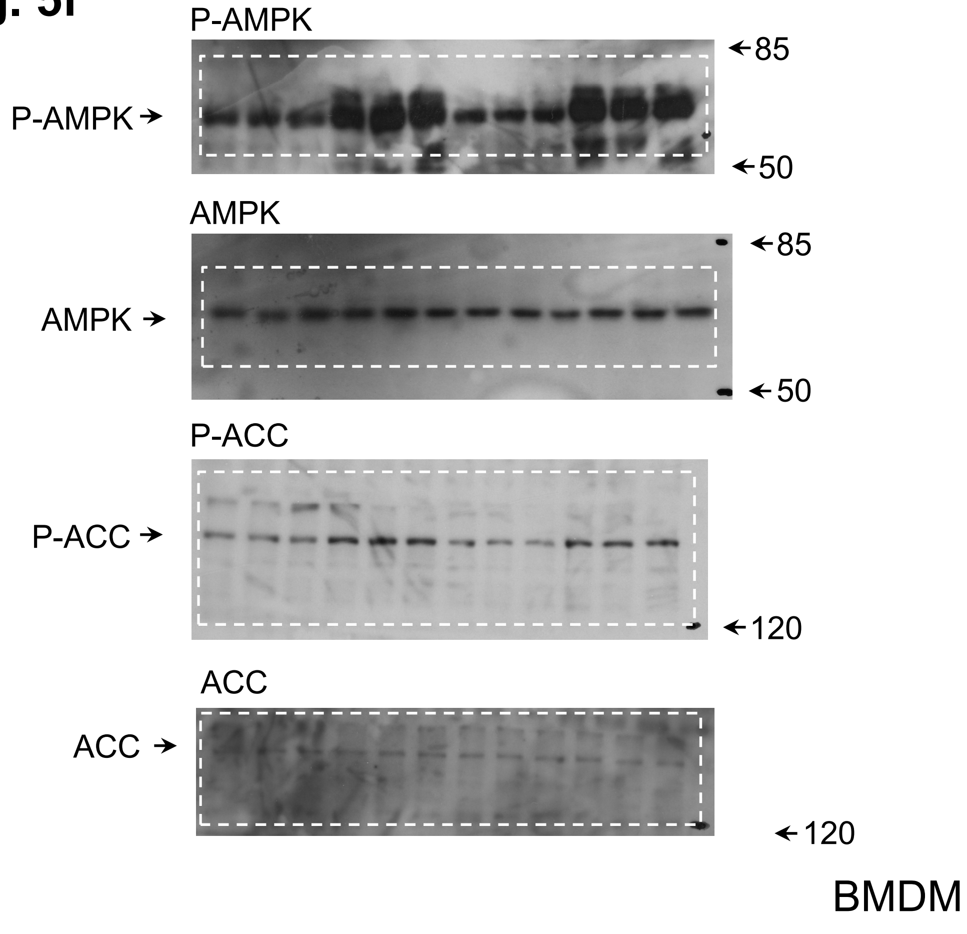
P-AMPK
P-AMPK
85
50
AMPK
AMPK
85
50
P-ACC
P-ACC
120
ACC
ACC
120
BMDM

Fig. S1a

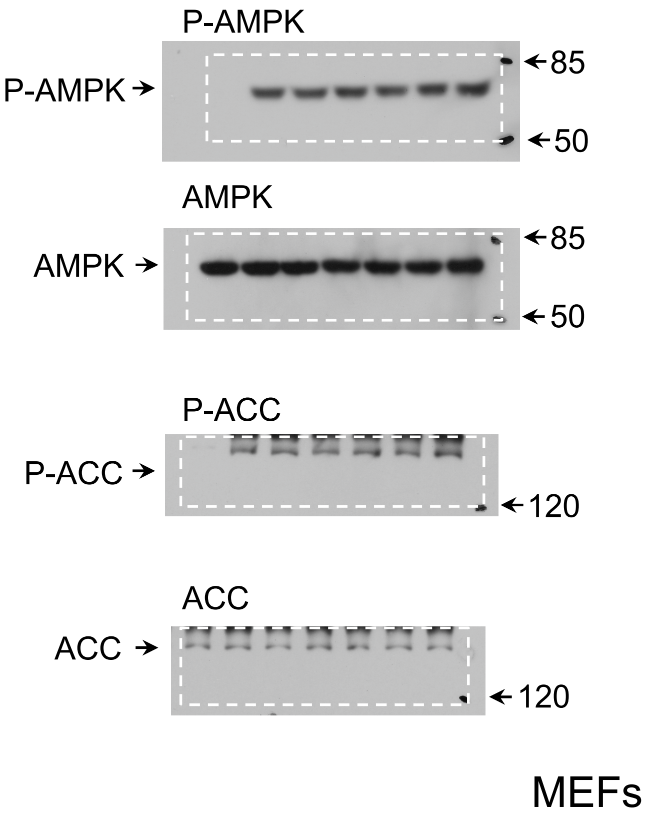
P-AMPK
P-AMPK
85
50
AMPK
AMPK
85
50
P-ACC
P-ACC
120
ACC
ACC
120
MEFs

Fig. S3a left

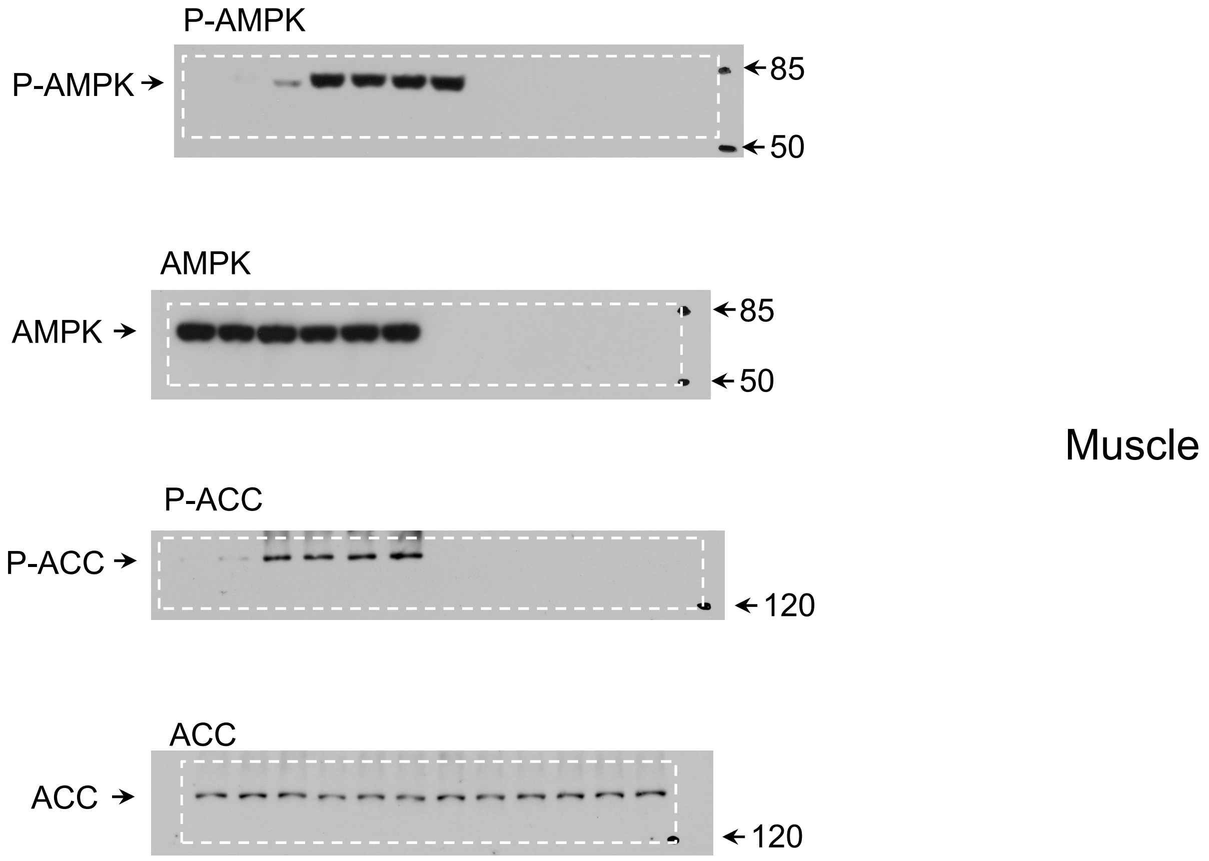

Fig. S3a right

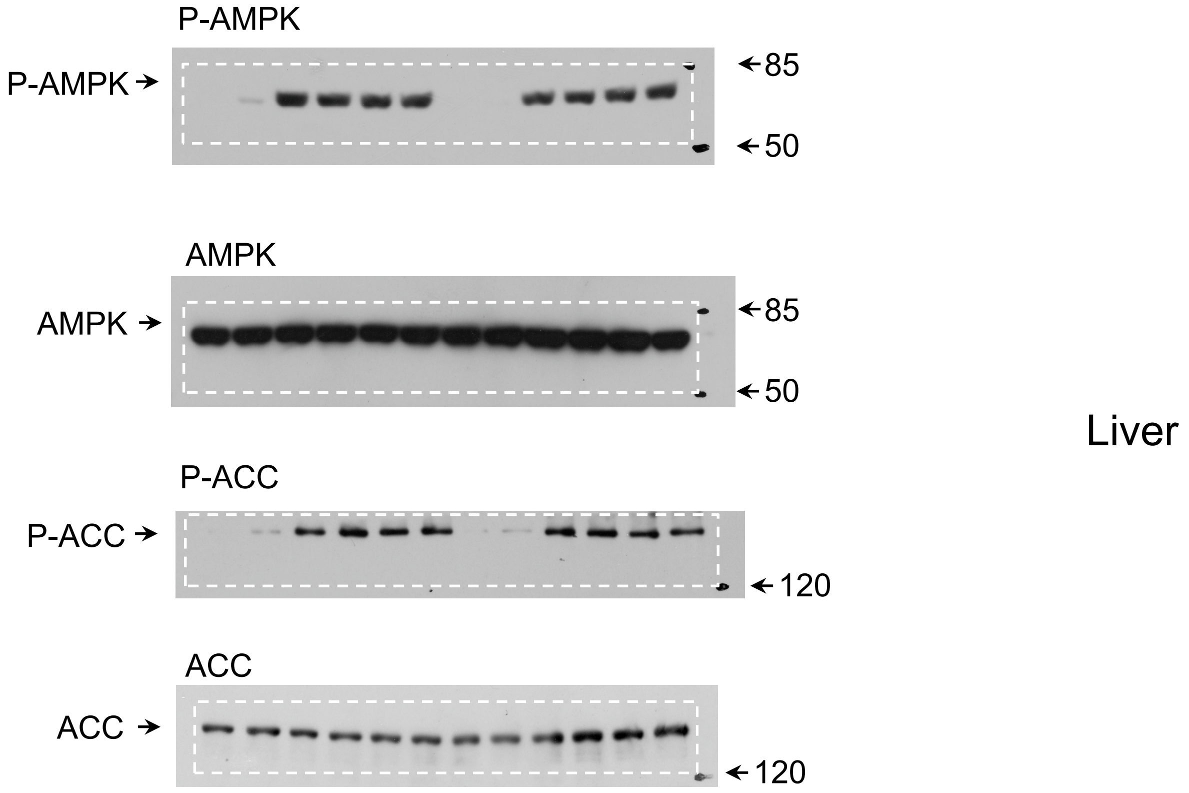

Fig. S3g left

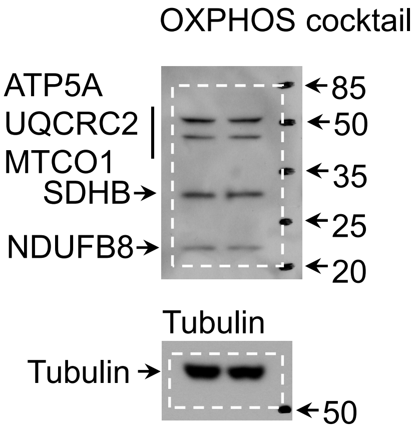

MEFs

Fig. S3g right

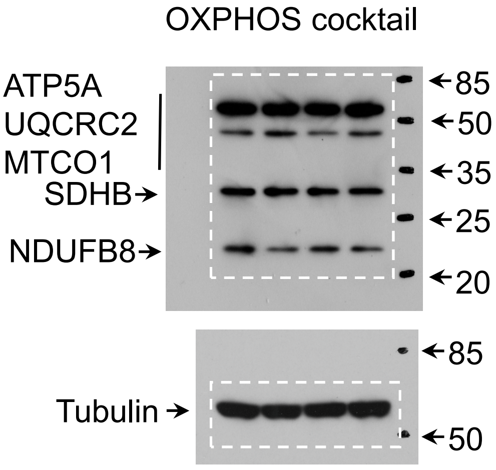

Muscle

Fig. S4a

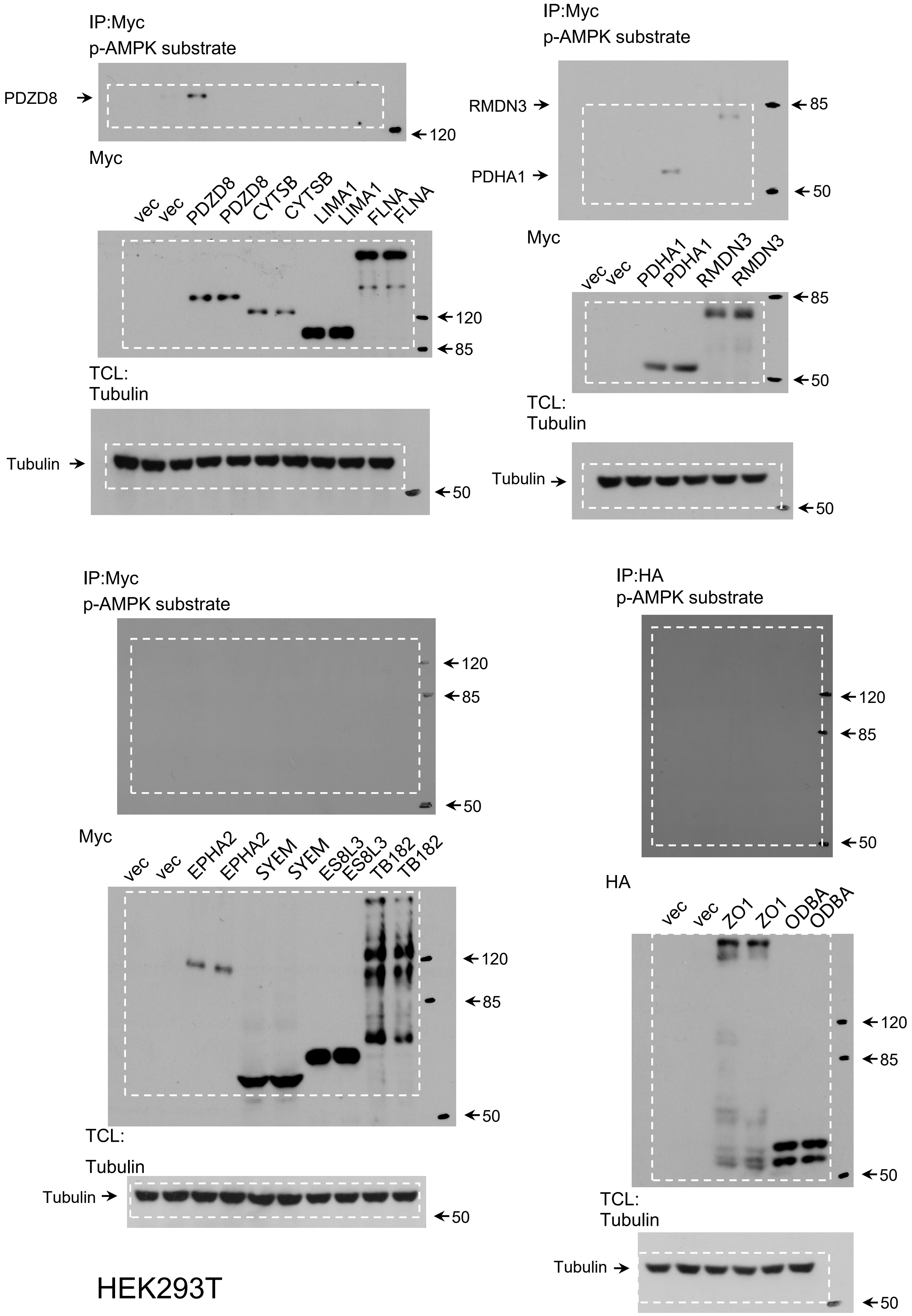
IP:Myc
p-AMPK substrate
PDZD8
120
Myc
vec
vec
PDZD8
PDZD8
CYTSB
CYTSB
LIMA1
LIMA1
FLNA
FLNA
120
85
TCL:
Tubulin
Tubulin
50
IP:Myc
p-AMPK substrate
RMDN3
PDHA1
85
50
Myc
vec
vec
PDHA1
PDHA1
RMDN3
RMDN3
85
50
TCL:
Tubulin
Tubulin
50
IP:Myc
p-AMPK substrate
120
85
50
Myc
vec
vec
EPHA2
EPHA2
SYEM
SYEM
ES8L3
ES8L3
TB182
TB182
120
85
50
TCL:
Tubulin
Tubulin
50
IP:HA
p-AMPK substrate
120
85
50
HA
vec
vec
ZO1
ZO1
ODBA
ODBA
120
85
50
TCL:
Tubulin
Tubulin
50
HEK293T

Fig. S4a

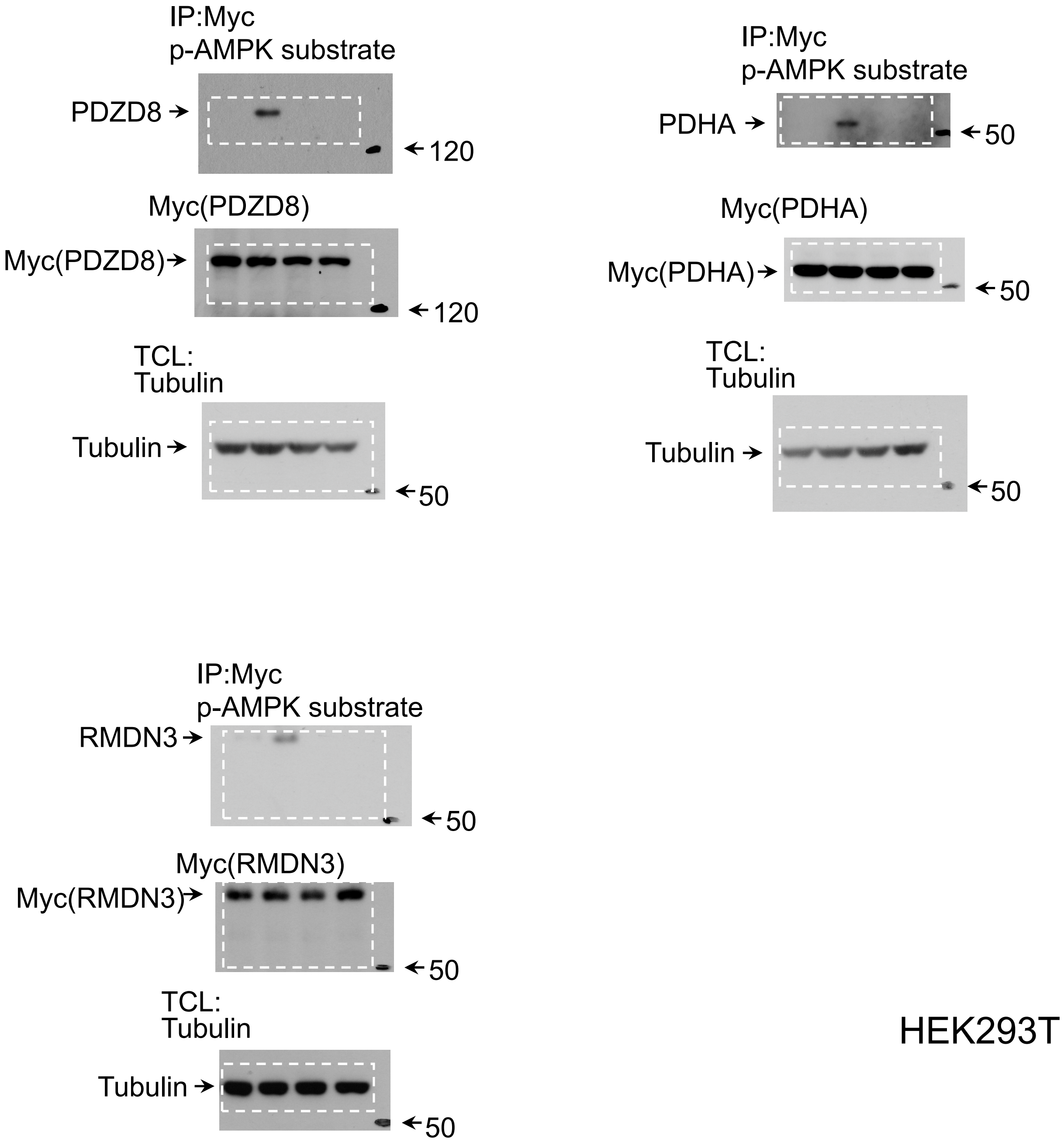
IP:Myc
p-AMPK substrate
PDZD8
120
Myc(PDZD8)
Myc(PDZD8)
120
TCL:
Tubulin
Tubulin
50
IP:Myc
p-AMPK substrate
PDHA
50
Myc(PDHA)
Myc(PDHA)
50
TCL:
Tubulin
Tubulin
50
IP:Myc
p-AMPK substrate
RMDN3
50
Myc(RMDN3)
Myc(RMDN3)
50
TCL:
Tubulin
Tubulin
50
HEK293T

Fig. S4b left

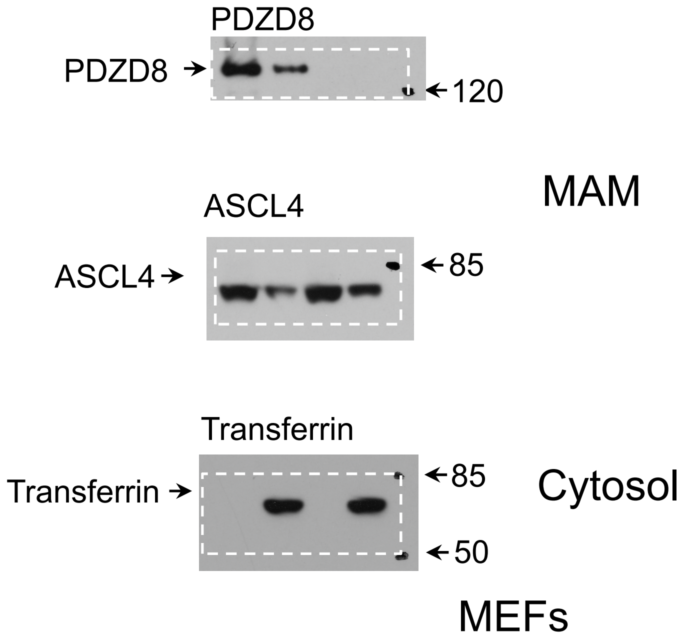

Fig. S4b middle

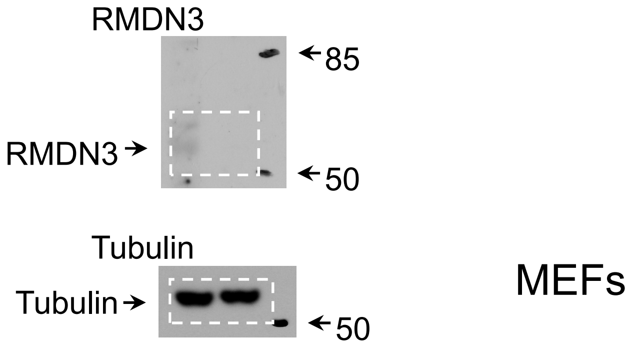

Fig. S4b right

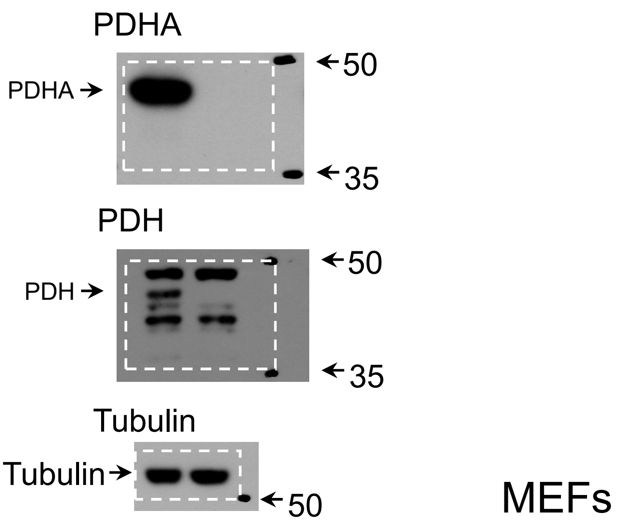

Fig. S4g

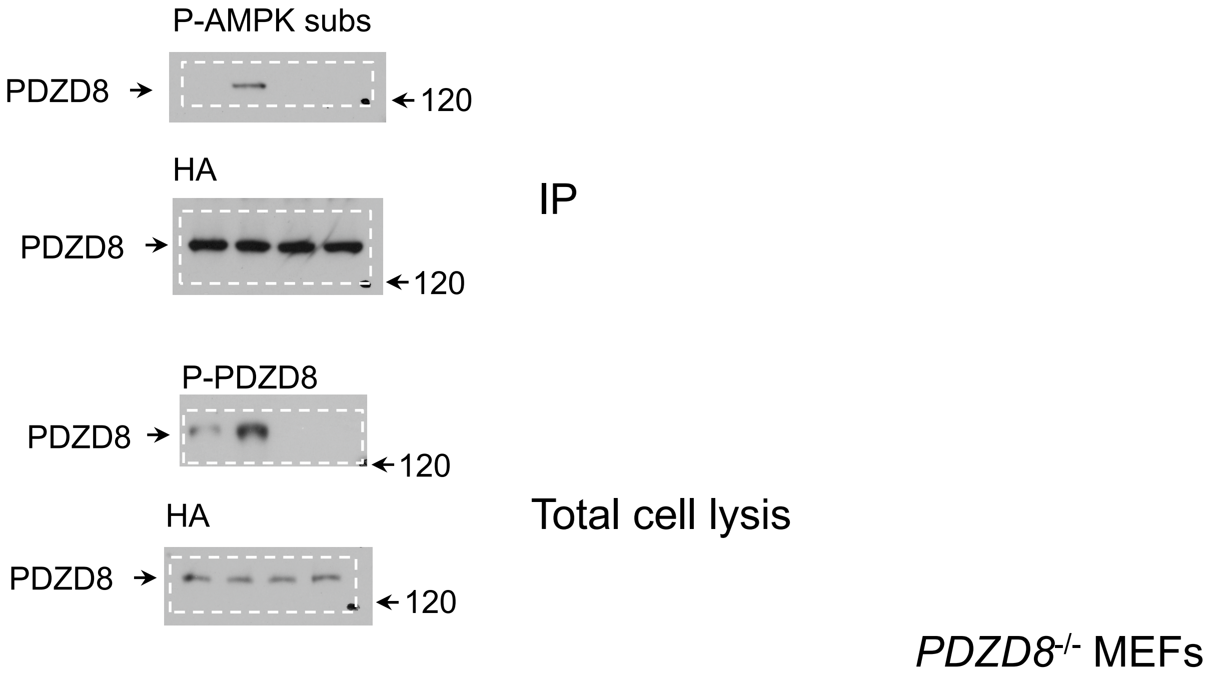

P-AMPK subs
PDZD8
120
HA
PDZD8
120
IP
P-PDZD8
PDZD8
120
Total cell lysis
HA
PDZD8
120
PDZD8-/- MEFs

Fig. S5c left

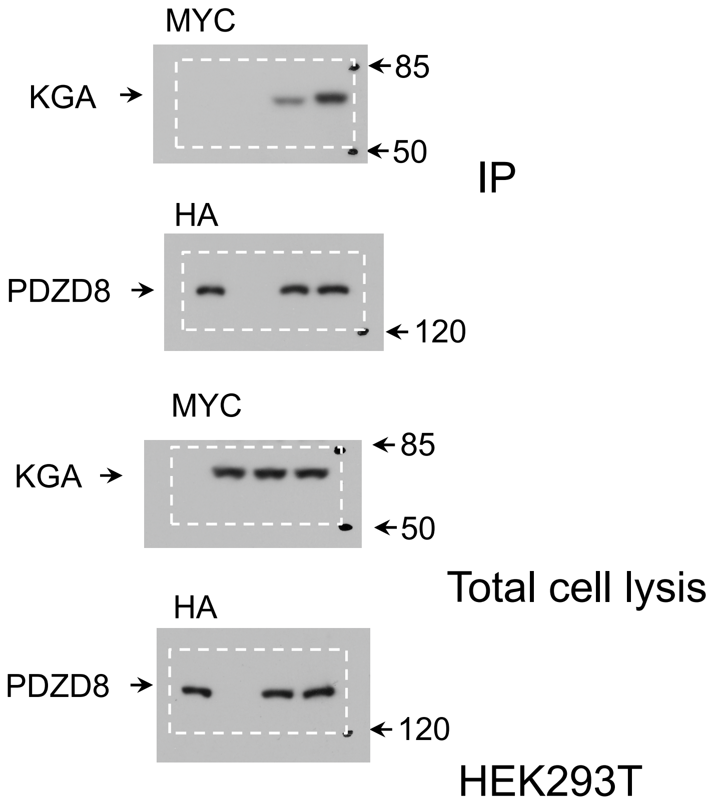

Fig. S5c right

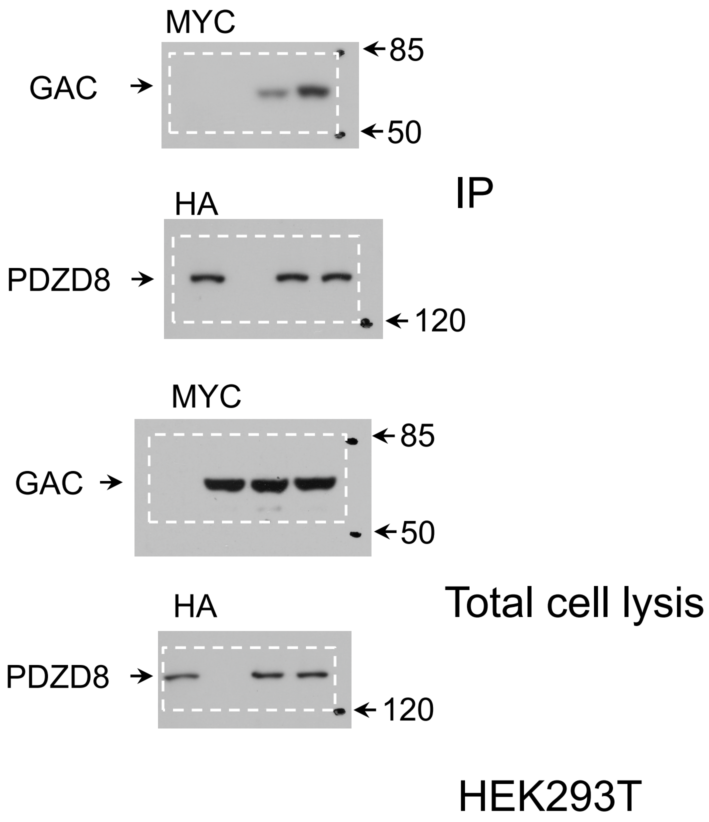

Fig. S5d up

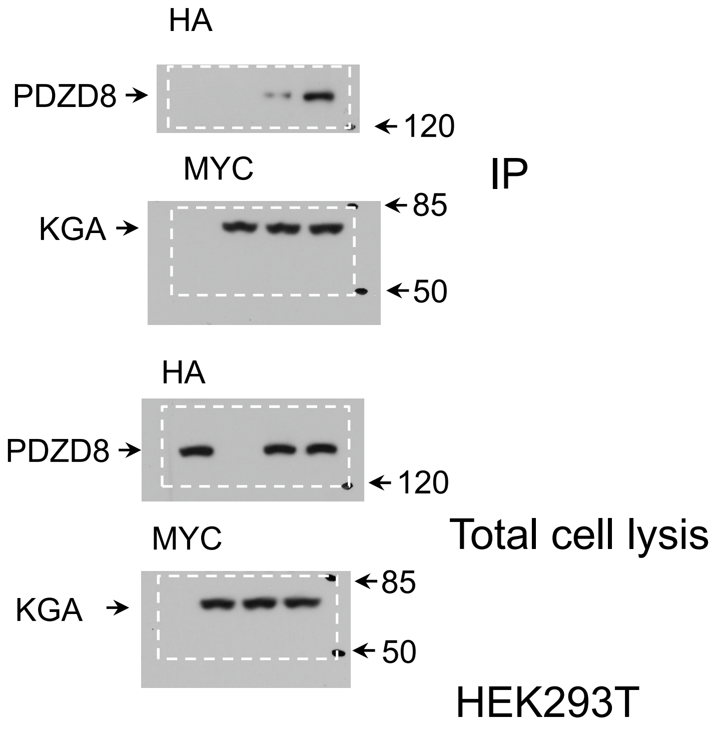

Fig. S5d bottom

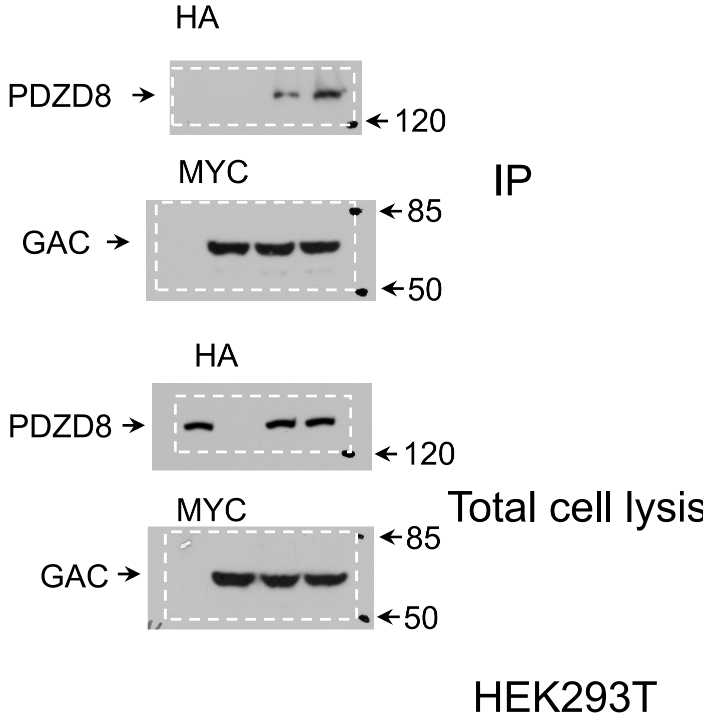

Fig. S5f left

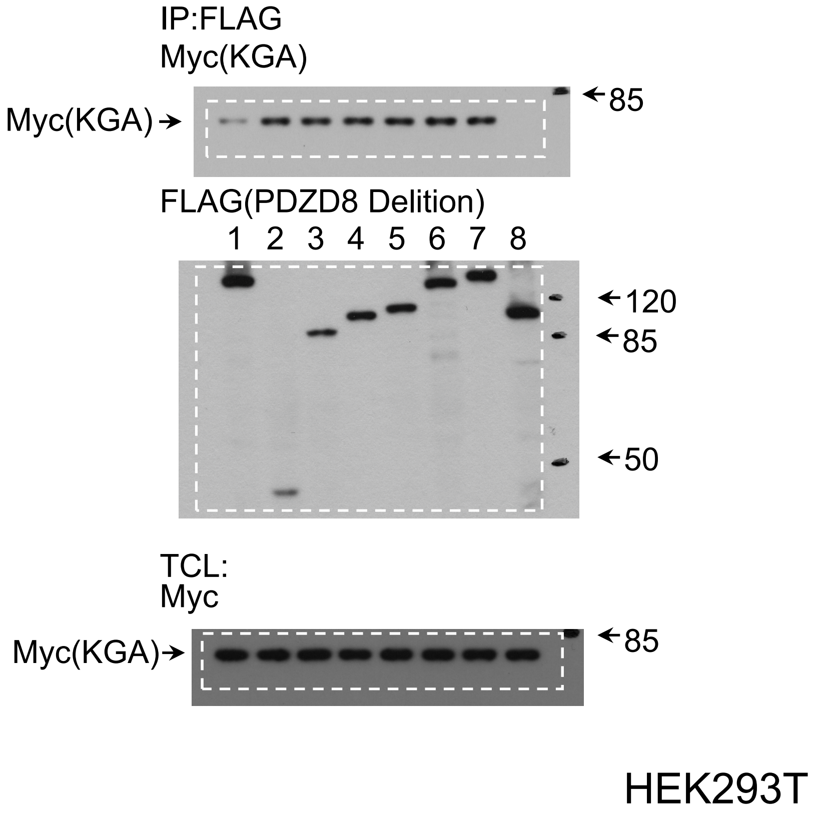

Fig. S5f right

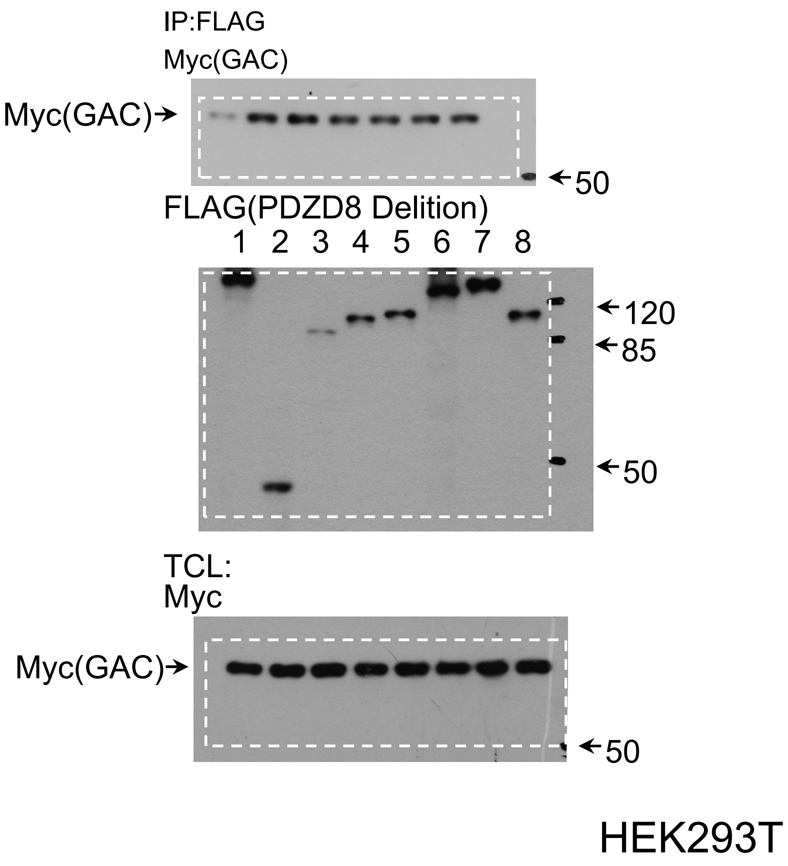

Fig. S5h up

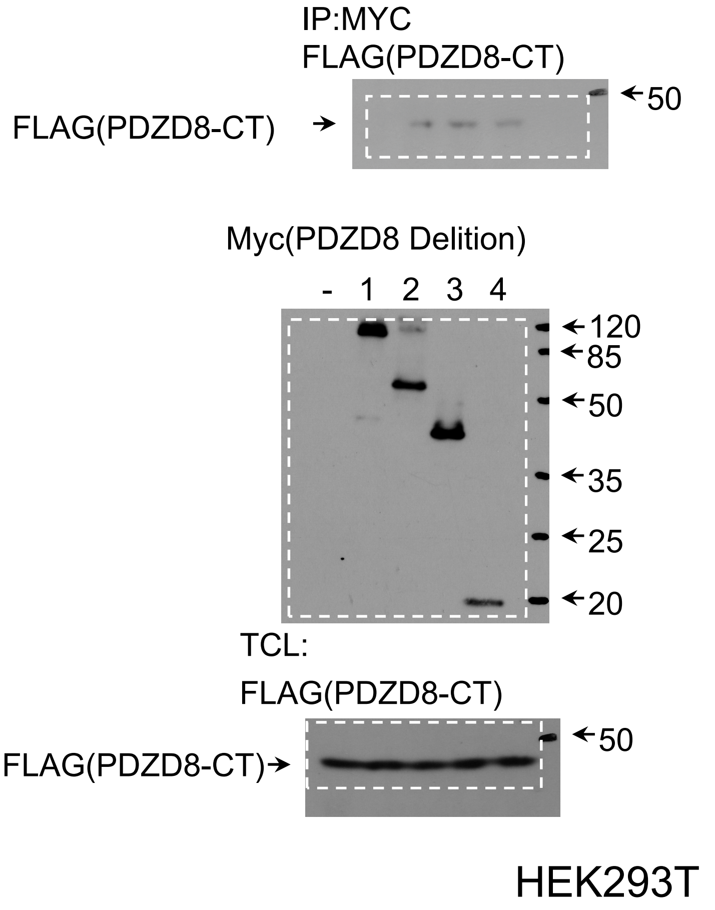

Fig. S5h middle

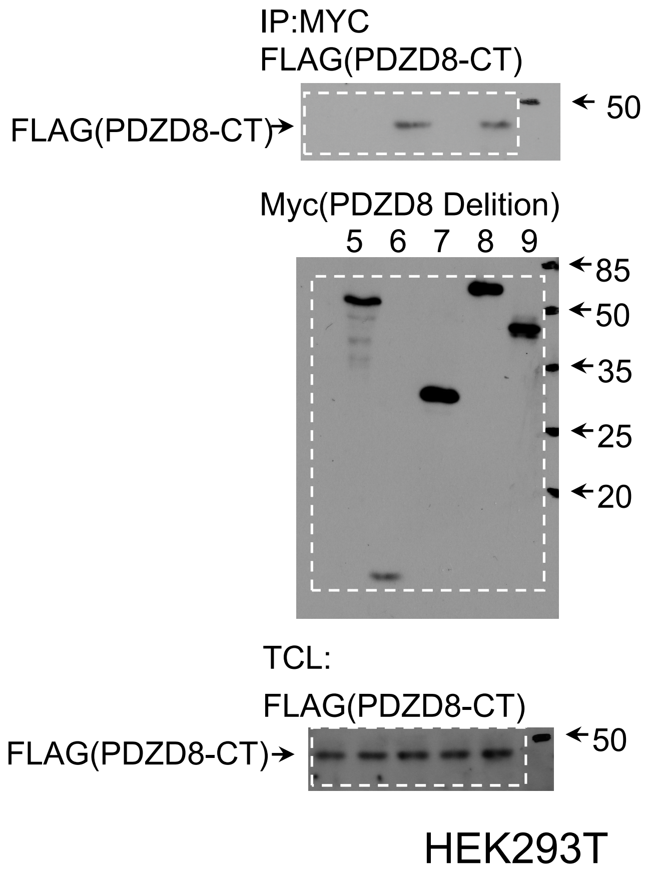

Fig. S5h bottom

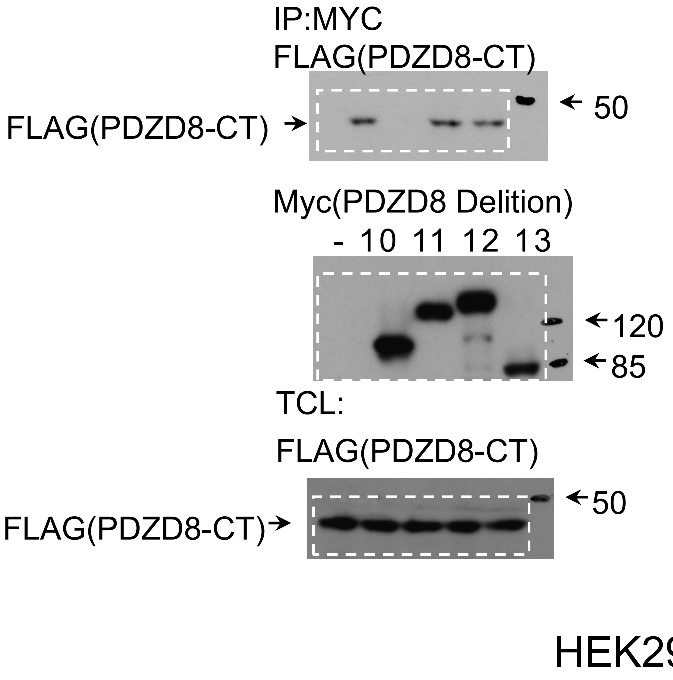

Fig. S5i up

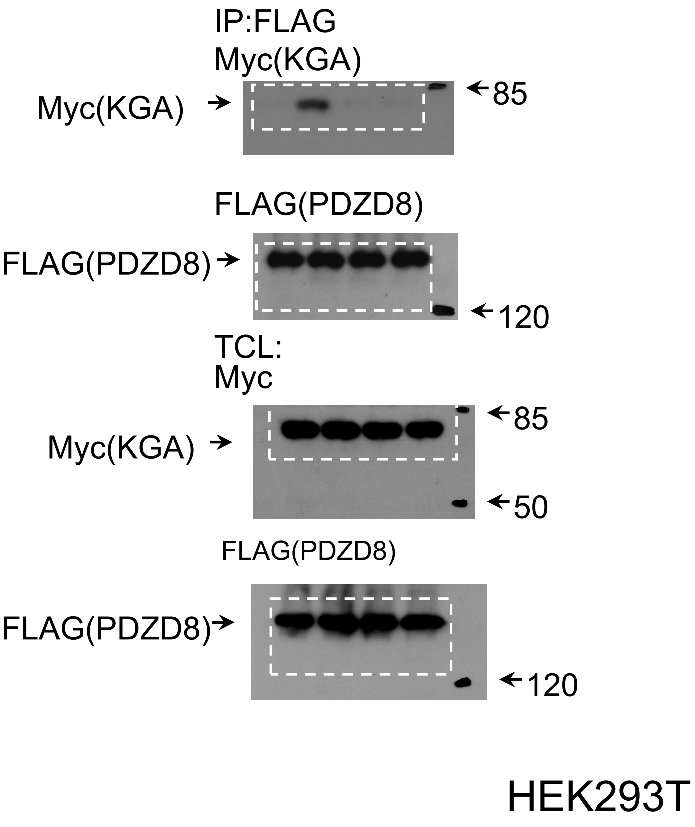

Fig. S5i bottom

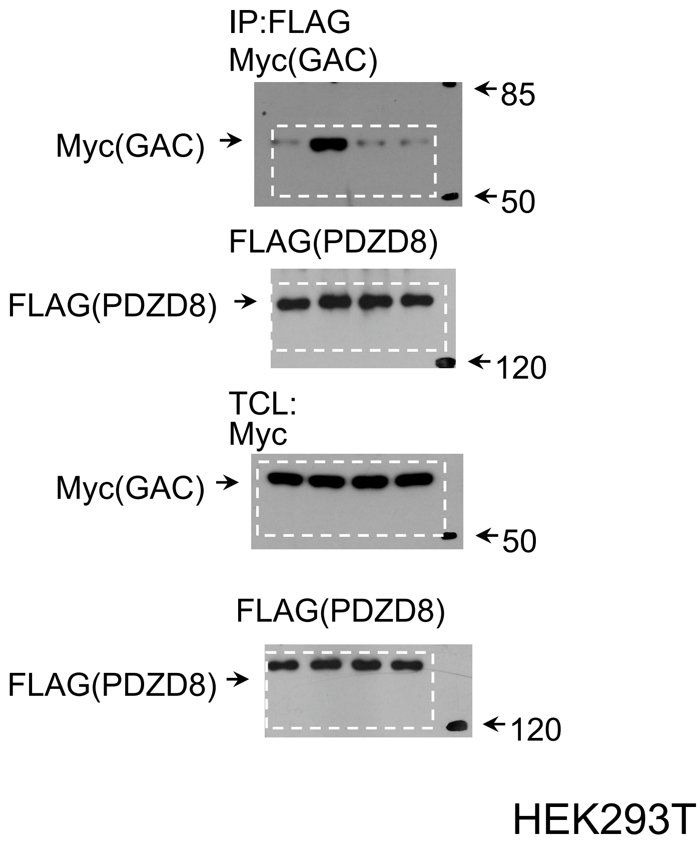

Fig. S6b (up)

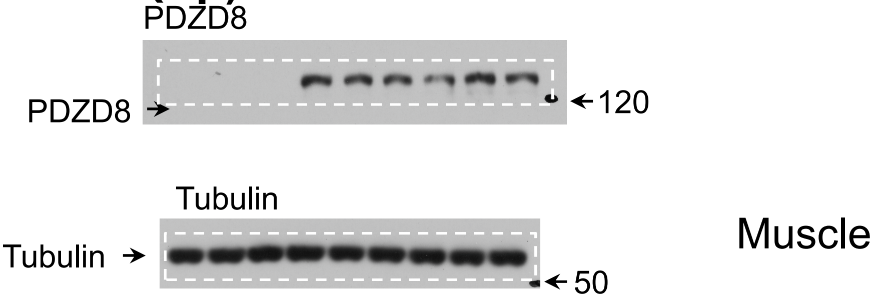

Fig. S6b(down)

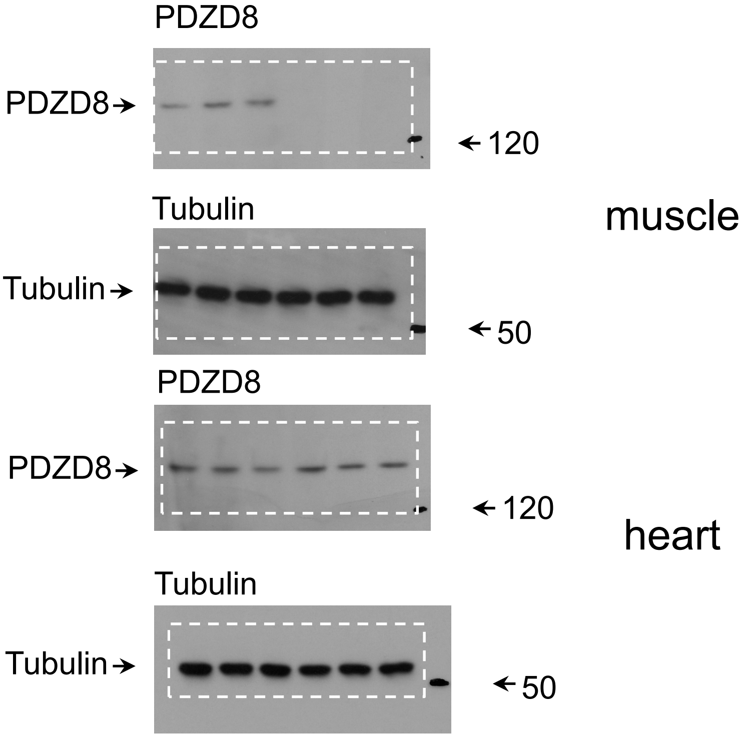

Fig. S7a

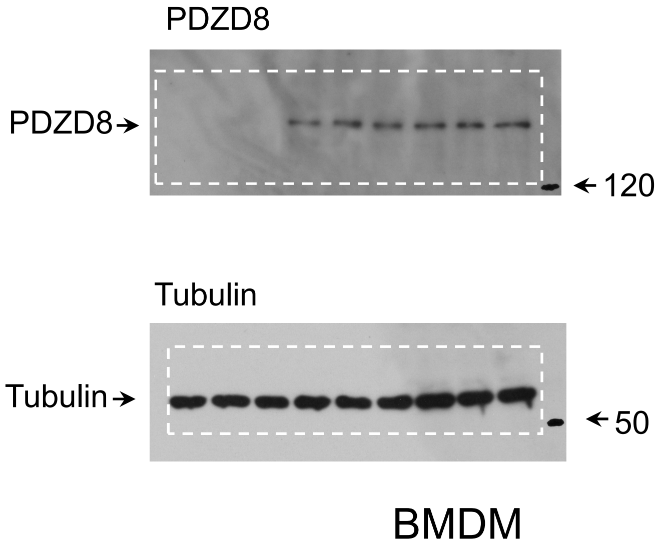

Fig. S7k left

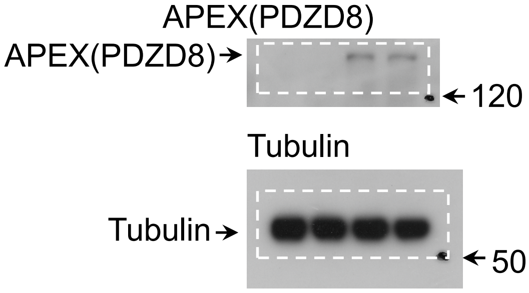

Fig. S7k right

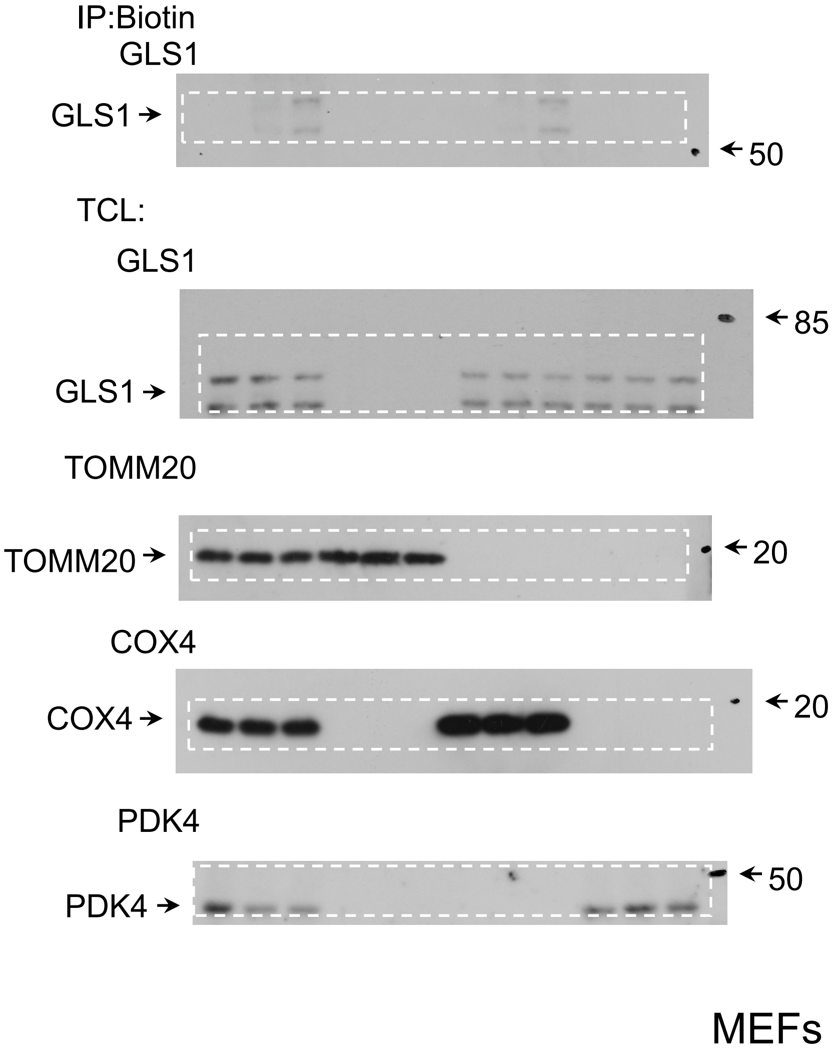

Fig. S7j

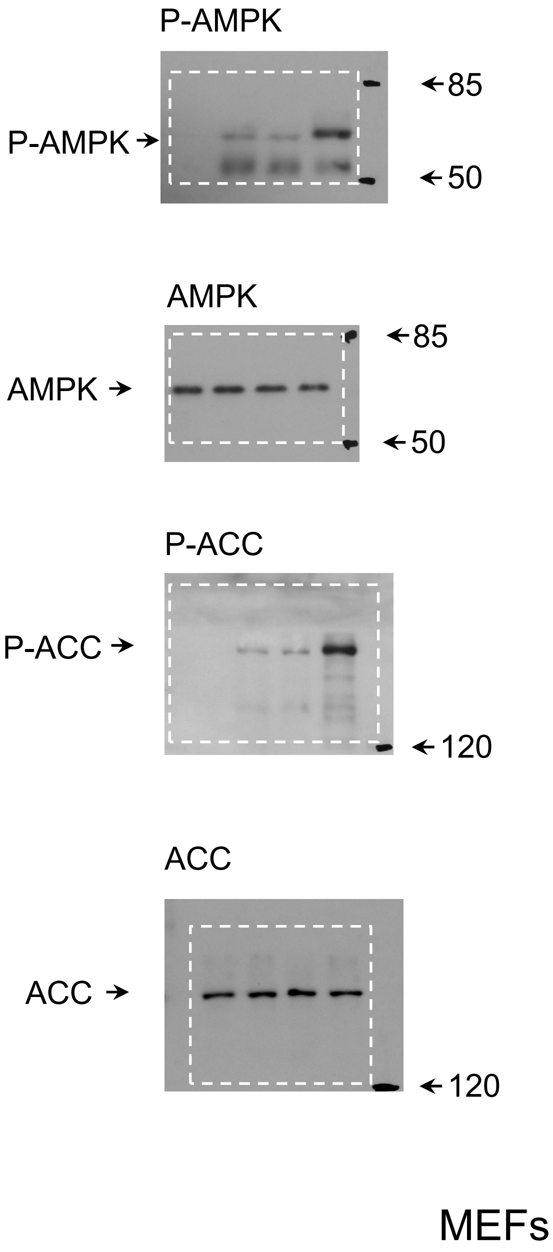

P-AMPK
P-AMPK
85
50
AMPK
AMPK
85
50
P-ACC
P-ACC
120
ACC
ACC
120
MEFs

Fig. S7I left

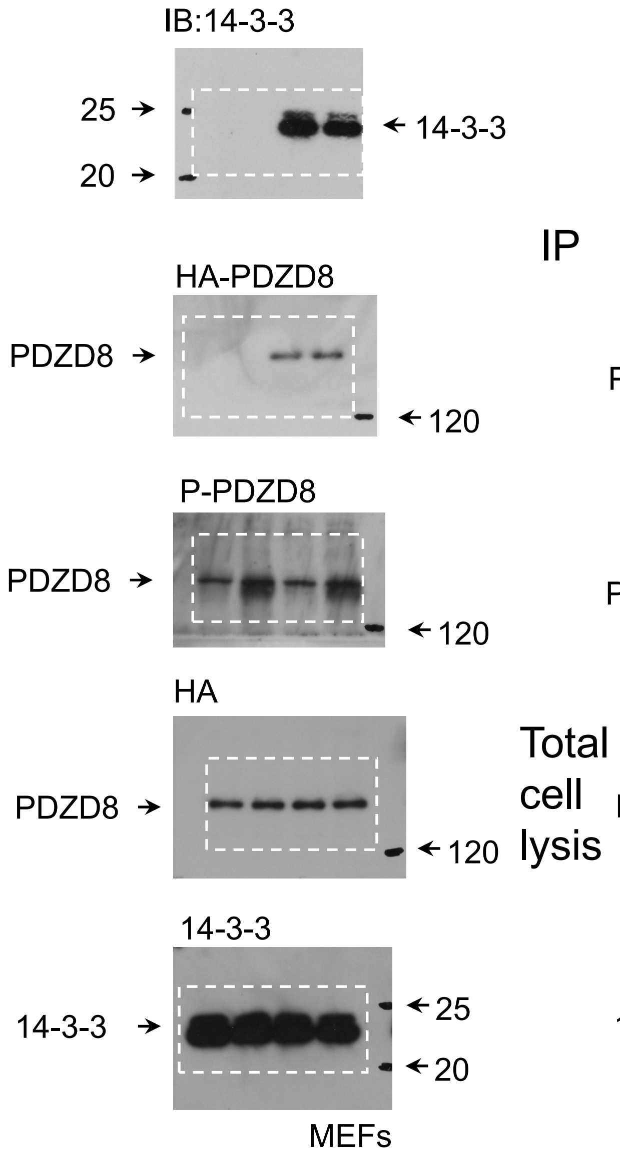

Fig. S7I right

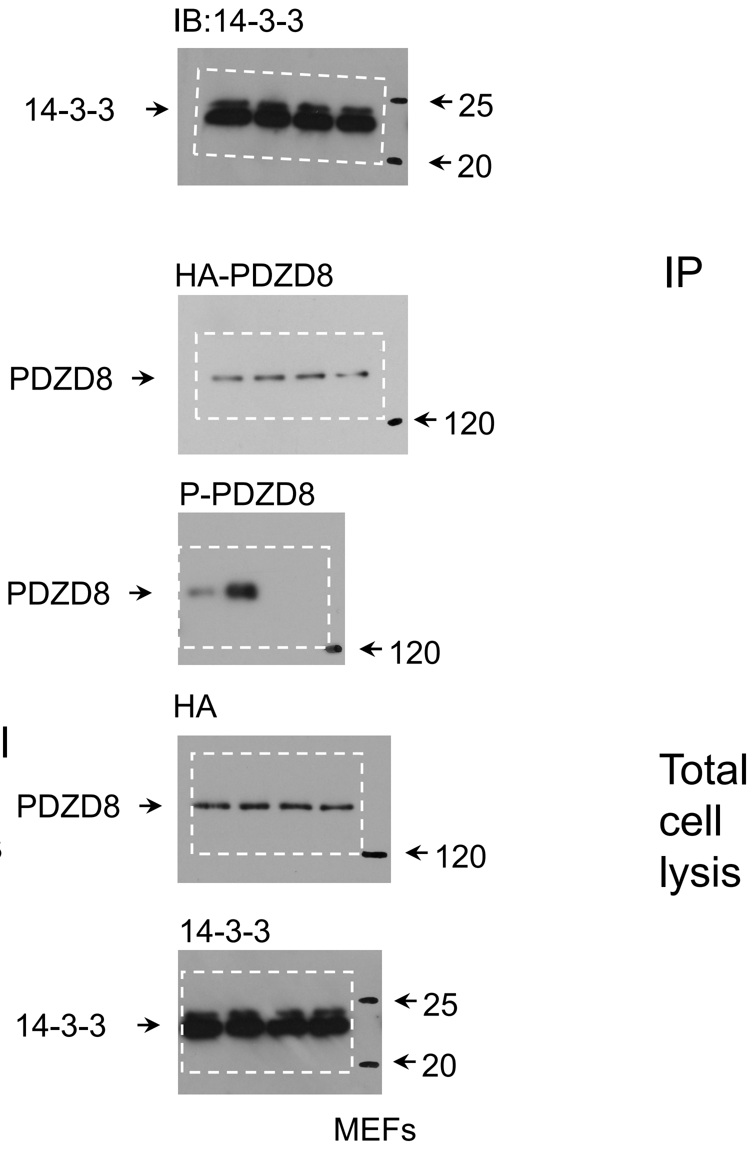

Supplement: Supplementary file 12 — Full scans [file 41422_2024_985_MOESM12_ESM.pdf]
